# Risk mitigation services in cyber insurance: optimal contract design and price structure

**DOI:** 10.1057/s41288-023-00289-7

**Published:** 2023-05-08

**Authors:** Gabriela Zeller, Matthias Scherer

**Affiliations:** Fakultät für Mathematik, Lehrstuhl für Finanzmathematik, Technische Universität München, Parkring 11, 85748 Garching, Germany

**Keywords:** Cyber risk, Cyber insurance, Cyber assistance, Prevention, Self-protection, Self-insurance, Coherent risk measures, Stackelberg game

## Abstract

As the cyber insurance market is expanding and cyber insurance policies continue to mature, the potential of including pre-incident and post-incident services into cyber policies is being recognised by insurers and insurance buyers. This work addresses the question of how such services should be priced from the insurer’s viewpoint, i.e. under which conditions it is rational for a profit-maximising, risk-neutral or risk-averse insurer to share the costs of providing risk mitigation services. The interaction between insurance buyer and seller is modelled as a Stackelberg game, where both parties use distortion risk measures to model their individual risk aversion. After linking the notions of pre-incident and post-incident services to the concepts of self-protection and self-insurance, we show that when pricing a single contract, the insurer would always shift the full cost of self-protection services to the insured; however, this does not generally hold for the pricing of self-insurance services or when taking a portfolio viewpoint. We illustrate the latter statement using toy examples of risks with dependence mechanisms representative in the cyber context.

**Supplementary Information:**

The online version contains supplementary material available at 10.1057/s41288-023-00289-7.

## Introduction

### Motivation and approach

Cyber insurance is still a relatively new, but steadily expanding market. The reasons for its ongoing growth in demand are manifold: the dynamically expanding and evolving cyber-threat landscape (ENISA 2021; tenable 2021), extensive media coverage of severe cyber incidents (Advisen and PartnerRe 2017, 2018; Marotta et al. 2017), ubiquitous introduction of stricter legislation (Anchen and Pain 2017; Marotta et al. 2017), and increased awareness of companies about their augmented dependence on information technology. To emphasise the first point, in particular the growing extent of the professionalism and economic potential of the ransomware “industry” are addressed, e.g. in ENISA (2021). As of 2020, cyber incidents were ranked the number one peril to businesses worldwide (Allianz 2020) and their perilousness can hardly be expected to have diminished since, as the COVID-19 pandemic and its effects (e.g. extensive ad-hoc shifts to remote work without adequate time to amend IT security measures and practices) have been labelled by some experts “the largest-ever cybersecurity threat” (Munich Re 2021). Many insurers are already actively participating in the global cyber insurance market, while still grappling with a firm understanding of this new and dynamic type of risk and its underlying drivers. Far from being solved is the question of how to adequately assess and price cyber risk given the various challenges, e.g. scarcity of historical data, non-stationarity of claims, association between claims, and strategic motivations of threat actors. Many academic works have recently been devoted to understanding and modelling these challenges in cyber risk. We, therefore, deliberately refrain from providing an exhaustive overview and refer to the surveys (Marotta et al. 2017; Awiszus et al. 2023).

In most established insurance lines, insurers have multiple years of claims experience and established technical expertise to quantify risks. In contrast, assessing and pricing cyber risks is particularly challenging due to the dynamically evolving threat landscape and the high complexity of modern IT systems. Therefore, insurers strive to collaborate with specialised IT security service providers (consider Bosch CyberCompare as an example or Advisen for a market overview), who not only support insurers in accurately assessing to-be-insured risks, but collaborate in providing services that aim at mitigating the insured risk as part of an insurance policy. Such cyber-assistance services can be divided into *pre-incident* services, such as network security, back-up of critical systems and data, and patch management, and *post-incident* services, such as restoration of data, forensic services, and legal advice (see Munich Re 2021). The former typically serve to decrease the probability of a cyber incident, while the latter support mitigation of the loss size in case an incident has occurred. In practice, the effects of both types of service are naturally intertwined, and additionally, all types of cyber assistance can also serve to provide insurers with additional information, i.e. to enhance their cyber-risk assessment practices or simply to obtain supplementary data (see also Remark 1 below). A recent survey (Munich Re 2021) indicates that the majority of (prospective) buyers believes that such services should be covered by holistic cyber insurance solutions, indicating that both the supply and demand side have realised that cyber insurance coverage should encompass more than pure compensation for financial losses. One type of service which is not yet explicitly advertised on the market, but holds great potential, is the insurer’s ability to use the interdependence of cyber incidents to all parties’ benefit by offering additional risk mitigation services.

To the best of our knowledge, established actuarial pricing approaches for these new policies are yet to be developed. The aim of this work is to propose a mathematical framework to study the optimal price structure of such insurance contracts, in particular to start addressing the question if (and under which circumstances) an insurer is economically incentivised to subsidise risk reduction services within an insurance policy. As part of this question, the issue of the optimal combination of insurance and risk mitigation (depending on their prices) from an insurance buyer’s point of view is also studied. A further point, which is particularly relevant in the cyber context, is that for an insurer, it is not exhaustive to consider every single policyholder separately, but due to the potential interconnectedness of cyber losses, a portfolio viewpoint considering dependencies needs to be taken into account.

Our approach is based on the work of Bensalem et al. (2020), by using the framework of distortion risk measures and stochastic ordering of loss distributions, respectively, to capture risk assessment of all parties and the effects of risk mitigation services, and by modelling the interaction between insurer and insurance buyer(s) as a Stackelberg game. We extend their setting to a bivariate problem for the insurer, allowing her to choose the price for both risk transfer and risk mitigation, and analyse the results of the corresponding buyer’s problem [which is conceptually similar to Bensalem et al. (2020)] in the cyber insurance context. Furthermore, we transcend from the study of an interaction with a single buyer to examples of (sequential or simultaneous) interactions with several buyers with dependent losses.

### Related literature

A concise overview of academic studies on the interaction between risk reduction and insurance in the cyber context is given in Xiang et al. (2021). As mentioned therein, many of these studies rely on very simplified assumptions regarding the distribution of random cyber losses or the interplay between costs of prevention and consequence on the reduction of risk. Most often, the optimal combination of security provisions and insurance from an insured’s point of view is studied, see, e.g. the early game-theoretic contribution of Pal and Golubchik (2010), the work of Young et al. (2016), and subsequently Mazzoccoli and Naldi (2020), or Yang and Lui (2014), Chase et al. (2017), and Mazzoccoli and Naldi (2021) who investigate optimal security investments under the presence of cyber insurance in a heterogeneous network, in a cloud computing environment, and for a multi-branch firm with correlated vulnerabilities, respectively. Zhang and Zhu (2021) use a dynamic moral hazard type of principal–agent model with Markov decision processes to capture decisions on self-protection of the insured and Skeoch (2022) expands the Gordon–Loeb model (Gordon and Loeb 2002) for cybersecurity to a cyber insurance context. Pal et al. (2014, 2017) more generally study synergies between cybersecurity and the (existence of a then nascent) cyber insurance market.

Fewer studies emphasise the insurer’s role in designing cyber insurance contracts, e.g. by choosing premium and contractual indemnity (Dou et al. 2020), employing a bonus-malus system (Xiang et al. 2021), or trying to mitigate moral hazard by means of risk preference design (Liu and Zhu 2022).

The problem of combining different strategies of coping with risk, in particular the combination of risk reduction by investing in prevention measures and risk transfer by purchasing insurance, is of course not specific to cyber and has been the interest of many earlier studies. A good starting point is the survey (Courbage et al. 2013) on the economic literature on prevention and precaution. As differentiated therein, prevention activities encompass *self-protection*, i.e. modifying the probability of a loss, and *self-insurance*, i.e. shaping the potential loss size. The seminal work by Ehrlich and Becker (1972) examined the relationship of both activities to market insurance, and many authors have subjected these results to various model changes (for an overview, see Courbage et al. 2013), see, e.g. Dionne and Eeckhoudt (1985) and Hiebert (1989). Most aforementioned models use an *Expected Utility* (EU) framework and consider only two states (i.e. a loss occurs = “bad” state or no loss occurs = “good” state).^1^ Another model of behaviour under risk, namely *Rank Dependent Expected Utility* (RDEU), has been considered for the study of prevention, e.g. in Konrad and Skaperdas (1993), Bleichrodt and Eeckhoudt (2006), Etner and Jeleva (2013). Courbage (2001) considered the relationships between market insurance, self-insurance, and self-protection in the context of Yaari’s Dual Theory.

Our work is conceptually most closely related to Bensalem et al. (2020), who model the interaction between insurer and insurance buyer as a so-called *Stackelberg game* (see, e.g. Osborne and Rubinstein 1994; Fudenberg and Tirole 1991), a setting recently used to describe the interaction between reinsurer(s) and insurer(s), e.g. in Bai et al. (2022), Chen and Shen (2018), Chen et al. (2020), and Cheung et al. (2019).^2^ Recently, some authors have also studied equilibria in sequential optimisation games in an insurance-reinsurance-setting, see, e.g. Boonen and Ghossoub (2022), Boonen et al. (2021) and Boonen and Zhang (2022). Let us also mention that in the cyber insurance domain, some works employ different game-theoretic approaches including the insurer and insured as parties, sometimes additionally featuring malicious third parties (cyber attackers), see, e.g. Zhang et al. (2017) and Yin et al. (2021). One aspect of the usual (principal-agent)problem between an insurer (acting as principal) and an insurance buyer (responding as agent) is the problem of *moral hazard*, i.e. the fact that the (risk reduction) actions of the agent are unobservable to the principal (see, e.g. Holmstrom 1979). This complicates matters, i.e. static principal-agent problems involving moral hazard are typically hard to solve (see, e.g. Rogerson 1985; Jewitt 1988). Many of the above-mentioned works incorporate, or at least mention, the issue of asymmetric information in their studies, e.g. Liu and Zhu (2022), Boonen et al. (2021), and Zhang and Zhu (2021).^3^

The popular framework of risk measures to model risk preferences of both the insurance buyer and insurer has recently been used by, e.g. Bensalem et al. (2020), Cheung et al. (2019), Boonen and Ghossoub (2022), and Balbás et al. (2011), mostly in an insurance-reinsurance context. In the insurance context, an axiomatic characterisation of insurance prices as Choquet integrals (see Denneberg 2013) with respect to distorted probabilities was introduced in Wang et al. (1997) and studied further, e.g. in Bellini and Caperdoni (2007) and Wang (2000).^4^ The first explicit connection of distortion risk measures and insurance pricing was made by introducing the *proportional hazard transform* (Wang 1995, 1996, 1998). Wang et al. (1997) described an axiomatic characterisation of insurance prices as Choquet integrals and Wang (2000) introduces another particular distortion in the general setting of Wang (1996), later called *Wang transform*, with the aim of connecting the pricing of insurance and financial risks. Finally, let us mention that many questions that arise from the practical usage (due to corresponding regulatory frameworks) of the value-at-risk (VaR) and average value-at-risk (AVaR) measures are subsequently studied for a more general class of distortion risk measures, e.g. backtesting methods [see, e.g. Christoffersen and Pelletier (2004) and Ziggel et al. (2014) for VaR, Emmer et al. (2015) and Kratz et al. (2018) for AVaR, and Bettels et al. (2022) for general distortion risk measures and an extensive overview of works on VaR and AVaR backtesting] or risk sharing [see, e.g. Galchion (2010) for VaR, Embrechts et al. (2018) for quantile-based risk measures (range value-at-risk), and Wang (2016), resp. Weber (2018), for more general (resp. *VaR-type*) distortion risk measures].

### Contribution

This paper extends the landscape of previous studies on the combination of risk reduction and risk transfer by bestowing the insurer with a more central role, namely controlling the cost of both risk transfer and risk mitigation. This relates to the real-world situation in cyber insurance, where insurers have started to endow insurance policies (risk transfer) with so-called cyber-assistance services (risk mitigation). We consider a monopolistic, profit-maximising, risk-averse or risk-neutral insurer using a concave distortion risk measure and study separately the cases of cyber-assistance services relating to the concepts of self-protection and self-insurance.^5^ The interaction between the insurer and the insurance buyer(s),^6^ who are risk averse and also use a concave distortion risk measure, is modelled as a Stackelberg game, where the “inner” optimisation problem corresponds to the insurance buyer’s response to a given price structure by the insurer and the “outer” optimisation problem corresponds to the insurer’s problem of determining prices for (cyber) risk transfer and (cyber) assistance services. In particular, we derive the following insights:

- The “The insurer’s problem: single-contract case” section addresses the insurer’s problem in the single-contract case, studying in which cases an insurer is incentivised to encourage risk reduction in her policyholders by sharing the cost of risk reduction measures. We find that under the above assumptions, the insurer would never share the cost of risk reduction in a single-contract, pure self-protection scenario (Theorem 1 and case study in section A.5 in the electronic supplementary information). This does not hold in a single-contract, pure self-insurance scenario, where the optimal share of risk mitigation cost the insurer chooses to bear may depend e.g. on the parameters of the loss size distribution and both parties’ risk aversions (Remark 11 and case study in section A.6 of the electronic supplementary information).
- The “The insurer’s problem: portfolio viewpoint” section extends the insurer’s study of the pure self-protection scenario from a single-contract view to bivariate examples of insurance buyers facing dependent cyber losses under dependence mechanisms relevant for cyber (loss propagation, common events). We demonstrate that the finding from the single-contract case does not carry over, i.e. already for these small toy portfolios, the insurer may have an incentive to subsidise risk mitigation in some policyholders. The study is extended to an example of a larger ($N \ge 2$) portfolio in section A.7.3 in the electronic supplementary information, illustrating the increasing importance of taking a portfolio viewpoint for dependent risks.
- The “Solution to the insurance buyer’s problem” section addresses the insurance buyer’s solution to his problem of choosing an optimal combination of insurance and risk mitigation for a given price structure by the insurer (Corollary 3) and deduces the potentially complementary nature of the two activities (Corollary 4).

In summary, the contribution offers threefold insights, regarding the viewpoints of insurers, (prospective) insurance buyers, and the general (cyber insurance) market. For insurers, the study of the insurer’s bivariate optimisation problem offers a first guidance to the optimal pricing of insurance policies including risk mitigation services (under specific assumptions). For insurance buyers, it is also invaluable to better understand how different contracts would be optimally priced by an insurer, in particular that the price structure a prospective policyholder is offered (and the included incentive for risk reduction) may not only depend on his own characterictics, but on the insurer’s existing portfolio and the (assumed prospective) dependence between losses.^7^ The study of the insurance buyer’s problem on the optimal combination of risk transfer and risk mitigation is not conceptually new, but its detailed consideration offers valuable insights. Next to naturally providing guidance on the recommended course of action for insurance buyers, it may serve to theoretically explain the *insurance gap* observed in the cyber insurance market (see, e.g. Shetty et al. 2018), an offer-demand mismatch caused by the fact that potential buyers often look for insurance against extreme cyber events and tend to perceive asked prices of such coverage as excessive, while insurers seek to limit their liabilities from unprecedented cyber losses either by limiting coverage or by charging heavy risk premiums. One way to mitigate this mismatch, where no premium acceptable to both parties can be found for the original risk, is to equip insurance policies with (potentially subsidised) risk reduction services which help to alter the risk in a way that allows the insurer to reduce premiums and offer desired coverage at an acceptable (from the buyer’s viewpoint) premium.

The remainder of this paper is structured as follows: in the “Model set-up and assumptions” section, the model assumptions and set-up are explained; in the “Solution to the insurance buyer’s problem” and “The insurer’s problem: single-contract case” sections the insurance buyer’s and insurer’s optimisation problems, respectively, are studied in the single-contract setting; the “The insurer’s problem: portfolio viewpoint” section addresses the insurer’s problem in simple portfolio settings with dependent losses. The “Conclusion” section summarises and outlines future research opportunities.

## Model set-up and assumptions

### Risk mitigation services in cyber insurance (cyber assistance)

We first consider a model involving one profit-maximising, risk-averse insurer (‘she’) and one risk-averse (insurance) buyer (‘he’). Before detailing the model set-up and the mechanics of the sequential optimisation game, we give some compelling arguments for considering *risk mitigation services* in conjunction with cyber insurance policies and subsume types of risk mitigation services into three categories:

1. *Reduction of loss probability after initial risk assessment:* Insurers often work with specialised IT service providers (SP) who help them to thoroughly classify a prospective client’s IT security. After the effort of such an assessment is invested, the SP and the assessed company share a common understanding of the company’s IT security standpoint and potential need for action. Given that the risk is deemed insurable, a joint offer by SP and insurer to the company is in everyone’s interest: the company receives insurance protection and high-quality IT security maintenance services as a joint package without the necessity of extra effort to ensure complying with the insurer’s requirements, which is especially relevant for small companies. The insurer does not forfeit the upfront investment for risk assessment and has certainty about the maintenance and potential improvement of the IT security according to the SP’s assessment. The SP has certainty about the company’s willingness to comply with recommendations in order not to jeopardise insurance coverage, and about insurance coverage with a trusted “counterparty” who will not doubt their work in case a cyber event still occurs.^8^
2. *Reduction of loss magnitude in a cyber event:* Among the insured’s obligations within a typical cyber insurance contract is the immediate notification of the insurer in case of a (suspected) cyber event. This allows the insurer to supply immediate technical and legal support in order to mitigate economic losses. Naturally, it is in both the company’s and insurer’s interest for these experts to already have a good understanding of the company’s IT security landscape and to be available immediately, both of which can be guaranteed by including these services – to be performed by a service provider collaborating with the insurer – in an insurance contract.
3. *Use of insurer’s knowledge about current cyber-loss landscape:* While many businesses dedicate their attention to describing current cyber-threat trends, insurers have invaluable knowledge about economic losses currently suffered by their portfolio of clients. Companies are usually obliged by contract to notify their cyber insurer about cyber events, while naturally being reluctant to voluntarily share this information publicly or with external parties (e.g. researchers) in order to avoid reputational damage. Therefore, insurers have an information advantage regarding current threats and their common causes (e.g. a new trend in phishing mails or a vulnerability in a software used by companies of a specific industry sector) and can make use of this extra knowledge to warn other policyholders who are particularly prone to similar threats and vulnerabilities (e.g. all policyholders from the same industry sector or all using some vulnerable software). The benefit of doing so is reducing the probability of additional cyber losses from the same cause in their portfolio. This is especially relevant for large companies with sophisticated IT security (who may already work with external SPs) which might not find it necessary to additionally take advantage of (R1) and (R2) as part of insurance coverage. For the insurer, this type of mitigation helps to reduce the impact of systemic events and, thus, accumulation risk in the portfolio.

#### Remark 1

(Link between theoretical and marketed types of risk reduction service) The types of service currently offered on the cyber insurance market and suggested above direct quite naturally to the concepts of self-protection and self-insurance:

1. Describes *pre-incident services* which are *self-protection activities*. Examples are network security, back-up of critical systems and data, anti-malware tools, identity and access management, IT security consulting, employee awareness measures, patch management, and mobile device management (Munich Re 2021).
2. Describes *post-incident services* which are *self-insurance activities*, such as restoration of data, 24h help hotlines, forensic post-breach services, legal advice, and consulting in case of extortion (Munich Re 2021).
3. Describes a type of *self-protection activity* not yet advertised on the market, as contracts are typically viewed stand alone. However, using the insurer’s portfolio knowledge to install such warning mechanisms would be an important way to use dependencies (and information) between risks to the insurer’s and insureds’ advantage.

Of course, the above categorisation simplifies reality regarding several points: pre- and post-incident services are usually not offered disjointly, but as a complete “cyber assistance” service package, and each service activity within the above categories can have beneficial effects on both cyber-loss probability and severity. For example, anti-malware tools not only serve their primary purpose, i.e. to deter malware from entering the system (preventing a cyber incident completely), but as a side effect – in case malware circumvents the protection – may help to identify the source of a cyber incident more efficiently and reduce the time until system functionality is restored (reducing the economic impact of an occurred cyber incident). Nevertheless, from a mathematical viewpoint, it is convenient (and in line with previous academic work) to study the two concepts separately and therefore it is helpful to keep in mind the types of “real-world cyber assistance activities” they relate to.^9^ One aspect of cyber assistance which is purposely omitted here is risk-assessment services (see section A.1 in the electronic supplementary information). This includes, e.g. extensive IT audits conducted by an IT service provider collaborating with the insurer to analyse a company’s IT security provisions, to identify vulnerabilities, and to provide recommended courses of action.

### Model prerequisites

Following the framework of Bensalem et al. (2020), we assume that over a given policy year, the buyer faces a random loss represented by a non-negative random variable (r.v.) *X* from a family of distributions $F_s$ indexed by a parameter $s \in [0,\infty )$.^10^ For $X \sim F_s$, we denote the corresponding survival function by $\overline{F}_{X,s}(x) = \mathbb {P}_s(X > x),\; x \in \mathbb {R}$, and its generalised inverse, the tail quantile function, by $\overline{q}_{X,s}(u) = \overline{F}_{X,s}^{-1}(u) = \inf \{x \in \mathbb {R}: \overline{F}_{X,s}(x) \le u\},\; u \in (0,1)$. To formalise the relationship between the parameter *s* and the distributions $F_s$, we assume a decreasing order in the sense of first-order stochastic dominance ($\le _{FSD}$), i.e. for any $0 \le s_1< s_2 < \infty$ and $X_1 \sim F_{s_1},\; X_2 \sim F_{s_2}$ it holds that $X_2 \le _{FSD} X_1$. This is equivalent (see Müller and Stoyan (2002), Theorem 1.2.8) to assuming

$$\begin{aligned} 0\le s_1< s_2 < \infty \implies \mathbb {E}_{s_2}\big [f(X)\big ] \le \mathbb {E}_{s_1}\big [f(X)\big ] \end{aligned}$$

for any non-decreasing^11^ function $f: \mathbb {R}\rightarrow \mathbb {R}$ for which both expectations exist. We furthermore assume that $\mathbb {E}_s[X] > 0,\; \forall s \in [0,\infty )$, meaning that no risk reduction can ever completely eliminate the possibility of a positive loss.

The decreasing order in the sense of FSD of $F_s$ implies that

A1 $$\begin{aligned} \text {for any}\;\; u \in (0,1),\;\;\text {the map}\;\; s \mapsto \overline{q}_{X,s}(u)\;\; \text {is non-increasing.} \end{aligned}$$

This means that increasing *s* alters the risk *X* in such a way that for any probability level, the minimum loss amount that is exceeded by *X* with this probability does not increase.

#### Assumption 1

(Convexity of tail quantile in *s*). Furthermore, we assume that

A2 $$\begin{aligned} \text {for any}\;\; u \in (0,1),\;\;\text {the map}\;\; s \mapsto \overline{q}_{X,s}(u)\;\; \text {is convex.} \end{aligned}$$

This assumption can be interpreted as a decrease in marginal effect of service, i.e. the impact per unit of *s* on the risk *X* in the sense of (A1) does not increase as the baseline level of *s* increases, which is a very natural economic assumption.

We assume that both parties evaluate risk by using law-invariant, coherent risk measures, whose properties are recalled in section A.2 of the electronic supplementary information. An important class of risk measures are so-called distortion risk measures (see Wang et al. 1997), defined for a real-valued r.v. *X* as the usual Choquet integral that simplifies for non-neg. *X* to

1 $$\begin{aligned} \rho (X) := \int _{0}^{\infty } \psi (\overline{F}_X(x)) \mathrm {d}x \overset{e.g.\, [32]}{=} \int _{0}^{1} \overline{q}_X(u) \mathrm {d}\psi (u), \end{aligned}$$

where $\psi : [0,1] \rightarrow [0,1]$ is a distortion function^12^ and $\overline{q}_X(u),\; u \in (0,1),$ is the tail quantile function. From Eq. (1), one can directly see that the distortion risk measure for a.s. non-neg. losses represents a distorted expectation of *X*.

#### Assumption 2

(Concavity of distortion function). Concavity of the distortion function is a natural economic assumption. As it corresponds to assigning a higher weight to small probability events, it describes risk aversion of the decision maker, a standard assumption and indeed a prerequisite for the existence of insurance. Therefore, we will restrict our analysis to distortion risk measures with concave distortion, a class of coherent, law-invariant risk measures.^13^

#### Remark 2

[Distortion risk measures and stochastic dominance, e.g. Dhaene et al. (2006)] Any distortion risk measure $\rho$ preserves first-order stochastic dominance, i.e. for any a.s. non-negative r.v. $X_1, X_2$, it holds that $X_1 \le _{FSD} X_2 \implies \rho (X_1) \le \rho (X_2)$.

#### Example 1

Table 1 lists some commonly used distortion risk measures and their corresponding distortion functions. In the case studies of our latter analysis, we focus on the *proportional hazard transform*.

**Table 1:** Popular distortion risk measures (DRM) and underlying distortion functions

Risk measure	Distortion $\psi (u)$, $u \in (0,1)$	$\psi$ concave	Parameters and remarks
$VaR_\alpha$	$\mathbf {1}_{\{u > 1-\alpha \}}$	No	$\alpha \in (0,1)$
$AVaR_\alpha$	$\min \big \{\frac{u}{1-\alpha };1\big \}$	Yes	$\alpha \in (0,1)$
Wang transform RM (Wang 2000)	$\Phi \Big (\Phi ^{-1}(u) + \lambda \Big )$	Yes	$\lambda \in (0,\infty )$, $\Phi$ is std. Normal c.d.f.
Beta DRM (Wirch and Hardy 2000)	$\frac{1}{\beta (a,b)} \int _{0}^{u} t^{a-1}(1-t)^{b-1}\mathrm {d}t$	Yes	$0 < a \le 1,\; b \ge 1$, $\beta (a,b) = \frac{\Gamma (a)\Gamma (b)}{\Gamma (a+b)}$
Proportional Hazard (PH) transform RM (Wang 1995)	$u^r$	Yes	$r \in (0,1],$ Special case of Beta DRM

The above assumptions on the risk measures and loss distributions [in particular (A2)] are convenient insofar as they imply that the map $s \mapsto \rho _s(X)$ (and as a special case $s \mapsto \mathbb {E}_s[X]$) is convex, continuous, non-increasing, and $\rho _s(X) \ge \mathbb {E}_s[X] > 0$ [see Bensalem et al. (2020) and section A.3 in the electronic supplementary information].

### Interaction between cyber-insurance buyer and insurer

We now describe how the interaction between insurance buyer and insurer in the case of a cyber insurance contract is modelled as a *Stackelberg game*, i.e. a sequential optimisation game between two parties, where one party (the *leader*) moves first by choosing her strategy and the other party (the *follower*) moves second by choosing his strategy depending on the selected strategy of the leader, whereby both parties seek to maximise a gain or utility function or equivalently, minimise a loss function. For a general introduction to Stackelberg games, see Fudenberg and Tirole (1991) and Osborne and Rubinstein (1994). A common tool to solve a Stackelberg game is *backward induction* (see Fudenberg and Tirole 1991), i.e. first solving the follower’s problem for any possible choice of the leader’s strategy and then – knowing all the follower’s responses – solving the leader’s problem. The search for a solution (and its existence) therefore depends on the specific formulation of both problems, which we now detail in our case.

0. Common (correct) knowledge of initial loss distribution

The prospective insurance buyer approaches the insurer to inquire about offered prices for cyber insurance policies (in person or by entering data into an online calculation system), where in order to receive price quotes, he needs to provide information that allows the insurer (with the help of an IT service provider) to classify his risk profile given his characteristics (e.g. industry sector, company size, IT security measures). We assume he provides the information truthfully and to the best of his knowledge, such that buyer and insurer have a common, unambiguous view of the original loss distribution, denoted $F_0$.^14^ The real-world uncertainty of either parties’ knowledge of the unknown initial loss distribution is not studied here. Naturally, the question of accurate cyber-risk assessment has gained increased practical importance and expresses itself, e.g. in the increasing number of service providers in this domain, see, e.g. Bosch CyberCompare as an example or Advisen for a market overview. For a seminal discussion of cyber-risk assessment services and a proposal how to approach them mathematically, see section A.1 in the electronic supplementary information.

1. Prices quotes by the insurer

Given the buyer’s original risk $X \sim F_0$, the insurer offers price quotes $\Pi$ for a range of contracts, where each offered contract is characterised by the included level of *risk mitigation service* $s \in [0,\infty )$.^15^ Assume that the price of entering a contract with service level $s \in [0,\infty )$ is given by

$$\begin{aligned} \Pi (s)&= (1+\theta )\mathbb {E}_s[X] + \beta c(s), \end{aligned}$$

where the first term represents the *risk premium* according to the expected value principle with loading $\theta$ and the second term denotes the *service premium*, where we assume that providing service at level $s \in [0,\infty )$ requires a monetary cost of *c*(*s*) for the insurer, of which a proportion $\beta \in [\underline{\beta },1]$, $\underline{\beta } > 0$,^16^ is charged to the insured and, thus, the remaining proportion $(1-\beta )$ can be regarded a subsidy by the insurer to incentivise risk reduction. Analogously to (A1) and (A2), $s \mapsto c(s)$ is assumed to be increasing, strictly convex, and continuous with $c(0)=0$ and $\displaystyle \lim _{s \rightarrow \infty } c(s) = \infty$. The cost incurred by the insurer can be understood e.g. as the internal cost charged by the IT service provider for providing pre- or post-incident services (i.e. (R1) and (R2)) or the administrative cost of monitoring and evaluating loss data to warn policyholders about imminent threats (i.e. (R3)). Thus, the insurer’s task is to choose a combination $(\theta ,\beta ) \in [0,\infty ) \times [\underline{\beta },1]$ which then defines price quotes for all feasible contracts.

*2. Choice of a contract by the buyer (or opt-out)*

Given a family of prices $\Pi (s)$ for all feasible contracts, the buyer selects a contract by choosing a proportional insurance share $\alpha \in \{0,1\}$ (to opt into full insurance $\alpha = 1$ or to not buy insurance $\alpha = 0$) and the amount of risk mitigation service $s \in [0,\infty )$. We assume that the purchase of (additional) service at any level *s* is also feasible outside of an insurance contract, but at a higher cost $\beta _o c(s)$ with $\beta _o > 1$. This can be understood as the cost of buying service directly through an IT service provider (without a discount offered for insurance customers) or from the insurer herself at a mark-up.^17^ In summary, given the prices for all feasible contracts as offered by the insurer, the insurance buyer’s problem consists of choosing $(\alpha ,s) \in \{0,1\} \times [0,\infty )$. We detail in Remark 4 how the insurance buyer’s choice encapsulates three classical ways of dealing with risk (acceptance, reduction, transfer), see, e.g. Marotta et al. (2017).

*3. Solution by backward induction*

To find both parties’ optimal solution, we use backward induction (see, e.g. Osborne and Rubinstein 1994) by first finding the buyer’s optimal response $(\alpha ^*,s^*)$ to any insurer’s choice of $(\theta ,\beta )$ and second, given all optimal buyer’s responses, finding the insurer’s optimal choice $(\theta ^*(\alpha ^*,s^*),\beta ^*(\alpha ^*,s^*))$. In order to formulate and solve the game, below we state the loss functions of buyer and insurer, respectively.

#### Remark 3

We highlight some similarities and distinctions between the present work and the study of Bensalem et al. (2020), whose framework was our inspiration: as indicated above, the choice of risk measures and the ordering of loss distributions follows Bensalem et al. (2020) and from the insurance buyer’s point of view, the risk reduction service *s* fulfils a very similar role to the *effort* considered in Bensalem et al. (2020), yielding related optimisation problems for the buyer within the Stackelberg game. In the present study, however, the insurer’s role is more central, as she controls the cost of risk mitigation service within an insurance contract (via the share $\beta$ of administrative cost charged to the insured). This implies that the insurer has to solve a two-dimensional problem (choosing a combination of *risk premium* and *service premium* optimally), and circumvents the moral-hazard problem that often occurs in studies on prevention and insurance. As in the present setting the risk mitigation service is offered through the insurer, the challenge of ensuring that the buyer actually complies with the agreed-upon optimal level of risk reduction (according to which insurance is priced) does not arise. Furthermore, we extend the study of the interaction with one insurance buyer to toy examples of interactions with a portfolio of dependent buyers, a particularly relevant issue in the cyber context.

### Formalisation of the Stackelberg game

We now combine the assumptions of the above sections to formulate the optimisation problems of both parties within a Stackelberg game. For the reader’s convenience, all parameters and functions appearing within the optimisation problems are summarized in Tables 2 and 3. The insurance buyer’s objective is to minimise a coherent and law-invariant risk measure $\rho _1$ associated to his total position including insurance, while the insurer’s objective is to minimise, given the buyer’s optimal response, another coherent and law-invariant risk measure $\rho _0$ associated to her (negative) total loss.

BP $$\begin{aligned} \min _{(\alpha ,s) \in \{0,1\} \times [0,\infty )} L_1(\alpha ,s)&:= \rho _{1,s}(X) + \beta _o c(s) + \alpha \Big [(1+\theta )\mathbb {E}_s[X] - \rho _{1,s}(X) + (\beta - \beta _o) c(s) \Big ], \end{aligned}$$

IP $$\begin{aligned} \min _{(\theta ,\beta ) \in [0,\infty ) \times [\underline{\beta },1]} L_0(\theta ,\beta )&:= \alpha ^*(\theta ,\beta )\Big (\rho _{0,s^*(\theta ,\beta )}(X) - (1+\theta )\mathbb {E}_{s^*(\theta ,\beta )}[X] + (1-\beta ) c(s^*(\theta ,\beta ))\Big ), \end{aligned}$$

where we have used that both risk measures are cash-additive and positively homogeneous. It is obvious that the insurer’s loss depends on $(\theta ,\beta )$ directly as well as via the buyer’s optimal response denoted $(\alpha ^*(\theta ,\beta ),s^*(\theta ,\beta ))$.

#### Remark 4

(Interpretation of insurance buyer’s choice) The buyer’s options correspond to three classical ways of dealing with risk:

- **Risk acceptance:** The choice $(\alpha ,s)=(0,0)$ yields $L_1(0,0) = \rho _{1,0}(X)$, i.e. is equivalent to opting out of buying insurance or services and just retaining and accepting the original risk.
- **Pure risk transfer:** Choosing $(\alpha ,s) = (1,0)$ yields $L_1(1,0) = (1+\theta )\mathbb {E}_0[X]$, meaning that the buyer opts for fully insuring the original risk.
- **Pure risk reduction:** A choice $\alpha =0,\;s>0$ yields $L_1(0,s) = \rho _{1,s}(X) + \beta _o c(s)$, i.e. the buyer opts out of risk transfer but chooses to reduce the original retained risk by purchasing risk reduction services (from the insurer outside of a policy or from a service provider directly).^18^
- **Combination of risk transfer and risk reduction:** A choice $\alpha =1,\;s>0$ yields $L_1(1,s) = (1+\theta )\mathbb {E}_s[X] + \beta c(s)$ and means that the buyer chooses an insurance policy with risk mitigation services included, i.e. opts for insuring a reduced risk.

#### Remark 5

(Buyer’s and insurer’s optimal attainable loss)

- Note that as the insurance buyer starts out by facing the non-negative random loss *X*, by assumption $L_1(\alpha ^*,s^*) > 0$, i.e. the insurance buyer can never completely eliminate his risk or even make a profit.
- On the contrary, we naturally assume that the insurer only offers a contract if it is profitable, i.e. only if she can obtain a negative loss $L_0(\theta ^*,\beta ^*) < 0$. Otherwise, she would refrain from offering a contract by refusing to quote a price.

## Solution to the insurance buyer’s problem

As the analysis of (BP) is an extension of Bensalem et al. (2020), this section focuses on the additions to their analysis originating from the new formulation of (IP) and the interpretation of all results in the cyber insurance context. Derivations and proofs are outlined in section A.3 of the electronic supplementary information. First, one determines the set of values of *s* such that full insurance is demanded (i.e. $\alpha ^*(s) = 1$, denoted $\mathcal {I}$) and its complement (no insurance is demanded, $\alpha ^*(s) = 0,\; \mathcal {N}:= \mathcal {I}^{\mathsf {c}}$). Note that for fixed *s*, the choice $\alpha ^* \in \{0,1\}$ depends only on the sign of the expression in the last bracket of (BP) such that it follows:

2 $$\begin{aligned} \alpha ^* = 1&\iff G^\beta (s) := \frac{\rho _{1,s}(X)}{\mathbb {E}_s[X]} + (\beta _o-\beta )\frac{c(s)}{\mathbb {E}_s[X]} \ge (1+\theta )&\implies \mathcal {I}:= \{s \in [0,\infty ): G^\beta (s) \ge (1+\theta )\}, \end{aligned}$$

3 $$\begin{aligned} \alpha ^* = 0&\iff G^\beta (s)< (1+\theta )&\implies \mathcal {N}:= \{s \in [0,\infty ): G^\beta (s) < (1+\theta )\}. \end{aligned}$$

On the sets $\mathcal {I}$ and $\mathcal {N}$, the buyer’s loss function is a sum of convex functions:

$$\begin{aligned} L_{1,\mathcal {N}}(s)&:= \rho _{1,s}(X) + \beta _o c(s), \; s \in \mathcal {N},\\ L_{1,\mathcal {I}}^{\theta ,\beta }(s)&:= (1+\theta )\mathbb {E}_s[X] + \beta c(s), \; s \in \mathcal {I}. \end{aligned}$$

Therefore, one considers (BP) separately on $\mathcal {I}$ and $\mathcal {N}$ and compares the resulting local minima to obtain a global minimum. To this end, one first needs to study $\mathcal {I}$ and $\mathcal {N}$ for given $(\theta ,\beta )$, i.e. the behaviour of $s \mapsto G^\beta (s)$ with respect to the threshold $(1+\theta )$. We know that by assumption and Lemma 1 (see section A.3 in the electronic supplementary information), $s \mapsto G^\beta (s)$ is continuous and its second summand $s \mapsto (\beta _o - \beta ) \frac{c(s)}{\mathbb {E}_s[X]}$ is non-negative and increasing.^19^ In this study, we consider two cases:

- **Self-protection:** In a self-protection scenario (Ehrlich and Becker 1972), i.e. if service only affects the probability of a loss, the map $s \mapsto \frac{\rho _{s}(X)}{\mathbb {E}_s[X]}$ is monotone non-decreasing (see Bensalem et al. 2020, Lemma 3.2, and section A.3 in the electronic supplementary information). Economically, this means that increased risk reduction has a larger impact on (reducing) the price of insurance than on (reducing) the risk.^20^ Mathematically, this implies increasingness of the entire map $s \mapsto G^\beta (s)$, meaning that $G^\beta (s)$ could intersect (for given $\beta$ and $\theta$) the threshold $(1+\theta )$ at most once, making $\mathcal {I}$ and $\mathcal {N}$ straightforward to determine. This setting will be considered in the following.
- **Special case of self-insurance:** Bensalem et al. (2020) argue that in a scenario of self-insurance, i.e. in the present context if service only affects the severity of a cyber loss, for some standard loss distributions (e.g. Pareto, Weibull, or Log-Normal), $s \mapsto \frac{\rho _{s}(X)}{\mathbb {E}_s[X]}$ is monotone non-increasing. This does not lead to a straightforward expression of $\mathcal {I}$ and $\mathcal {N}$, as monotonicity of $s \mapsto G^\beta (s)$ is not implied and there is a priori no limit for the number of times it crosses a given threshold $(1+\theta )$ for $s \in [0,\infty )$, such that no general results for this case can be stated. In section A.6 in the electronic supplementary information, we study the particular case of a Pareto-distributed loss whose severity is affected by risk reduction service. Here, under mild assumptions, $G^\beta (s)$ turns out to be strictly convex (with $\displaystyle \lim _{s \rightarrow \infty } G^\beta (s) = \infty$), yielding only one additional case compared to the self-protection case, namely $G^\beta (s)$ intersecting the level $(1+\theta )$ exactly twice.

As outlined above, we now consider a scenario of *self-protection* (Ehrlich and Becker 1972), i.e. an a.s. non-negative loss *X* which stems from a family of zero-inflated distributions of the form

4 $$\begin{aligned} F_{X,s}(x) = [(1-p(s)) + p(s) F_Y(x)]\mathbbm {1}_{\{x \ge 0\}}, \end{aligned}$$

where $s \mapsto p(s) \in [0,1]$ is decreasing and $F_Y$ is the c.d.f. of an a.s. positive r.v. *Y*. This means that a positive loss with c.d.f. $F_Y$ (which could describe a single loss or be a compound distribution describing a cumulative loss) occurs with a probability that can be lowered by purchasing services while the severity distribution remains untouched, relating to (R1) and (R3) above. Ansatz (4) only assumes $s \mapsto p(s)$ to be decreasing (which is natural, as increased service should decrease the loss probability). As a standard economic assumption (e.g. Courbage et al. 2013) is $s \mapsto p(s)$ being convex (decreasing marginal impact), (A2) is not necessarily implied. Therefore, we assume another sufficient condition to ensure convexity of $s \mapsto \rho _s(X)$ for distributions of the form (4), namely that both the *objective* loss probabilities *p*(*s*) and the *subjective* loss probabilities $\psi (p(s))$ are decreasing in a convex way (see Bensalem et al. 2020, Lemma 3.3, and section A.3 in the electronic supplementary information).

### Example 2

As $\psi$ is concave, $s \mapsto p(s)$ must be “sufficiently” convex for the concatenation to be convex; e.g. for the common choice of distortion function $\psi (u) = u^r, r \in (0,1]$, a sufficient condition for the convexity of $\psi (p(s)) = p(s)^r$ would be for $s \mapsto p(s)$ to be *logarithmically convex* (see section A.5 in the electronic supplementary information).

Increasingness of $s \mapsto G^{\beta }(s)$ for any $\beta \in [\underline{\beta },1]$ in the self-protection case allows a convenient expression of the sets $\mathcal {I}$ and $\mathcal {N}$.

### Corollary 1

(Structure of $\mathcal {I}$ and $\mathcal {N}$ in the self-protection case, extension of Bensalem et al. (2020), Lemma 3.2) There exists a constant $\theta _0 \ge 0$ such that:

1. If $\theta \le \theta _0$, then $\mathcal {N}= \emptyset$ and $\mathcal {I}= [0,\infty )$.
2. If $\theta > \theta _0$, then for any $\beta \in [\underline{\beta },1]$, there exists $s_B(\theta ,\beta ) > 0$ such that $\mathcal {N}= [0,s_B(\theta ,\beta ))$ and $\mathcal {I}= [s_B(\theta ,\beta ),\infty )$.

In the latter case, both maps $\theta \mapsto s_B(\theta ,\beta )$ and $\beta \mapsto s_B(\theta ,\beta )$ are increasing.

### Remark 6

**Fig. 1:**
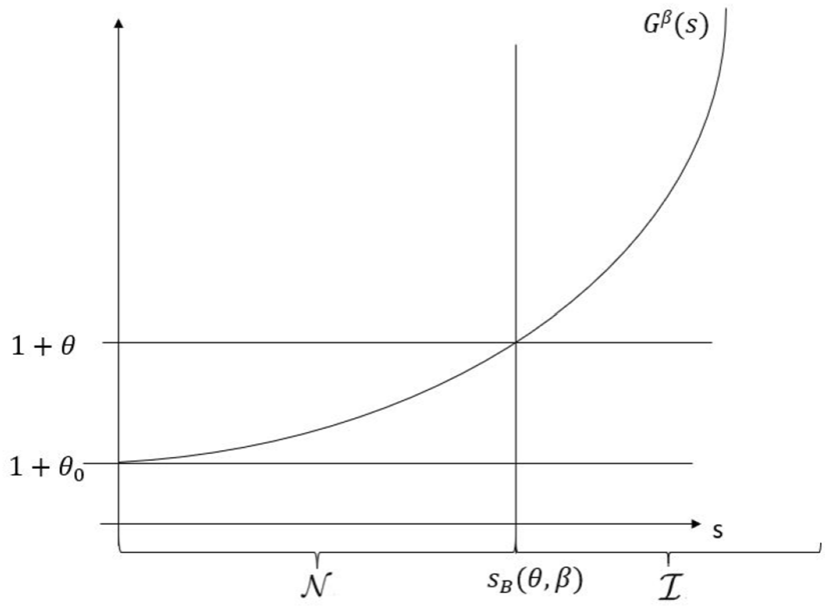
Schematic illustration of $G^\beta (s)$ and resulting $s_B(\theta ,\beta )$ for one value of $\theta$. $\theta _0$ is the minimum value of the loading such that $G^\beta (s)$ intersects the level $1+\theta$, resulting in $\mathcal {N}$ being non-empty

(Interpretation of Corollary 1) Case (1) states that if the loading is lower than a given constant level $\theta _0$, the buyer would purchase insurance already for the original risk (at $s=0$) and therefore at any level *s* (recall that increasing *s* reduces the price more than the risk). Case (2), illustrated in Figure 1, corresponds to a situation where the loading is too high for the buyer to insure the original risk, but by adding a service level of at least $s_B(\theta ,\beta )$ (which depends on $\theta$ as well as its relative cost $\beta$), an insurance contract with loading $\theta$ becomes acceptable for the buyer.

This directly relates to the *insurance gap* on the cyber insurance market: for the pure risk transfer ($s=0$) policies offered with loading $\theta$, it may not be acceptable for the buyer to insure the original risk at the price the insurer demands. To make an insurance contract possible, either $\theta$ would have to be lowered to at most a level $\theta _0$ (move from case (2) to case (1)) or risk reduction services equivalent to a level $s_B$ would have to be offered as part of the policy (in case (2), enable a move from $\mathcal {N}$ to $\mathcal {I}$).

Lastly, it is intuitive that if the *risk premium* or *service premium* increase, the with-insurance solution becomes relatively more expensive for the buyer, and the interval corresponding to $\mathcal {N}$ (resp. $\mathcal {I}$) becomes larger (resp. smaller).

To solve the buyer’s problem, first note that $L_{1,\mathcal {N}}(s)$, resp. $L_{1,\mathcal {I}}^{\theta ,\beta }(s)$, each admit a unique global minimiser on $[0,\infty )$, denoted $s_N$ resp. $s_I(\theta ,\beta )$.

### Corollary 2

(Solutions of separate problems, extension of Bensalem et al. (2020, section 3.3)

1. For any $\beta \in [\underline{\beta },1]$, there exists a positive constant $\theta _N(\beta ) > \theta _0$ such that

$$\begin{aligned} \theta < \theta _N(\beta )&\implies argmin_\mathcal {N}L_{1,\mathcal {N}}(s) = s_B(\theta ,\beta ),\\ \theta \ge \theta _N(\beta )&\implies argmin_\mathcal {N}L_{1,\mathcal {N}}(s) = s_N. \end{aligned}$$

The map $\beta \mapsto \theta _N(\beta )$ is decreasing.

2. For any $\beta \in [\underline{\beta },1]$, there exists a constant $\theta _I(\beta )$ such that

$$\begin{aligned} \theta \le \theta _I(\beta )&\implies s_I(\theta ,\beta ) = 0,\\ \theta> \theta _I(\beta )&\implies s_I(\theta ,\beta ) > 0. \end{aligned}$$

In the latter case, the following hold:

(i) For any $\beta \in [\underline{\beta },1]$, the map $\theta \mapsto s_I(\theta ,\beta )$ is increasing.
(ii) For any $\theta > 0$, the map $\beta \mapsto s_I(\theta ,\beta )$ is decreasing.

### Remark 7

(Interpretation of Corollary 2) 

Part 1.: As the loading $\theta$ increases, the set $\mathcal {N}$ (no insurance) expands, i.e. the boundary $s_B(\theta ,\beta )$ increases (shift to the right in Fig. 1). The value $\theta _N(\beta )$ is the smallest loading such that the global minimiser of $L_{1,\mathcal {N}}(s)$ lies in $\mathcal {N}$

Part 2.: For fixed service cost $\beta$, as $\theta$ increases, it becomes relatively more expensive to transfer risk, which makes it economically rational to reduce the to-be-insured risk by increasing service. Vice versa, for fixed risk loading $\theta$, as $\beta$ increases, and thus, service becomes relatively more expensive, it is economically rational to decrease the purchased amount of service.

Corollary 2 does not make a statement about the local solution on $\mathcal {I}$. As both $s_I(\theta ,\beta )$ (by Corollary 2) and $s_B(\theta ,\beta )$ (by Corollary 1) are non-decreasing in $\theta$, to determine the local solution on $\mathcal {I}$ and the global solution to the minimisation of $L_1(\alpha ^*(s),s)$, one has to consider all possible cases regarding the order of $s_N, s_I(\theta ,\beta ), s_B(\theta ,\beta )$ (see sectin A.3 in the electronic supplementary information).

### Corollary 3

(Global solution in the self-protection case, extension of Bensalem et al. (2020, Theorem 3.2) For any $\beta \in [\underline{\beta },1]$, there exists a constant $\theta _R(\beta ) \ge 0$, such that:

(i) If $\theta \le \theta _R(\beta )$, the global minimiser of $L_1(\alpha ^*(s),s)$ is $(\alpha ^*,s^*) = (1,s_I(\theta ,\beta ))$.
(ii) If $\theta > \theta _R(\beta )$, the global minimiser of $L_1(\alpha ^*(s),s)$ is $(\alpha ^*,s^*) = (0,s_N)$.

Furthermore, it holds $\theta _R(\beta ) \ge \theta _N(\beta )$ and the map $\beta \mapsto \theta _R(\beta )$ is non-increasing.

### Remark 8

(Interpretation of Corollary 3) For any choice of $\beta$, there is a maximum loading $\theta _R(\beta )$ the insurance buyer is willing to accept: if it is not exceeded, he subscribes to full insurance with service level $s_I(\theta ,\beta )$; else, he refrains from purchasing insurance and buys service at level $s_N$ from an outside provider. The maximum acceptable loading decreases as the share of service cost increases, which is intuitive as the buyer accepts the contract if his total loss with insurance does not exceed his (fixed) total loss without insurance.

The relationship between risk loading and service demand is summarised in Corollary 4.

### Corollary 4

(Based on Bensalem et al. 2020, Corollary 3.3) For any $\beta \in [\underline{\beta },1]$, the map $\theta \mapsto s^*(\theta ,\beta )$ is non-decreasing for $\theta \le \theta _R(\beta )$ and constant (equal to $s_N$) for $\theta > \theta _R(\beta )$. It has a negative jump of size $s_N - s_I(\theta _R(\beta ),\beta )$ at $\theta = \theta _R(\beta )$, which means that demand for risk transfer and service can be complements.

### Remark 9

(Interpretation of Corollary 4 in the cyber context) Corollary 4 is meaningful in cyber insurance: earlier game-theoretic studies concerned with the existence and efficiency of a cyber-insurance market where agents in a network invest in interdependent security measures (e.g. Lelarge and Bolot 2009; Schwartz et al. 2013; Schwartz and Sastry 2014; Shetty et al. 2010, 2010) have in many cases concluded that given the availability of cyber insurance, individuals’ willingness to invest in self-protection decreases and it is, thus, generally not possible to design insurance as a means to reach socially optimal levels of investment. Corollary 4 emphasises the much more optimistic perspective that in case of self-protection, the existence of insurance can indeed lead to higher optimal levels of risk reduction at least for individual policyholders. While we do not consider negative externalities of interdependent security investments, it is reasonable to postulate that by subscribing to insurance with a high service level, policyholders inadvertently benefit other agents in their network, e.g. by reducing the risk of cyberattacks being propagated through their systems or by providing loss data the insurer can use to warn other policyholders.

Furthermore, Corollary 4 allows another understanding of the cyber insurance gap: as the optimal service demand within insurance can be higher than without insurance, for a given combination $(\theta ,\beta )$ that an insurer demands in practice, if the service that can be offered is limited (e.g. due to technical constraints or due to limited contracts between insurers and service providers), the optimal within-insurance service level may not be attainable and the company may prefer the no-insurance solution. A way to close (or narrow) the gap would be to either decrease the premium or to increase the amount of available service within an insurance policy to make $s_I(\theta ,\beta )$ attainable.

Having found the insurance buyer’s optimal response to any combination $(\theta ,\beta ,\beta _o)$, we address the insurer’s problem of choosing $(\theta ,\beta )$ to minimise her loss over all optimal responses of the buyer.

## The insurer’s problem: single-contract case

Given the results of Corollary 3, (IP) reduces to a minimisation over a compact set:

5 $$\begin{aligned} \min _{(\theta ,\beta ) \in \mathcal {A}:= [0,\theta _R(\beta )] \times [\underline{\beta },1]} L_0(\theta ,\beta ) = \rho _{0,s_I(\theta ,\beta )}(X) - (1+\theta )\mathbb {E}_{s_I(\theta ,\beta )}[X] + (1-\beta ) c(s_I(\theta ,\beta )), \end{aligned}$$

assuming that the obtainable objective value of (5) is negative. This corresponds to a choice $(\theta ,\beta )$ yielding full risk transfer with service level $s_I(\theta ,\beta ) \ge 0$ as the buyer’s optimal response. In case the insurer could not obtain a negative objective value in (5), she abstains from offering risk transfer by choosing $\theta > \theta _R(\beta )$ in (IP). In this case, the buyer’s optimal response is $(\alpha ^*,s^*) = (0,s_N(\beta _o))$, i.e. to buy service at level $s_N(\beta _o)$ outside an insurance policy.^21^ Note that the special case $\beta = 1$, where the insurance buyer carries the full cost of self-protection, has already been studied previously, the difference here being that the self-protection measures can be obtained cheaper within an insurance contract, increasing the maximum risk premium chargeable by the insurer.

We now state that in the self-protection case, choosing $\beta = 1$ is also a solution to the more general problem (5). The steps leading to this result are outlined subsequently, proofs are postponed to section A.4 in the electronic supplementary information.

### Theorem 1

(Solution of (5) in the self-protection case) Let the assumptions of Lemma 2 (self-protection, see section A.3 in the electronic supplementary information) hold. Then, a solution $(\theta ^*,\beta ^*)$ to the minimisation problem (5) lies in the compact set $\{(\theta ,1):\theta \in [0,\theta _R(1)] \}$. This means that in the self-protection case, i.e. if service only affects the loss probability, it is always optimal for the insurer to shift the full service cost to the insured.

### Example 3

(Zero-inflated Pareto loss) The solution to (5) cannot be characterised further without more structure. Details for the special case of a zero-inflated Pareto-distributed loss are given in section A.5 of the electronic supplementary information. In this case, the insurer’s loss can be shown to be monotone in $\theta$ for $\beta =1$, yielding the solution $\theta ^* = \theta _R(1)$ (see Bensalem et al. 2020). Combining this with Theorem 1 means that for a Pareto-distributed loss whose occurrence probability can be lowered by risk reduction services, an optimal solution for the insurer is given by shifting the full cost of service to the insured and charging the maximum acceptable loading, i.e. $(\theta ^*,\beta ^*) = (\theta _R(1),1)$.

### Remark 10

Theorem 1 does not make a statement about uniqueness of the solution, as uniqueness only holds whenever the maximum attainable loading $\theta _R(\beta )$ is larger than the minimum loading $\theta _I(\beta )$ that makes pure risk transfer undesirable to the insured compared to a combination of risk reduction and risk transfer (i.e. leads to a solution $s_I(\theta ,\beta ) > 0$, see the proof of Corollary 2). This holds true under quite general assumptions on the function $s \mapsto c(s)$, e.g. for its right-side derivative at 0 to vanish, i.e. $c'(s)\vert _{s=0^+} = 0$.

We use the (implicit) definition of the maximum feasible loading for any share of service cost $\theta _R(\beta )$ from the proof of Corollary 3, given as

$$\begin{aligned} \theta _R(\beta ) := \sup \big \{\theta \ge 0: L_{1,\mathcal {I}}^{\theta ,\beta }(s_I(\theta ,\beta )) \le L_{1,\mathcal {N}}(s_N)\big \}, \end{aligned}$$

which is well-defined for any $\beta \in [\underline{\beta },1]$, as the map $\theta \mapsto L_{1,\mathcal {I}}^{\theta ,\beta }(s_I(\theta ,\beta ))$ is increasing with $L_{1,\mathcal {I}}^{0,\beta }(s_I(0,\beta )) < L_{1,\mathcal {N}}(s_N)$. Furthermore, it is shown that for any $\theta \ge 0$ (resp. $\theta > \theta _I(\beta )$), the map $\beta \mapsto L_{1,\mathcal {I}}^{\theta ,\beta }(s_I)$ is non-decreasing (increasing) such that $\beta \mapsto \theta _R(\beta )$ is non-increasing (decreasing). By denoting $\underline{\theta } := \theta _R(1)$ and $\bar{\theta } := \theta _R(\underline{\beta })$, it holds $L_\mathcal {I}^{\theta ,\underline{\beta }}(s_I(\theta ,\underline{\beta })) < L_\mathcal {N}(s_N)$ for any $\theta \in [0,\bar{\theta }]$, such that one can likewise define for any such $\theta$ the constant

$$\begin{aligned} \beta _M(\theta ) := \max \big \{\beta \in [\underline{\beta },1]: L_\mathcal {I}^{\theta ,\beta }(s_I(\theta ,\beta )) \le L_\mathcal {N}(s_N)\big \}, \end{aligned}$$

denoting the maximum feasible share of service cost such that the contract is accepted for a given loading. The map $\theta \mapsto \beta _M(\theta )$ is by definition non-increasing on $\theta \in [0,\bar{\theta }]$. As a corollary of Lemma 2, we deduce that for $\theta \ge 0$ fixed, the insurer’s loss is monotone in the share of service cost $\beta$.

### Proposition 1

(Monotonicity of insurer’s loss in $\beta$) Under the conditions of Lemma 2 (self-protection) and under the necessary condition of profitability for the insurer, i.e. if $L_0(\theta ,\beta ) < 0$, $\beta \mapsto L_0(\theta ,\beta )$ is a monotone, non-increasing function for any $\theta \ge 0$.

**Fig. 2:**
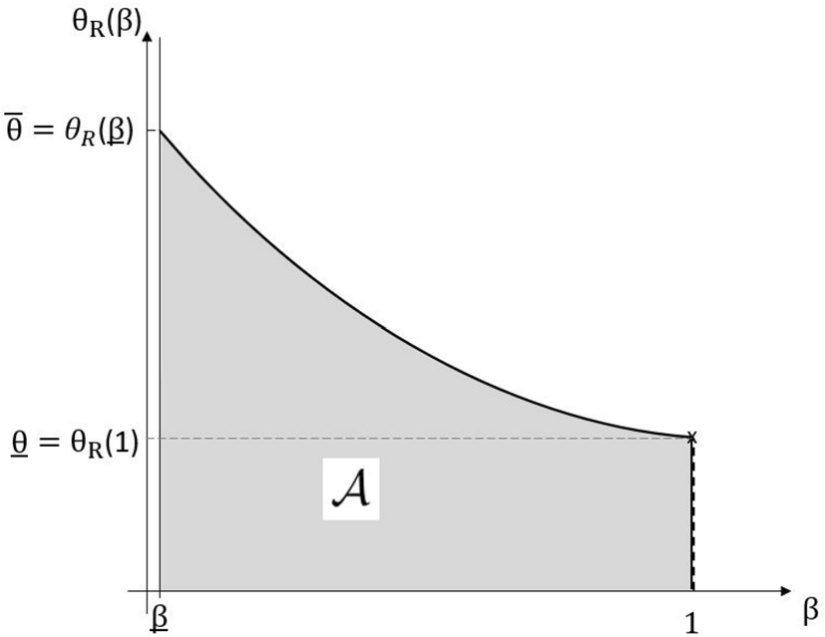
Schematic illustration of the insurer’s admissible set $\mathcal {A}= [0,\theta _R(\beta )] \times [\underline{\beta },1]$ (grey) and the set containing the optimal solution in the self-protection case. According to Proposition 1, an optimal solution must lie on the boundary $\{(\theta _R(\beta ),\beta ), \; \beta \in [\underline{\beta },1]\} \cup \{(\theta ,1),\; \theta \in [0,\underline{\theta }]\}$ (solid black line). Proposition 2 restricts the set containing an optimal solution to the set $\{(\theta ,1),\; \theta \in [0,\underline{\theta }]\}$ (dashed black line). For the special case of a Pareto-distributed loss, the optimal solution $(\theta ^*,\beta ^*) = (\underline{\theta },1)$ is marked by a cross (for details, see section A.5 in the electronic supplementary information)

Proposition 1 states that for any (fixed) loading $\theta$, an optimal solution for the insurer is to choose the maximum possible service cost $\beta _M(\theta )$ acceptable to the buyer, or equivalently that the insurer has no incentive to subsidise risk reduction through a rebate on services. This implies that an optimal solution to problem (5) lies in the (compact) set $\{(\theta ,\beta _M(\theta )),\; \theta \in [\underline{\theta },\bar{\theta }]\} \cup \{(\theta ,1),\; \theta \in [0,\underline{\theta }]\}$ or equivalently $\{(\theta _R(\beta ),\beta ), \; \beta \in [\underline{\beta },1]\} \cup \{(\theta ,1),\; \theta \in [0,\underline{\theta }]\}$ (see Figure 2). The one-dimensional optimisation problem on $\{(\theta _R(\beta ),\beta ), \; \beta \in [\underline{\beta },1]\}$ can be understood as solving the insurer’s trade-off between charging a higher service cost versus a higher risk loading while offering a contract the buyer will accept. The following proposition states that the insurer’s loss on this set is monotone in $\beta$, leading to the statement of Theorem 1.

### Proposition 2

(Monotonicity of insurer’s loss in $\beta$ with maximum feasible risk premium) Under the conditions of Lemma 2 (self-protection), the map $\beta \mapsto L_0(\theta _R(\beta ),\beta )$ is non-increasing.

### Remark 11

(Self-insurance) A central property leading to the above results for the *self-protection* case is non-decreasingness of $s \mapsto \frac{\rho _{s}(X)}{\mathbb {E}_s[X]}$. In case of *self-insurance*, this assumption does not necessarily hold; indeed, for some standard loss distributions (e.g. Pareto, Weibull, or Log-Normal), the converse holds true, i.e. $s \mapsto \frac{\rho _{s}(X)}{\mathbb {E}_s[X]}$ is non-increasing (see Bensalem et al. 2020). In section A.6 in the electronic supplementary information, we study the particular case of a Pareto-distributed loss whose severity is affected by risk reduction service. We find that in this self-insurance case, the insurer can indeed have an incentive to subsidise service cost (i.e. offer contracts with $\beta ^* < 1$), where the optimally subsidised share $(1-\beta ^*)$ increases with the insurer’s risk aversion. In particular, if the risk aversions of insurer and insurance buyer are similar (i.e. $r_0 \searrow r_1$ for the PH transform risk measure), a mutually acceptable contract may only exist if the cost is shared ($0< \beta < 1$). This further implies that the insurer’s optimal solution, i.e. the price structure the insurance buyer is offered, may depend on his choice of risk measure, even if the initial risk assessment is equivalent.

So far, we scrutinised the interaction between the insurer and a single insurance buyer as an isolated problem. This is often reasonable, as in practice insurers usually price individual risks on a stand alone basis without taking into account the existing portfolio. However, the failure of the independence assumption between risks is one of the central challenges in cyber insurance, as cyber incidents at different firms can be dependent, e.g. due to common underlying vulnerabilities (e.g. Böhme et al. 2018; Zeller and Scherer 2022) or due to propagation for worm-type viruses. Therefore, one could argue that rather than finding price structures $(\theta ,\beta )$ by considering problem (5) separately for each customer, the insurer should jointly optimise the risk measure for the entire portfolio against the sum of all premiums received (note that distortion risk measures are in most situations not additive for non-comonotonic risks).

In the “The insurer’s problem: portfolio viewpoint” section, we illustrate that already for portfolios of two dependent losses, the results of Theorem 1 do not necessarily hold anymore, i.e. when optimising from a portfolio viewpoint, indeed the insurer can have an incentive to subsidise self-protection measures for some policyholders.

## The insurer’s problem: portfolio viewpoint

In the self-protection case, a central property is that for any single contract in a portfolio of *n* policyholders with risks $X_i,\; i \in \{1,\ldots ,n\}$, for any feasible loading $\theta _i,\ i \in \{1,\ldots ,n\}$, the reduction in price for increased service outweighs the reduction in the insurer’s risk measure $\rho _{0,s_i}(X_i),\ i \in \{1,\ldots ,n\}$ for each single risk, i.e.

$$\begin{aligned} \underbrace{(1+\theta _i) \frac{\partial \mathbb {E}_{s_i}[X_i]}{\partial s_i}}_{\text {sensitivity of premium for } X_i}  < \underbrace{\frac{\partial \rho _{0,s_i}(X_i)}{\partial s_i}}_{\text {sensitivity of risk measure for } X_i} \le 0,\;\; i \in \{1,\ldots ,n\}. \end{aligned}$$

However, ordering of the relevant sensitivities is not necessarily preserved in a portfolio context, i.e. when adding a new policyholder to an existing portfolio, the reduction of the overall *portfolio risk measure* $\rho _{0,{\textbf {s}}}(X)$ may outweigh the price reduction of the additional contract, i.e. for some $i \in \{1,\ldots ,n\}$: 

7 $$\begin{aligned} \frac{\partial \rho _{0,{\textbf {s}}}(X)}{\partial s_i}< (1+\theta _i) \frac{\partial \mathbb {E}_{s_i}[X_i]}{\partial s_i} < \frac{\partial \rho _{0,s_i}(X_i)}{\partial s_i} \le 0, \end{aligned}$$

where ${\textbf {s}}:= (s_1,\ldots ,s_n)$ and $X = \sum _{i=1}^{n} X_i$ is the aggregated loss. This may imply a situation where the insurer has an economic incentive to subsidise risk reduction for some policyholders in the self-protection case, as we will now analyse in a toy example of two policyholders with dependence mechanisms representative for cyber risk: (directed) loss propagation, common cyber events, and copula approaches. While these bivariate examples will already be sufficient to work out the structural difference to the univariate case, we provide one exemplary extension to a general multivariate setting in section A.7.3 of the electronic supplementary information.

### (Directed) loss propagation

A popular way of modelling dependencies between cyber losses is to consider a model of epidemic spreading in an underlying network, i.e. a directed or undirected graph whose nodes are interpreted as companies (or machines) and whose edges are interpreted as connections between these companies (or machines) through which a state of “infectiousness” can be passed on. These models, often originating from mathematical biology, have been extensively studied in the cyber context over the last few years, see, e.g. Fahrenwaldt et al. (2018), Xu and Hua (2019), Xu et al. (2015) or the surveys Marotta et al. (2017), and Kerstin Awiszus et al. (2022). Interpretations of such models are worm-type viruses spreading between connected machines or a state of business interruption propagating through a supply chain.

#### Example 4

(Bivariate model with one directed edge) For illustration purposes, we consider a portfolio of two firms with one directed edge between them and we understand the “infected” state as a loss occurrence, i.e. assume a loss occurrence in firm 1 can cause a loss in firm 2 with probability $q \in [0,1]$, but not vice versa.^22^ If a loss occurs, the loss sizes are deterministic; w.l.o.g. $0< L_1 \le L_2 < \infty$. We assume that the events of the occurrence of a loss in firm 1, its propagation, and the occurrence of a non-propagated loss in firm 2 are independent. This implies that, depending on the chosen service levels $s_i,\; i \in \{1,2\}$, the loss r.v.s $X_i,\; i \in \{1,2\}$, take the values

$$\begin{aligned} X_1&= {\left\{ \begin{array}{ll} 0 \;\; \text { w.p. } 1-p_1(s_1),\\ L_1 \;\; \text { w.p. } p_1(s_1), \end{array}\right. } X_2 = {\left\{ \begin{array}{ll} 0 \;\; \text { w.p. } 1 - (p_2(s_2) + q p_1(s_1) (1-p_2(s_2))),\\ L_2 \;\; \text { w.p. } p_2(s_2) + q p_1(s_1) (1-p_2(s_2)), \end{array}\right. } \end{aligned}$$

where $s\mapsto p_i(s)$ are continuous, non-increasing functions with $\displaystyle \lim _{s \rightarrow \infty } p_i(s) > 0$ for $i \in \{1,2\}$. Let $X := X_1 + X_2$ denote the portfolio loss, such that the insurer’s portfolio risk measure, using $\psi (u) = u^{r_0},\; {r_0} \in (0,1]$, is given by (see section A.7.1 in the electronic supplementary information):

$$\begin{aligned} \rho _{0,{\textbf {s}}}(X)&= L_1 [(p_1 + p_2 - p_1 p_2)^{r_0} + (p_1 q + p_1 p_2 - p_1 p_2 q)^{r_0}] + (L_2- L_1) (p_2 + p_1 q - p_1 p_2 q)^{r_0}, \end{aligned}$$

where the dependence on $s_i,\; i \in \{1,2\}$, is suppressed for notational convenience and ${\textbf {s}}:= (s_1,s_2)$.

**Fig. 3:**
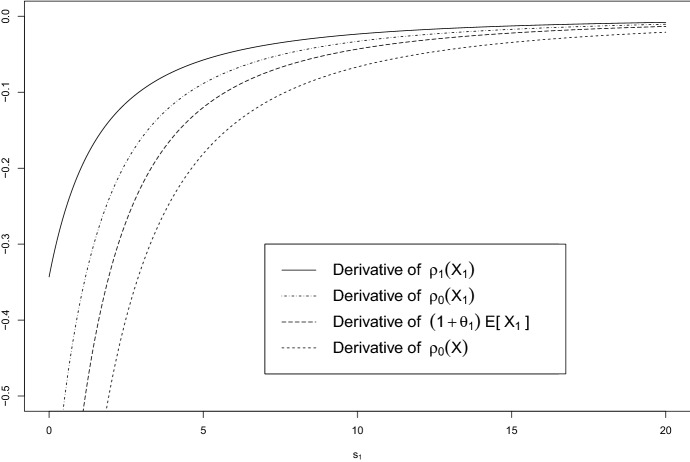
Comparison of derivatives with respect to $s_1$ of single-contract and portfolio risk measures as well as the price of insurance (at a feasible loading $\theta _1 = 0.35$). Note that Equation (7) holds: The decrease in price outweighs the decrease in both single-contract risk measures, but is outweighed by the reduction in the insurer’s portfolio risk measure. The parameters for this example are chosen as $r_0 = 0.8,\; r_1 = r_2 = 0.3,\; L_1 = 5,\; L_2 = 10,\; p_1(s_1) = \frac{1}{a+s_1}+b = \frac{1}{2.5 + s_1} + 0.2,\; p_2 = 0.3,\; q = 0.8$

Figure 3 illustrates that (7) may hold in the above example, which indicates that the insurer can have a financial incentive to subsidise service.

#### Remark 12

(Insurer’s problem: individual optimisation) If the insurer evaluates the two contracts individually, she solves separately

8 $$\begin{aligned} \begin{aligned} \min _{(\theta _i,\beta _i) \in [0,\theta _{R,i}(\beta _i)] \times [\underline{\beta },1]} L_{0,i}^{\text {ind}}(\theta _i,\beta _i) =&\rho _{0,s_{Ii}(\theta _i,\beta _i)}(X_i) - (1+\theta _i)\mathbb {E}_{s_{Ii}(\theta _i,\beta _i)}[X_i] + (1-\beta _i) c(s_{I,i}(\theta _i,\beta _i)),\; i \in \{1,2\}, \end{aligned} \end{aligned}$$

where the superscript ‘ind’ denotes *individual* contract pricing.

#### Remark 13

By very similar calculations as for the Pareto case, one can show that for a loss of deterministic severity, $\theta \mapsto L_{0,i}(\theta ,1)$ is monotone non-increasing, such that the insurer’s optimal solution to the minimisation problems (8) is $(\theta ^*_i,\beta ^*_i) = (\theta _{R,i}(1),1),\; i \in \{1,2\}$, i.e. to shift the full cost of service to the buyers and charge the maximum feasible loading, respectively.

We now consider her optimisation problem from a portfolio viewpoint in a two-contract set-up, where, interestingly, it has to be distinguished whether the contracts with the buyers are closed sequentially or simultaneously. Let us commence by assuming that the two contracts are closed sequentially and firm 2 is insured first.

#### Example 5

(Interpretation of sequential contract closure) Sequential contract closure could be interpreted as a situation where for a prospective policyholder, a loss could be caused by an occurrence at another firm (e.g. a supplier) outside the insurer’s portfolio, but insuring the other firm is not feasible (yet).

#### Remark 14

(Insurer’s problem: sequential optimisation, first policy) The results for firm 2, being insured first, are analogous to the single-contract case: In her initial risk assessment, assume the insurer correctly assesses the loss probability (given service level $s_2$) as

9 $$\begin{aligned} \mathbb {P}_{{\textbf {s}}}(X_2 = L_2) = p_2(s_2) + q p_1(s_1) \big (1-p_2(s_2)\big ), \end{aligned}$$

which depends (due to loss propagation) on the unknown loss probability of firm 1.^23^ For this study, we assume that firm 1 has not subscribed to insurance yet, but has solved the minimisation problem for the no-insurance case correctly, such that in Eq. (9) we set $s_1 = s_{N1}$. As remarked above, we know that the solution to the insurer’s problem (8) for $i=2$ is given by $(\theta ^*_2,\beta ^*_2) = (\theta _{R,2}(1),1)$ and given (9), we can proceed analogously to Sect. 3 to deduce firm 2’s optimal service level without insurance $s_{N2}$ and $s_{I2}(\theta ^*_2,\beta ^*_2)$ within insurance.

The striking observation is as follows: By incentivising a higher service level in a subsequent contract with firm 1, the insurer not only improves the to-be-insured risk in that contract, but also the already priced risk in the existing contract with firm 2, as the probability for a propagated loss decreases.^24^

#### Remark 15

(Insurer’s problem: sequential optimisation, second policy) If the insurer prices each contract as if the risks were independent (or the propagation potential is undetected), she would solve (8) for $i=1$ yielding $(\theta ^*_1,\beta ^*_1) = \big (\theta _{R,1}(1),1\big )$. However, if she correctly takes the effect on the portfolio risk into account, to find $(\theta ^*_1,\beta ^*_1)$ she instead considers the problem

10 $$\begin{aligned} \begin{aligned} \min _{(\theta _1,\beta _1) \in [0,\theta _{R,1}(\beta _1)] \times [\underline{\beta },1]} L_{0,1}^{\text {seq}}(\theta _1,\beta _1) =&\rho _{0,s_{I1}(\theta _1,\beta _1),s_{I2}(\theta _{R,2}(1),1)}(X)\\&- (1+\theta _1)\mathbb {E}_{s_{I1}(\theta _1,\beta _1)}(X_1) - (1+\theta _2)\mathbb {E}_{s_{I2}(\theta _{2,R}(1),1)}(X_2)\\&+ (1-\beta _1) c(s_{I1}(\theta _1,\beta _1)) + (1-\beta _2) c(s_{I2}(\theta _{2,R}(1),1)), \end{aligned} \end{aligned}$$

where the superscript ‘seq’ denotes *sequential* contract closure and ${X} = {X}_{1} + {X}_{2}.$^25^

#### Remark 16

Sequential contract closure in the reverse order can be studied analogously. It is, however, obvious from the set-up of directed loss propagation that the insurer has no additional incentive to subsidise service for firm 2, independently of whether firm 1 is part of the portfolio, i.e. this analysis would not yield different results from the single-contract case and is, thus, omitted.

We now assume that both contracts are priced simultaneously.

#### Example 6

(Interpretation of simultaneous contract closure) In practice, simultaneous contract closure could be interpreted as two firms jointly inquiring about insurance (e.g. companies along a supply chain or parent company and subsidiary) or the insurer approaching both before the first contract is closed.

#### Remark 17

(Insurer’s problem: simultaneous optimisation) If the insurer offers both contracts simultaneously, she considers the four-dimensional problem

11 $$\begin{aligned} \begin{aligned} \min _{(\theta _1,\beta _1,\theta _2,\beta _2) \in \mathcal {A}} L_{0}^{\text {sim}}(\theta _1,\beta _1,\theta _2,\beta _2)&= \rho _{0,s_{I1}(\theta _1,\beta _1),s_{I2}(\theta _1,\beta _1,\theta _2,\beta _2)}(X)\\&-(1+\theta _1)\mathbb {E}_{s_{I1}(\theta _1,\beta _1)}[X_1] - (1+\theta _2)\mathbb {E}_{s_{I2}(\theta _1,\beta _1,\theta _2,\beta _2)}[X_2]\\&+(1-\beta _1) c(s_{I1}(\theta _1,\beta _1)) + (1-\beta _2) c(s_{I2}(\theta _1,\beta _1,\theta _2,\beta _2)), \end{aligned} \end{aligned}$$

where the superscript ‘sim’ denotes *simultaneous* contract closure, ${X} = {X}_{1} + {X}_{2},$ and $\mathcal {A}:= [0,\theta _{R,1}(\beta _1)] \times [\underline{\beta },1] \times [0,\theta _{R,2}(\beta _2)] \times [\underline{\beta },1]$ is the admissible set for this problem.

**Fig. 4:**
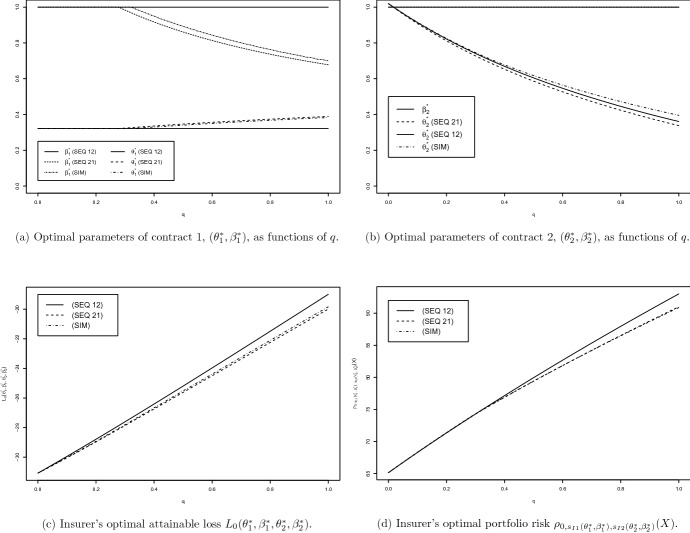
Aspects of the insurer’s solution in the portfolio case with directed loss propagation. The parameters for this example are: loss sizes $L_1=50, L_2 = 100$, loss probability parameters $a_1=a_2=2.5, b_1=b_2=0.2$, risk aversion $r_0=0.8,r_1=0.7,r_2=0.3$, cost parameters $\eta = 0.5,\gamma =2,\beta _o=1.1$, $q \in [0,1]$

The results of numerically solving the above optimisation problems are given in Fig. 4 for the propagation probability $q \in [0,1]$, which in this set-up governs the dependence between the risks.^26^

#### Remark 18

(Interpretation of results for directed loss propagation)

- **Panel** **4****(a)** depicts the optimal pricing parameters $(\theta _1^*,\beta _1^*)$ of the contract offered to firm 1 (the “source of propagation”). If the contract with firm 2 is priced first, the insurer may subsidise service (i.e. choose $\beta ^* < 1$) in the subsequent contract with firm 1, as this reduces the insured risk in contract 2 (without having to adjust the premium of firm 2). This subsidy $(1-\beta ^*)$, as well as the loading $\theta ^*_1$, increase with the dependence between the risks. The same effect occurs, but to a smaller extent, if the contracts are priced simultaneously. This is caused by the fact that by subsidising service for firm 1, the insured risk in firm 2 is reduced, but this now has to be reflected in a decreased chargeable premium for that contract. Therefore, the incentive to subsidise service for firm 1 is smaller relative to the case where the price of contract 2 is fixed first.
- **Panel** **4****(b)** depicts the optimal parameters $(\theta _2^*,\beta _2^*)$ of the contract offered to firm 2. As the service level of firm 2 has no additional effect on firm 1, the insurer’s problem for firm 2 is always analogous to the single-contract case, and thus, service cost is never subsidised ($\beta ^*=1$). However, the risk loading depends on the loss probability $\mathbb {P}_{\textbf {s}}(X_2=L_2)$, which differs between the cases as it depends on $s_1^*$ and therefore on whether firm 1 is insured already (and under which parameters).
- **Panel** **4****(c)** depicts the insurer’s optimally attainable negative loss (gain) $L_0(\theta _1^*,\beta _1^*,\theta _2^*,\beta _2^*)$, which decreases with increasing dependence between the risks, while the additional gain from pricing contracts “correctly”, i.e. using the portfolio risk measure, increases with the dependence. Analogous observations hold for the insurer’s portfolio risk, see Panel 4(d).

### Cyber events at multiple ‘targets’

Another way to understand dependence between cyber losses is to consider the presence of common (*systemic*) vulnerabilities which allow cyber threats to affect multiple companies simultaneously (see, e.g. Böhme et al. 2018; Zeller and Scherer 2022). Realistic examples for systemic events causing incidents in multiple firms are the accidental outage or the malicious exploitation of a vulnerability in commonly used software or operating systems, leading to, e.g. data breaches or fraudulent activity (e.g. ransomware claims).^27^

#### Remark 19

(Buyer’s vs. insurer’s perspective on common events) In this setting, each company faces incidents from systemic events as well as *idiosyncratic* incidents occurring independently from other firms, e.g. the loss or theft of hardware or negligent employee behaviour leading to involuntary data disclosure or business interruption. From the viewpoint of each company (insurance buyer), both types of incidents are indistinguishable in the sense that they aggregate to one loss arrival process, i.e. the company simply monitors if a loss occurs (disregarding its source) without knowing (or caring) if others may be simultaneously affected. From the insurer’s portfolio viewpoint, however, the two types of incidents are viewed differently: incidents from systemic events are particularly worrisome as they entail accumulation risk, whereas idiosyncratic incidents are “desirable” in the sense that they constitute (if correctly priced) the basis of the insurance business and can be “diversified away” in a large portfolio.

#### Example 7

(Bivariate model with common events) Consider as model for the risks $X_1$ and $X_2$:

$$\begin{aligned} X_1&= L_1 \mathbf {1}_{\{\min \{E_1,E_{12}\}\le T\}}, \; X_2 = L_2 \mathbf {1}_{\{\min \{E_2,E_{12}\}\le T\}}, \end{aligned}$$

with $E_1 \sim Exp(\lambda _1),\; E_2 \sim Exp(\lambda _2)$, and $E_{12} \sim Exp(\lambda _{12})$ independent with $\lambda _{1},\lambda _{2},\lambda _{12} \ge 0,\; s.t.\; \lambda _{i}+\lambda _{12} > 0,\; i \in \{1,2\}$, and w.l.o.g. $0< L_1 \le L_2 < \infty$. $E_1$ and $E_2$ model the arrival times of an idiosyncratic incident to firm 1 and 2, respectively, whereas $E_{12}$ models the arrival time of a common event causing simultaneous incidents in both firms, with deterministic loss sizes $L_1$ and $L_2$, respectively. Let *T* denote the time horizon of the policy under consideration (w.l.o.g. $T=1$ in what follows) and let

$$\begin{aligned} \lambda _I&:= \lambda _1+\lambda _{12}, \; \lambda _{II} := \lambda _2+\lambda _{12}, \end{aligned}$$

denote the overall marginal arrival rates of incidents to firms 1 and 2, respectively.^28^ It follows that the buyers’ risk measure and expected loss are given by

$$\begin{aligned} \rho _1(X_1)&= L_1 (1-e^{-\lambda _I})^{r_1},\; \mathbb {E}[X_1] = L_1 (1-e^{-\lambda _I}),\\ \rho _2(X_2)&= L_2 (1-e^{-\lambda _{II}})^{r_2},\; \mathbb {E}[X_2] = L_2 (1-e^{-\lambda _{II}}), \end{aligned}$$

while the insurer’s portfolio risk measure is given by (see section A.7.2 in the electronic supplementary information)

$$\begin{aligned} \rho _0(X)&= L_1 [(1-y_{00})^{r_0} + (1-(y_{00}+y_{10}+y_{01}))^{r_0}] + (L_2-L_1) (1-(y_{00}+y_{10}))^{r_0}, \end{aligned}$$

where $y_{00} := e^{-(\lambda _1+\lambda _2+\lambda _{12})},\; y_{10} := (1-e^{-\lambda _1}) e^{-(\lambda _2+\lambda _{12})},\; y_{01} := (1-e^{-\lambda _2}) e^{-(\lambda _1+\lambda _{12})}$ are the probabilities of none (subscript $_{00}$) or exactly one (subscripts $_{10}$ and $_{01}$) of the companies experiencing a loss.^29^

#### Remark 20

(Interpretation: Self-protection by prevention of systemic events) We now consider the effect of self-protection services which can be distinguished into different categories described in Table 4. In the following, we scrutinise one possible type of effect we regard as particularly interesting in the cyber context, namely the prevention of systemic events: as the existence of common vulnerabilities (e.g. use of the same software) is regarded as the source of dependence between losses, it is firstly crucial for a cyber insurer to identify such common factors among policyholders and offer services which prevent the manifestation of a loss from a systemic event for the policyholder himself (e.g. timely patch management for standard software). Second, it is in the insurer’s interest to use knowledge about an incident (or so-called *near miss*, i.e. a threat that did not lead to an incident due to adequate controls) at one insured company to immediately warn other policyholders about the imminent threat and, thus, hopefully increase the chance of averting a loss manifestation for them. Thus, the total portfolio loss in case of a systemic event could be reduced or, if **all** policyholders are warned on time, the manifestation of the systemic event could even be prevented.^30^

#### Remark 21

(Insurer’s problem: sequential optimisation, first policy) Assume again sequential contract closure, where w.l.o.g. the contract with firm 2 is closed first and its chosen service level affects the rate $\lambda _{II}$ via a decreasing map $s_2 \mapsto \lambda _{II}(s_2)$.^31^ Recall that by Lemma 3 (see section A.3 in the electronic supplementary information) a sufficient condition for convexity of the insurance buyer’s optimisation problem is to choose the map $s_2 \mapsto \lambda _{II}(s_2)$ in such a way that the subjective loss probability $s_2 \mapsto \psi _2\big (\mathbb {P}_{{\textbf {s}}}(X_2 = L_2)\big ) = (1-e^{-\lambda _{II}(s_2)})^{r_2}$ is convex. For simplicity, we choose analogously to above (however, for the rate, not the loss probability directly)

$$\begin{aligned} \lambda _{II}(s_2) = \frac{1}{s_2 + a_2}+b_2, \end{aligned}$$

with $a_2,b_2 > 0$ such that the above convexity condition is fulfilled.

With the contract closure of firm 2, the insurer solves the single-contract problem (8) for $i=2$, resulting in $(\theta _2^*,\beta _2^*) = \big (\theta _{R,2}(1),1\big )$ and within-insurance service level $s^*_2 = s_{I2}\big (\theta _{R,2}(1),1\big )$ determining the loss probability of firm 2 via the rate $\lambda _{II}(s_2^*)$.

#### Remark 22

(Insurer’s problem: sequential optimisation, second policy) At subsequent contract offering to firm 1, we assume that the service level of firm 1 influences the rate $\lambda _{I}(s_1)$ via a decreasing map

$$\begin{aligned} s_1 \mapsto \lambda _{12}(s_1) = \frac{1}{s_1 + a_{12}} + b_{12}, \end{aligned}$$

with $a_{12},b_{12} > 0$ such that $s_1 \mapsto \psi _1\big (\mathbb {P}_{{\textbf {s}}}(X_1 = L_1)\big )$ is convex and it must hold $\lambda _{12}(s_1) \le \lambda _{II}(s_2^*)$ for any $s_1 \ge 0$. The marginal rates for both firms are then given by (now the incidents can be classified as idiosyncratic or systemic)

$$\begin{aligned} \lambda _{I}(s_1)&= \lambda _{1} + \lambda _{12}(s_1),\\ \lambda _{II}(s_1,s_2^*)&= \lambda _{2}(s_2^*) + \lambda _{12}(s_1), \end{aligned}$$

for some constant $\lambda _1 > 0$, implying that the choice of $s_1$ affects the marginal distributions of both risks as well as the dependence between them, e.g. expressed by $s_1 \mapsto 1-\frac{\lambda _{1}}{\lambda _{I}(s_1)}$.^32^ Therefore, when offering a contract to firm 1, the insurer should again consider problem (10) to correctly take the dependence into account, as opposed to solving (8) for $i=1$.

#### Remark 23

**Fig. 5:**
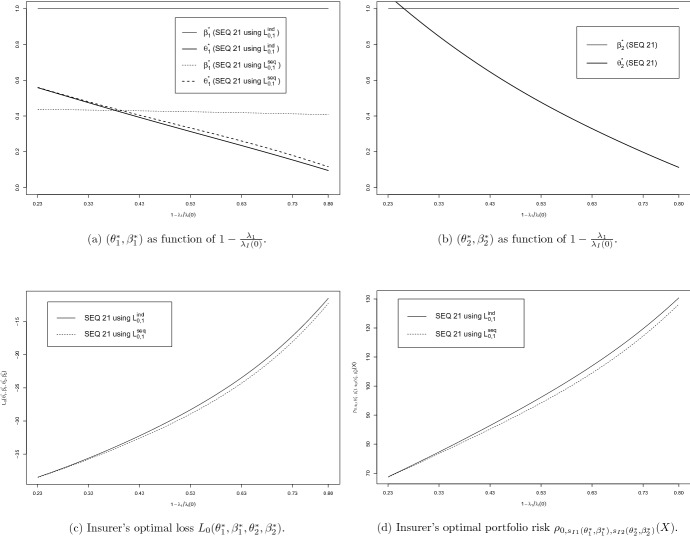
Aspects of the insurer’s solution in the portfolio case with common cyber events. The parameters for this example are: $L_1=50, L_2 = 100$, $\lambda _1 = 0.5$, $r_0=0.8,r_1=0.4,r_2=0.3$, $\eta = 0.5,\gamma =2,\beta _o=1.1$, with $\lambda _{12}(0) = \frac{1}{a_{12}}+ b_{12} \in [0.15,2]$ and $\frac{\lim _{s \rightarrow \infty } \lambda _{12}(s)}{\lambda _{12}(0)} = \frac{1}{2}$ for any starting value

(Results for prevention of systemic events) Numerical results of solving (10) are given in Fig. 5 for varying degree of dependence between the two risks.^33^ We observe that if the contract of firm 1 is priced using (10), it can be optimal for the insurer to choose $\beta _1^* < 1$, leading to an increased risk loading, an increased optimal service level $s_{I1}$ within the insurance policy, a decreased loss probability for both policyholders, and an increased gain and decreased portfolio risk for the insurer. These effects increase with the dependence between the two risks.^34^

### Copula approaches

*Copula* approaches have become a widely popular method to assess and describe dependence between random variables, as they allow the decomposition of a multivariate distribution function (c.d.f.) *F* of a random vector $(X_1,\ldots ,X_d)$ into marginal c.d.f.s $F_1,\ldots ,F_d$ and an object representing the *dependence structure*, called copula *C*, which itself is a multivariate c.d.f. with standardized uniform marginals (see section A.2 in the electronic supplementary information). In empirical research on cyber-risk modelling, one starts with observations of cyber losses that are conjectured not to be independent. As the main goal of many empirical studies is the description and analysis of the observed data, *bottom-up* approaches that seek to mimic the mechanism underlying the dependence between cyber losses may not be available for a statistical investigation, yet. Rather, a *top-down* approach of analysing the multivariate observations by fitting (parametrically or non-parametrically) univariate distributions to the marginals and by choosing a flexible parametric copula family and fitting its parameter(s) to the observed data, is often preferred (due to numerical tractability).

In the cyber context, e.g. Eling and Jung (2018) study the cross-sectional dependence of data breach losses (cross-industry and cross-breach type) using a *Gaussian* copula, among others. Previously, Böhme and Kataria (2006) and Herath and Herath (2011) proposed models for cyber risk using the *t-copula* and the *Archimedean copula family (Clayton and Gumbel)*, respectively. More recently, Peng et al. (2018) studied the multivariate dependence exhibited by real-world cyber attack data using a Copula-GARCH model with *vine copulas*.

#### Example 8

(Bivariate Gumbel copula) An example akin to the ones above would be for the bivariate case $(X_1,X_2) \sim F_{\textbf {s}}$ with

$$\begin{aligned} F_{\textbf {s}}(x_1,x_2) = C_{\theta ({\textbf {s}})}\big (F_{1,s_1}(x_1),F_{2,s_2}(x_2)\big ),\;\; x_1,x_2 \in \mathbb {R}, \end{aligned}$$

where $F_{i,s_i}$ are the marginal c.d.f.s of the single risks depending on the chosen service levels $s_i$ (for example, *zero-inflated Pareto distributions* as considered in Appendix 7.5 in elctronic supplementary information) and $C_{\theta ({\textbf {s}})}(u,v)$ is the bivariate Gumbel copula (see Gumbel 1960)

$$\begin{aligned} C_{\theta ({\textbf {s}})}(u,v) = {\textstyle \exp \!\left[ -\big ((-\ln (u))^{\theta ({\textbf {s}})}+(-\ln (v))^{\theta ({\textbf {s}}) }\big )^{1/\theta ({\textbf {s}})}\right] },\;\; \theta ({\textbf {s}}) \in [1,\infty ),\; u,v \in [0,1], \end{aligned}$$

which seems a suitable choice in the cyber-risk context as it allows for capturing *upper tail dependence* and is the only member of the Archimedean family which is also an *extreme-value copula*.^35^ The dependence is governed by the parameter $\theta ({\textbf {s}}) \in [1,\infty )$, ranging between the *independence* copula for $\theta ({\textbf {s}}) = 1$ and perfect positive dependence (i.e. converging to the *comonotonicity* copula) for $\theta ({\textbf {s}}) \rightarrow \infty$.^36^

#### Remark 24

(Effects of service on portfolio risk in the copula setting) Again, different assumptions about how the chosen service levels ${\textbf {s}}= (s_1,s_2)$ of insurance buyers influence the (joint) portfolio risk can be postulated:

- If service only influences the marginal distribution of the insured risk, i.e. via $s_i \mapsto F_{i,s_i},\; i \in \{1,2\}$, inducing a decreasing order in the sense of the “Model set-up and assumptions” section, the analysis does not differ from the univariate case. For examples in the cyber context, see the first row of Table 4.
- If service only affects the dependence between the risks via a (in some suitable (partial) ordering decreasing) map ${\textbf {s}}\mapsto \theta ({\textbf {s}})$ without altering the marginals, it is obvious that no insurance buyer would have an economic incentive to purchase such service (compare the last case in Table 4) and another (interesting!) question would arise, namely, how much the insurer should optimally spend on giving away service (as a free addition to risk transfer) to favourably (in her risk measure) alter the dependence structure of her portfolio.
- If service affects both the marginal distribution(s) and the dependence structure, an example where both parties agree to share the cost of service could be constructed. For interpretations in the cyber context, compare the second and third row of Table 4.

As remarked above, however, the main drawback of such a top-down modelling approach is that it is not based on an attempt to causally understand the dependence between cyber losses; instead, its merit is based on the analytical decomposition in Theorem 2 (see section A.2 in the electronic supplementary information) and its tractability in statistical inference. This is a somewhat questionable foundation in the cyber context due to scarcity, limited reliability, and suspected non-stationarity of available data, limiting the informativeness of models estimated on past data for the prediction of future losses. Therefore, we do not go into more detail on this example, but reiterate that in principle it provides the same flexibility regarding the effect of risk reduction services in insurance policies as the examples treated in detail above.

## Conclusion

In recent years, with demand for cyber insurance increasing tremendously, cyber insurance markets around the world have been growing and the range of available cyber policies has been continuously expanding. As policies continue to mature, many prospective insurance buyers and external cyber experts agree that pure risk transfer cannot be an optimal cyber-risk management solution. Instead, companies – insured or not – have to make ongoing efforts to keep their cybersecurity measures up-to-date, given the evolving cyber-threat landscape. Therefore, there is mutual benefit (for all stakeholders) in the combination of risk transfer and risk reduction measures, leading to the (prospective) ubiquitous offering of pre-incident and post-incident services.

In this study, we have dealt with this combination of risk reduction and risk transfer in the cyber insurance context, and in particular addressed the question of how such risk reduction services should be optimally priced from an insurer’s viewpoint. We have illustrated how common services within cyber insurance can be classified into the concepts of self-protection and self-insurance, and have argued how insurers should make use of their unique position regarding knowledge about the current cyber-loss landscape to offer additional pre-incident (warning) services to their policyholders.

We have shown that in the univariate case, i.e. when pricing a single contract alone, an insurer using a distortion risk measure with concave distortion (i.e. being risk-neutral or risk-averse) never has an economic incentive to subsidise pure self-protection services (i.e. only considering the effect on loss probability, factoring out potential cross-effect on loss sizes and the prospect of gaining additional information) and will, thus, always shift their full cost to the insurance buyer. Interestingly, this does not generally hold for the pricing of self-insurance services or when taking a multivariate (portfolio) viewpoint, in which case it can be optimal (and in some cases even mandatory to find an acceptable contract for both parties) to share the cost of risk reduction service between insurer and policyholder. We illustrate this finding using toy examples of two risks with dependence mechanisms representative for the cyber context and one exemplary extension to a larger multivariate setting.

From the insurance buyers’ point of view, the study serves to illustrate how their initial risk (when approaching the insurer) and their choice of (distortion) risk measure as well as the existing portfolio of the insurer can influence the insurance price offered to them for different contracts (i.e. how much risk reduction is implicitly incentivised for them by the insurer’s choice of price structure).

Some interesting aspects, however, remain for future research. We restricted the insurance buyer’s options to full or no insurance (as is customary for primary insurance in the cyber context), but one could extend this to more general payout functions (e.g. proportional at any share $\alpha \in [0,1]$ or excess-of-loss per risk at different priorities and limits).^37^ Furthermore, we have mentioned that in the cyber context, part of the risk should be considered non-insurable (e.g. reputational risk), an aspect that could generalize the modelling of the insurance buyer’s optimisation problem.

From the insurer’s point of view, the pricing of self-protection and self-insurance services has been studied disjointly, whereas in practice, the combination of both types of services within a policy is customary. Furthermore, we have only illustrated the insurer’s portfolio viewpoint in bivariate examples and an exchangeable extension. Fully exploring the question of optimal offering of cyber services using an insurer’s more general multivariate viewpoint on a portfolio of dependent policyholders comprises many interesting questions for future work.

Furthermore, especially due to the potential for extreme cyber losses resulting from single large losses or accumulation risk from a large cyber event, many insurers work with reinsurance providers to limit their exposure and manage their portfolio risk. This opens the potential to analyse a suitable Stackelberg game between insurer and reinsurer(s) or even a set-up involving all three parties (insurance buyer(s), insurer, and reinsurer(s)). In this context, also interesting questions about optimal risk sharing arise.

Lastly, we have argued that the understanding of the dependence between cyber losses is crucial for insurers, as purely top-down dependence modelling approaches may not be suitable in the highly dynamic, non-stationary cyber domain. Therefore, more empirical research on the dependence structures underlying cyber risk, e.g. to more accurately determine underlying common factors leading to simultaneous exposure to a certain cyber event, is certainly necessary to better understand the evolving cyber-threat landscape. Lastly, it should be mentioned that many related questions from a not purely mathematical viewpoint arise. For example, economically and legally, it needs to be investigated how to ideally set up cyber insurance policies including services such that all parties (insurer, insureds, and IT security experts as service providers) draw synergies from the collaboration. From a technical viewpoint, one important issue is how to effectively quantify (and monitor) the IT security landscape of a potentially highly complex enterprise for actuarial applications. These issues emphasise the importance of interdisciplinary collaboration and research in the cyber insurance domain in order to tackle this challenging risk. This article is complemented by an electronic supplement (Appendix) containing a seminal discussion of risk-assessment services, mathematical preliminaries, proofs, case studies and extended calculations.

**Table 2:** Overview of parameters in problems (BP) and (IP)

Parameter	Range	Interpretation	Buyer’s view	Insurer’s view	Comment
$\alpha$	$\{0,1\}$	Insurance share (opt-in $(=1)$ / opt-out $(=0)$)	Optimisation parameter, choose $\alpha ^*$	$\alpha ^*$ chosen by buyer	
*s*	$[0,\infty )$	Cyber service level	Optimisation parameter, choose $s^*$	$s^*$ chosen by buyer	
$s_B(\theta ,\beta )$	$(0,\infty )$	Boundary between sets of values of *s* where no insurance vs. full insurance is preferred in the self-protection case	Fixed	Fixed given choice of $(\theta ,\beta )$	See Corollary 1 and Remark 6.
$s_{B1/B2}(\theta ,\beta )$	$(0,\infty )$	Boundaries between sets of values of *s* where no insurance vs. full insurance is preferred in a special self-insurance case	Fixed	Fixed given choice of $(\theta ,\beta )$	See section A.6 in the electronic supplementary information.
$s_N$	$[0,\infty )$	Optimal service demand without insurance	To be found by minimising $L_{1,\mathcal {N}}(s)$ (loss without insurance)	Fixed	See Corollary 2 and Remark 7.
$s_I(\theta ,\beta )$	$[0,\infty )$	Optimal service demand with insurance	To be found by minimising $L_{1,\mathcal {I}}^{\theta ,\beta }(s)$ (loss with insurance)	Fixed given choice of $(\theta ,\beta )$	See Corollary 2 and Remark 7.
$\theta$	$[0,\infty )$	Risk loading	$\theta ^*$ chosen by insurer	Optimisation parameter, choose $\theta ^*$	
$\theta _0$	$[0,\infty )$	Minimum loading s.t. buyer would choose not to insure original risk	Fixed, constant	Fixed, constant	See Corollary 1 and Remark 6.
$\theta _N(\beta )$	$(\theta _0,\infty )$	Minimum loading s.t. global minimiser of no-insurance problem $s_N$ lies in set where no insurance is chosen	Fixed	Fixed given choice of $\beta$	See Corollary 2 and Remark 7.
$\theta _I(\beta )$	$[0,\infty )$	Maximum loading s.t. pure risk transfer (insurance) is preferred to combination of risk transfer and risk reduction (insurance and service)	Fixed	Fixed given choice of $\beta$	See Corollary 2 and Remark 7.
$\theta _R(\beta )$	$(\theta _N(\beta ),\infty )$	Maximum loading s.t. buyer chooses (full) insurance	Fixed	Fixed given choice of $\beta$	See Corollary 3 and Remark 8; Denote $\underline{\theta } := \theta _R(1),\; \bar{\theta } := \theta _R(\underline{\beta })$, see the "The insurer's problem: single-contract case" section.
$\beta$	$[\underline{\beta },1]$	Share of service cost shifted to buyer	$\beta ^*$ chosen by insurer	Optimisation parameter, choose $\beta ^*$	
$\underline{\beta }$	(0, 1)	Minimum share of service cost to be shifted to buyer	Fixed, constant	Fixed, constant	Service cannot be given away for free.
$\beta _o$	$(1,\infty )$	Share of service cost without insurance	Fixed, constant	Fixed, constant	Service without insurance is more expensive than service combined with insurance.

**Table 3:** Overview of functions in problems (BP) and (IP)

Function	Interpretation	Properties	Comment
$\rho _{0,s}(X)$	Insurer’s risk measure	$X \mapsto \rho _{0,s}(X)$ is a (coherent) distortion risk measure; $s \mapsto \rho _{0,s}(X)$ is convex, continuous, non-increasing, and $\rho _s(X) \ge \mathbb {E}_s[X] > 0$	See Definition 1 and Lemma 1.
$\rho _{1,s}(X)$	Insurance buyer’s risk measure	As for $\rho _{0,s}(X)$	Risk measures should reflect that buyer is more risk-averse than insurer.
*c*(*s*)	Cost of service	$s \mapsto c(s)$ is increasing, strictly convex, and continuous with $c(0)=0$ and $\displaystyle \lim _{s \rightarrow \infty } c(s) = \infty$	Increasingness and convexity are natural economic assumptions.
*p*(*s*)	Loss probability in the self-protection case	$s \mapsto p(s) \in [0,1]$ is decreasing and convex	Decreasingness and convexity are natural economic assumptions. Additionally assume convexity of $s \mapsto \psi (p(s))$ to ensure convexity of problem (BP).

**Table 4:** Different effects of risk mitigation service if systemic events are the source of loss dependence

Scenario	Model	Effect on marginals and dependence			Interpretation
		$s_1 \mapsto \lambda _{I}({\textbf {s}})$	$s_1 \mapsto \lambda _{II}({\textbf {s}})$	$s_1\mapsto 1-\frac{\lambda _{1(2)}({\textbf {s}})}{\lambda _{I(II)}({\textbf {s}})}$	
Prevention of idiosyncratic incidents	$s_1$ affects risk $X_1$ via decreasing map $s_1 \mapsto \lambda _{1}(s_1)$	Decreases	Constant	Increases	Service prevents idiosyncratic incidents, e.g. continuous monitoring and improvement of password and access control management to impede unauthorised access to confidential data or processes.
Prevention of systemic events	$s_1$ affects portfolio risk *X* via decreasing map $s_1 \mapsto \lambda _{12}(s_1)$	Decreases	Decreases	Decreases	Service prevents the manifestation of a loss from a systemic event for the policyholder and allows the insurer to prevent a potential loss from the same source in other companies in the portfolio. An example is timely patch management for all common software where additionally all *near misses* are immediately reported to and analysed by the insurer (or her service provider), allowing them to identify current threats and warn other firms.
Transformation of systemic events to idiosyncratic incidents	$s_1$ affects portfolio risk *X* via decreasing map $s_1 \mapsto \lambda _{12}(s_1)$, such that for fixed $s_2$, $s_1 \mapsto \lambda _{II}({\textbf {s}})$ is constant	Decreases	Constant	Decreases	Service makes firm 1 less frequently affected by incidents from systemic events, e.g. improved patch management for common software or usage of different operating systems or cloud service providers, but the risk for other firms in the portfolio is not improved (i.e. all events that would have affected both firms jointly initially now affect firm 2 alone, such that the risk $X_2$ does not change).^a^
	$s_1$ affects portfolio risk *X* via decreasing map $s_1 \mapsto \lambda _{12}(s_1)$, such that $s_1 \mapsto \lambda _{I}({\textbf {s}})$ is constant	Constant	Decreases	Decreases	Service in the contract of firm 1 does not prevent a loss in firm 1, but allows the insurer to warn others. It is obvious that firm 1 has no economic incentive to purchase such service (i.e. any $\beta _1 > 0$ yields $s_{I1}(\theta _1,\beta _1)=0$ for any $\theta > 0$), such that in this case one would have to allow $\beta _1 = 0$ and reformulate the insurer’s optimisation in terms of $\theta _1$ and $s_1$, where $s_1$ is the amount of service the insurer would optimally include for free as part of the policy to optimise her portfolio risk.

^a^Böhme (2005) analyses the similar idea of premium discrimination between users of a dominant and an alternative platform (e.g. representing an operating system) to estimate the extent to which insurance premiums can motivate “ecosystem diversification” and counterbalance market processes that converge to a “monoculture” of installed systems

## Supplementary Information

Below is the link to the electronic supplementary material.

Supplementary file (PDF 2902 KB)

## Footnotes

1 The distinction between self-protection and self-insurance provides good guidance and fits well with simple two-state models and frequency-severity approaches. Note that in reality, the effects of altering loss probabilities and loss sizes are often inseparable, which may be particularly relevant for cyber risks.

2 The cited studies use a continuous-time setting, whereas we consider a one-period model.

3 Indeed, in other insurance domains, if incentive programmes exist (e.g. discounts on health insurance for participating in fitness regimes), they often give rise to moral-hazard issues, i.e. the insurer needs to secure the insured actually complies with the agreed-upon level of effort. In the cyber context, however, moral hazard does not seem to be a major concern for two reasons: first, due to the novelty and dynamics of cyber risk and the high complexity of technical systems, it is likely that neither of the parties (insurer and insured) have a full understanding of the underlying risk, i.e. the main problem is a lack of information for both parties rather than information being withheld. Due to the necessity for up-to-date technical expertise, insurers collaborate with specialised IT service providers to assess and monitor the insured risks and recommend or employ risk mitigation measures. Thus, in our framework, we assume both risk transfer and risk reduction are offered through the insurer (principal), i.e. risk reduction services are part of the insurance contract and therefore their uptake (ex-ante) and upholding (ex-post) observable to the insurer. Second, as e.g. reputational risk from cyber events or losses from threats classified as war actions are not fully insurable but substantial risks in practice, the insured has an intrinsic motivation to mitigate such risks, even if an insurance policy to transfer other financial losses is in place.

4 Such *distortion risk measures* result from the properties of law-invariant, coherent risk measures if the property of sub-additivity for all random variables is replaced by additivity for comonotone random variables (see, e.g. Föllmer and Schied (2016) and Dhaene et al. (2012) for a detailed exposition and Dhaene et al. (2006, 2011) for a general review on (distortion) risk measures and their relation to comonotonicity). The sub-class of distortion risk measures with concave distortion functions used in this study can furthermore be shown to be coherent (see Wirch and Hardy 1999), i.e. are a sub-class of law-invariant, coherent risk measures.

5 While both types of services can have intertwined effects and relate to gaining information via risk assessment services, the issues of moral hazard / asymmetric information and the prospect of gaining additional information are excluded from the mathematical analysis in the main part of this paper. A discussion of how to potentially address the effect of risk assessment services is provided in section A.1 in the electronic supplementary information.

6 We consider a single buyer during the first part of the paper and extend this to examples of two (resp. $N \ge 2$) buyers with dependent cyber-loss occurrences in the “The insurer's problem: portfolio viewpoint” section (resp. section A.7.3. in the electronic supplementary information).

7 This implies that a prospective buyer would be particularly well advised to enquire about prices at different insurers, as the offered price structures may differ depending on the existing portfolio, even if (hypothetically) the insurers’ risk assessment and modelling processes were identical.

8 All of the above considerations emphasise again that moral hazard and information asymmetries might not be a severe problem in cyber as knowledge and incentives are aligned.

9 Naturally, an extension to a setting where both concepts are studied as intertwined remains an interesting task for future research.

10 The parameter *s* denotes the amount of risk mitigation service, whose categories were detailed above.

11 Throughout, we use the term *non-decreasing* for a real-valued function that fulfils $\forall x,y: x < y \implies f(x) \le f(y)$ and *increasing* if the order in the implication is strict. The terms *non-increasing* and *decreasing* are used analogously.

12 A distortion function $\psi : [0,1] \rightarrow [0,1]$ is a continuous, non-decreasing function with $\psi (0)=0$ and $\psi (1)=1$. The distortion is often economically interpreted as a subjective weighting of objective probabilities representing the decision maker’s views or risk preference.

13 By the properties of the Choquet integral (see Denneberg 2013), any distortion risk measure fulfils 1., 2., 4., and 5. in Definition 1  (Section A.2 of the electronic supplementary information) and additionally 3. if the distortion function $\psi$ is concave (and the underlying probability space has no atoms), see, e.g. Wirch and Hardy (1999).

14 $F_0$ denotes the loss distribution of the buyer given his initial characteristics, including his existing IT security measures. The subscript 0 indicates that no *additional* services to reduce the risk have yet been acquired following the initial risk assessment. As the initial IT security level (and other characteristics) vary between prospective buyers, the initial risk assessment yields inhomogeneous $F_0$. Note that for some companies, the risk assessment as part of the insurance take-up process may be the first comprehensive analysis of the cybersecurity level of their organization. While not every inquiry about insurance prices leads to the closure of a cyber insurance contract, the process may serve as a wake-up call for the acquisition of (additional) risk reduction measures within or without an insurance policy.

15 One might argue that *s* should rather be chosen from a discrete set $\{s_1,\ldots ,s_n\},\; n \in \mathbb {N}$ (a potentially interesting combinatorial optimisation problem), representing all feasible combinations of service packages offered by the insurer. This is reasonable and we regard this as a mathematically different version of the problem whose analysis is not the present focus.

16 As $\beta$ does not depend on *s* (a potential generalisation for future studies), we do not allow the insurer to give away service for free, as otherwise the cost of service $\beta c(s)$ would not increase with its amount, which is unnatural.

17 The latter option is not necessarily feasible in practice, as the insurer may not be interested in or legally allowed to sell such services.

18 If one does not want to allow the interpretation that such contracts are offered by the insurer outside of an insurance policy (e.g. due to legal restrictions), the insurer’s loss function should be formulated in a way that makes these contracts unprofitable (e.g. as done here by restricting $\beta \in [\underline{\beta },1]$). If one wants to allow such contracts (one could argue that such a contract could be closed in the cyber domain with a client that has other contracts with the same insurer), a choice of $\beta > 1$ would allow the insurer to sell her services at a mark-up (one could argue that this might be profitable for an insurer who has the appropriate infrastructure in place anyway for the rest of her portfolio). In our analysis, we stick to the interpretation that these *outside* service contracts are offered by third parties, i.e. service providers, and their price is externally given and higher than any within-insurance price (i.e. $\beta _o > 1$, see above).

19 This follows immediately as by assumption $\mathbb {E}_s[X] > 0$, $\beta _o - \beta > 0$, and $s \mapsto c(s)$ is non-negative and increasing, while by Lemma 1, $s \mapsto \mathbb {E}_s[X]$ is non-negative and non-increasing.

20 This can be seen even more clearly by rewriting Equation (A3) in terms of *elasticity* $\eta$ with respect to *s* [as used in economics for e.g. the price-elasticity of demand, see e.g. Parkin et al. (2002)], i.e. for $0< s_1< s_2 < \infty$ as

$$\begin{aligned} \underbrace{\frac{\frac{\mathbb {E}_{s_2}[X] - \mathbb {E}_{s_1}[X]}{\mathbb {E}_{s_1}[X]}}{\frac{s_2-s_1}{s_1}}}_{\eta _{\mathbb {E},s}} \le \underbrace{\frac{\frac{\rho _{s_2}[X] - \rho _{s_1}[X]}{\rho _{s_1}[X]}}{\frac{s_2-s_1}{s_1}}}_{\eta _{\rho ,s}} \le 0, \end{aligned}$$

yielding that the expectation is more elastic with respect to service than the risk measure.

21 As mentioned above, one could theoretically allow the insurer to offer “service-only” contracts by solving

6 $$\begin{aligned} \min _{\beta \in [1,\beta _o]} (1-\beta ) c(s_N(\beta )), \end{aligned}$$

which certainly yields a non-positive objective value. It might be feasible to assume that the insurer would be able to offer such services cheaper than other market participants, as she might have certain service infrastructures (contracts with IT experts, warning mechanisms) in place already for her insurance clients. One might also assume that the insurer has initially solved this problem, thus, determining $\beta _o$, and the upper bound in (6) is the next-cheapest outside option. Under no circumstance would we find it realistic to allow the insurer to simultaneously compare (negative) objective values of (5) and (6) and choose the lower one. In other words, the insurer should not compare for a prospective buyer where risk transfer is profitable whether it could be more profitable to offer only services and choose a solution that discourages the buyer from buying risk transfer.

22 The cited works typically use two processes, one to model the state of infectiousness among nodes in the graph and another one for loss occurrences among “infected” nodes; we regard this additional complexity as unnecessary for the present example.

23 Note that (9) implies that by buying service from the insurer, firm 2 can reduce the probability of a non-propagated loss only.

24 This example is somewhat related to the question studied in Khalili et al. (2019) about jointly underwriting a service provider and its customers as interdependent insurance customers.

25 Note that the terms corresponding to contract 2 are not adjusted at this point and therefore will not appear in the minimisation.

26 The calculation of the gradients, used in the numerical optimisation routine, is detailed in section A.7.1 of the electronic supplementary information.

27 One recent example were the multiple, sometimes effectively simultaneous attacks on exchange servers via the so-called *ProxyShell* exploit during 2021, see, e.g. Born (2021).

28 This corresponds to the seminal Marshall–Olkin shock model, see Marshall and Olkin (1967).

29 In this case $X_1$ and $X_2$ are comonotone iff $\lambda _1 = \lambda _2 = 0$, implying $y_{00} = e^{-\lambda _{12}},\; y_{10} = y_{01} = 0$ such that the risk measure is additive (a well-known general result for DRM): $\rho (X_1)+\rho (X_2) = \rho (X) = (L_1+L_2)(1-e^{-\lambda _{12}})^{r_0}$.

30 Our model implicitly equates incident arrival times (e.g. $Z_1 := \min \{E_1,E_{12}\}$) with loss occurrence times, which would not allow time for a warning mechanism as all losses occur instantly and simultaneously. In reality, however, the discovery and exploitation of the same vulnerability in different firms can be delayed over time, see again, e.g. the *ProxyShell* exploit case (Born 2021). As we do not take into account discounting over the policy year and therefore do not need to explicitly model a delayed loss occurrence time after the incident arrival time, we assume the warning mechanism to directly prevent the incident arrival.

31 In this sequential set-up, there is no distinction between *idiosyncratic* incidents and incidents from *systemic* events yet, as firm 1 is not yet part of the portfolio; in other words, the overall rate $\lambda _{II} = \lambda _2+\lambda _{12}$ can be observed, but it is not yet distinguished between $\lambda _{2}$ and $\lambda _{12}$.

32 Note that in this set-up, neither independence nor comonotonicity can be reached, as $b_{12} > 0$ and $\lambda _1 > 0$, respectively.

33 The gradients used for the numerical optimisation are given in section A.7.2 in the electronic supplementary information. Due to the symmetrical set-up of the dependence, we do not consider the reverse order of contract closures.

34 Note that contrary to the last example, the x-axis does not start at $\lambda _{12}(0)=0$ representing (initial) independence, resulting in $\beta _1^*<1$ for the whole depicted range $\lambda _{12}(0) \in \{0.15,2\}$.

35 Extreme-value copulas allow to capture the dependence structure between certain rare events, for details see, e.g. Mai and Scherer (2017). The necessity of dealing adequately with *extreme* events in the cyber context has been emphasised by many authors, e.g. the comprehensive data-driven analysis of cyber losses by Eling and Wirfs (2019) advocated for distinguishing between “cyber risks of daily life” and “extreme cyber risks”.

36 Note that generally, an Archimedean copula is not parametrised by a parameter $\theta$, but by the so-called (Archimedean) generator $\psi = \psi _\theta$, a non-increasing function $\psi : [0,\infty ) \rightarrow [0,1]$ with $\psi (0)=1$ and $\lim _{x \rightarrow \infty } \psi (x)=0$. The Gumbel copula is obtained by using the parametric family $\psi _\theta (x) = \exp \Big (-x^{\frac{1}{\theta }}\Big )$; for brevity, we use the notation $C_\theta$ instead of $C_{\psi _\theta }$.

37 An immediate generalization is a proportional insurance share $\alpha \in \{0\} \cup [\alpha _0,1]$, which could illustrate not only the two cases *no insurance* and *full insurance*, but additionally the case where the insurance buyer purchases a minimum feasible share of risk transfer $\alpha _0$ in order to benefit from the risk reduction services within insurance; in other words, insurers could sell policies that customers would not buy from a pure risk transfer viewpoint by including attractive services.
