# Supplementary material for: Risk mitigation services in cyber insurance: optimal contract design and price structure
Source: Geneva Pap Risk Insur Issues Pract. 2023 May 8;48(2):502–47. doi: 10.1057/s41288-023-00289-7 (PMC10165595; doi:10.1057/s41288-023-00289-7)
Supplement: Supplementary file 1 — Supplementary file (PDF 2902 KB) [file 41288_2023_289_MOESM1_ESM.pdf]

A Electronic Supplementary Information

This is the electronic supplement to the article: Zeller, G. and M. Scherer. 2023. *Risk mitigation services in cyber insurance: optimal contract design and price structure.*
It contains a seminal discussion of risk-assessment services, mathematical preliminaries, proofs, case studies, and extended calculations.

A.1 A Note on Risk-Assessment Services

An important, yet challenging, aspect of cyber insurance is risk assessment. In established insurance lines, insurers can rely on plenty of historical claims and in-house expertise to classify prospective policyholders into risk categories (e.g. using standardized models for mass segments such as motor insurance, individual expert judgement for large risks in industrial lines, or a mixture of both). In contrast, due to the novelty, non-stationarity, and complexity of cyber risk, the choice of relevant risk factors and the process of their assessment is a challenging task. For example, it seems intuitive that a policyholder's IT infrastructure and existing cyber security provisions are an important factor for his susceptibility to cyber risk. However, how to include extensive qualitative information about a system's structure and potential vulnerabilities into an actuarial model is a complex open issue in itself. Nevertheless, insurers often cooperate with specialized IT security experts to conduct extensive IT audits of a prospective policyholder before pricing insurance coverage (and choosing to make an offer at all). While the exact nature of these collaborations is of course opaque from the outside, it is likely that insurers currently carry the full costs of these audits and subsume them under operational or acquisition costs of their cyber insurance business. Thus, for such services, the following questions naturally arise: *Which amount of effort should an insurer optimally invest in risk assessment? Under which circumstances is it (feasible and) optimal for an insurer to shift part of the cost of risk assessment to the insurance buyer?* To elaborate on these questions, three relevant points should be mentioned:

- An extensive risk assessment benefits both parties: While the insurer increases her knowledge about the to-be-insured risk (allowing for more accurate pricing and risk management), the prospective insurance buyer also benefits from gaining expert knowledge about his IT security situation, often even including an ordered list of priorities to be addressed to ensure efficient spending of limited IT (security) budget. This is particularly beneficial for small to medium-size enterprises without sophisticated internal IT divisions, for whom the risk assessment as part of the insurance take-up process may be the first comprehensive analysis of the cyber-security level of their organization. While not every inquiry about insurance prices leads to the closure of a cyber-insurance contract, the process may serve as a wake-up call for the acquisition of (additional) risk reduction measures within or outside of an insurance policy.
- It is clear that due to the dynamics of the cyber threat landscape and the ongoing evolution of IT ecosystems, the risk assessment process should not be conducted only once, but periodically during the life (and at the renewal) of a cyber-insurance policy. From this perspective, one may argue that this is a service offering complementary to the ones discussed in the main body of this study. While *self-protection* and *self-insurance* services

aim to reduce the loss probability and loss severity respectively, i.e. re-shape the original loss distribution (previously denoted F_0) favourably, *risk assessment* services acknowledge that neither party has accurate knowledge of F_0 and therefore aim at better understanding it.

- In our view, the issue of *incomplete information* in the cyber context is not exactly akin to *asymmetric information* or *moral hazard*. By that we mean that in reality, it is not usually the case that an insurance buyer has better knowledge (in a meaningful, quantifiable way) about their own IT systems than the insurer and seeks to obscure this knowledge from the insurer, such that she has to use risk assessment to extract this hidden information. Rather, both parties jointly seek the expertise of an IT security provider to better understand the underlying risk and thus through the risk assessment process jointly gain improved (shared) knowledge. Apart from the insurance buyer's own lack of information, it has to be considered that as cyber risks are partly non-insurable (in particular regarding reputational risks) and cyber policies (as other lines) come with exclusions in case relevant information is wrongly stated or willfully omitted at policy closure, the insurance buyer has no incentive to take malicious advantage of asymmetric information.

There are, so far, very few mathematical papers concerned with cyber risk assessment services.[39] Below, we propose a general mathematical framework to describe the setting where none of the parties has full information, but both are able to learn more about the true underlying distribution through a (costly) risk audit. The further study of this setting is outside the scope of this manuscript but remains an interesting problem for future research.

0. As before, denote by F_0 the *true* (unknown) distribution of the risk X the buyer faces.
1. As the buyer approaches the insurer, both parties have (distinct) subjective views about the distribution of the risk X:

   $(Buyer : pre\ audit)$: $F_{0,B}$ depending on his own (incomplete) knowledge about his IT systems.

   $(Insurer : pre\ audit)$: $F_{0,I}$ depending on her standard models for cyber and publicly available information about the buyer.

   Note that while insurers routinely think in terms of risk and probability distributions, already assuming that a regular insurance buyer would have a certain distribution in mind is one step ahead of reality. In practice, most companies may consider scenarios and a setting of uncertainty rather than an already formalized context of risk.
2. If an IT audit is conducted, this causes some effort $a > 0$ (where different options w.r.t. extensiveness are usually available) with associated cost $c(a)$ (with the typical assumptions

[39] An exception is [Khalili et al., 2018], who study the role of pre-screening (i.e. risk assessment) in cyber insurance (again in a setting of *asymmetric* information, i.e. assuming the insurance buyer has perfect information). They study the influence of risk aversion (in an expected utility setting) and interdependence of insurance buyers under very specific assumptions on the loss distribution (normal distribution with parameters depending on the insurance buyer's effort), the effect of effort on the loss distribution, the pre-screening process (the outcome being the true effort plus standard Gaussian noise), and the interdependence between agents.

of increasingness and convexity). At the conclusion of the audit, an IT security expert shares their subjective opinion with all other parties:

$$(IT\ Expert:\ audit)\ :\ F^a_{0,A}\ \text{ depending on the extent of the audit } a > 0.$$

The more effort is invested in the audit, the closer the expert assessment approaches the *true* distribution, i.e.

$$0 < a_1 \le a_2 < \infty :\ \ \mathcal{D}\big(F^{a_1}_{0,A}, F_0\big) \ge \mathcal{D}\big(F^{a_2}_{0,A}, F_0\big) \ge 0, \tag{12}$$

where $\mathcal{D}$ denotes some measure of distance between probability distributions (e.g. the Wasserstein distance between the associated probability measures, see e.g. [Vaserstein, 1969]).

3. Both buyer and insurer update their original views by incorporating the result of the IT audit, i.e.

$$(Buyer : post\ audit)\ \ \hat{F}_{0,B}(a) := h_B(F_{0,B}, F^a_{0,A}) \overset{e.g.}{=} w_B F_{0,B} + (1-w_B)F^a_{0,A}, \quad w_B \in [0,1],$$
$$(Insurer : post\ audit)\ \ \hat{F}_{0,I}(a) := h_I(F_{0,I}, F^a_{0,A}) \overset{e.g.}{=} w_I F_{0,I} + (1-w_I)F^a_{0,A}, \quad w_I \in [0,1].$$

As an example, we have assumed that each party uses a convex combination (mixture) of their original view and the new information, with potentially different weights. In general, $h_B(\cdot)$ and $h_I(\cdot)$ could denote any functions mapping two distribution functions (*a priori* and *IT audit*) to a new distribution function (*a posteriori*).

4. As an initial step, one may formulate the insurer's loss function depending on the risk assessment effort as

$$(pre\ audit)\ \ \Big[\rho_{I,F_{0,I}}(X) - (1+\theta)\mathbb{E}_{F_{0,I}}[X]\Big]\mathbb{1}_{\{(1+\theta)\mathbb{E}_{F_{0,I}}[X]\le\rho_{B,F_{0,B}}(X)\}},$$
$$(post\ audit)\ \ \Big[\rho_{I,\hat{F}_{0,I}(a)}(X) - (1+\theta)\mathbb{E}_{\hat{F}_{0,I}(a)}[X]\Big]\mathbb{1}_{\{(1+\theta)\mathbb{E}_{\hat{F}_{0,I}(a)}[X]\le\rho_{B,\hat{F}_{0,B}(a)}(X)\}} + c(a),$$

where $\rho_{I,\cdot}, \rho_{B,\cdot}$ denote the risk measures of the risk X used by the insurer and buyer, respectively, given their subjective views about the distribution of X (*a priori* without audit and *a posteriori* with audit).

However, the above loss function does not yet encode the insurer's preference for a better estimation of the true distribution, i.e. the gain resulting from (12). Crucially, note that in this setting, we are in the framework of *precaution* and *uncertainty* as opposed to *prevention* and *risk* in the main body of the paper. An excellent summary of both concepts and the corresponding literature can be found in [Courbage et al., 2013]. As outlined therein, there are several approaches to formalizing the *effect of more information*; a classical result (without intertemporal dependence) in an expected utility setting is due to the seminal work of [Epstein, 1980] whose results have been used as the foundation for many following studies. Another (contested) approach to *precaution* is the idea of ambiguity (aversion), see [Klibanoff et al., 2005]. The choice and application of such an approach to the above problem is an interesting next step in a consecutive research project.

A.2 Mathematical Prerequisites

Definition 1 (Law-invariant, coherent risk measure (see [Artzner et al., 1999, Kusuoka, 2001])). *A risk measure $\rho : L^1(\Omega, \mathcal{F}, \mathbb{P}) \to \mathbb{R}$ is a coherent risk measure if it has the following properties:*

1. *Monotonicity:* $X \leq Y \implies \rho(X) \leq \rho(Y)$;
2. *Cash-additivity / translation invariance:* $\forall\, m \in \mathbb{R} : \ \rho(X - m) = \rho(X) - m$;
3. *Convexity:* $\forall\, X, Y \in L^1, \forall\, \lambda \in [0,1] : \ \rho(\lambda X + (1-\lambda)Y) \leq \lambda\rho(X) + (1-\lambda)\rho(Y)$;
4. *Positive homogeneity:* $\forall\, \lambda \in [0,\infty) : \ \rho(\lambda X) = \lambda\rho(X)$.

Note that under property 4., convexity is equivalent to sub-additivity. A coherent risk measure is called law-invariant if additionally:

5. *Law-invariance:* $X \overset{(d)}{=} Y \implies \rho(X) = \rho(Y)$, *where* $\overset{(d)}{=}$ *denotes equality in distribution.*

Definition 2 (Copula, see, e.g., [Mai and Scherer, 2017]). *A function $C : [0,1]^d \to [0,1]$ is called copula, if there is a random vector $(U_1, \ldots, U_d)$ such that $U_j \sim \mathcal{U}[0,1],\ j \in \{1, \ldots, d\}$, and C is the joint c.d.f. of $(U_1, \ldots, U_d)$, i.e.*

$$C(u_1, \ldots, u_d) = \mathbb{P}(U_1 \leq u_1, \ldots, U_d \leq u_d), \ \ u_1, \ldots, u_d \in [0,1].$$

Theorem 2 (Sklar's Theorem, see [Sklar, 1959]). *A function $F : \mathbb{R}^d \to [0,1]$ is the c.d.f. of a random vector $(X_1, \ldots, X_d)$ iff there exist a copula $C : [0,1]^d \to [0,1]$ and univariate c.d.f.s $F_1, \ldots, F_d : \mathbb{R} \to [0,1]$ s.t.*

$$C\big(F_1(x_1), \ldots, F_d(x_d)\big) = F(x_1, \ldots, x_d), \ \ \forall x_1, \ldots, x_d \in \mathbb{R}.$$

A.3 Proofs and Derivations of Sections 2 and 3

The following lemma details the relationship between the parameter s and the distortion risk measure $\rho_s(X) := \int_0^\infty \psi(\overline{F}_{X,s}(x))\mathrm{d}x$ under the assumptions of Section 2.

Lemma 1 ([Bensalem et al., 2020], Lemma 2.1). *Let ρ be a coherent, law-invariant risk measure and assume X to be distributed according to a family of distributions F_s such that* (A2) *holds. Then, the map $s \mapsto \rho_s(X)$ is convex, continuous, non-increasing and $\rho_s(X) \geq \mathbb{E}_s[X] > 0$.*

Proof. *See proof of Lemma 2.1 in [Bensalem et al., 2020].*

Choosing $\psi(u) = u$, the statements of Lemma 1 carry over to the special case $s \mapsto \mathbb{E}_s[X]$. For notational convenience, we formalize the following:

Assumption 3 (Monotonicity of $s \mapsto \frac{\rho_s(X)}{\mathbb{E}_s[X]}$). *Assume that $s \mapsto \frac{\rho_s(X)}{\mathbb{E}_s[X]}$ is monotone non-decreasing, i.e.*

$$\forall\, 0 \leq s_1 \leq s_2 < \infty : \ \frac{\rho_{s_2}(X)}{\mathbb{E}_{s_2}[X]} \geq \frac{\rho_{s_1}(X)}{\mathbb{E}_{s_1}[X]} \iff 0 \leq \frac{\rho_{s_1}[X] - \rho_{s_2}[X]}{\rho_{s_1}[X]} \leq \frac{\mathbb{E}_{s_1}[X] - \mathbb{E}_{s_2}[X]}{\mathbb{E}_{s_1}[X]}. \tag{A3}$$

Lemma 2 (A sufficient condition for Assumption 3 - [Bensalem et al., 2020], Lemma 3.2)**.** *Let ρ be a distortion risk measure with concave distortion function ψ and let the distribution of X be as in* (4)*. Then, the map $s \mapsto \frac{\rho_s(X)}{\mathbb{E}_s[X]}$ is non-decreasing.*

An alternative proof to the one given in [Bensalem et al., 2020] is given below.

Proof (Lemma 2)**.** *To show* (A3)*, rearranging the equation and using that by Equation* (4) *the survival functions $\overline{F}_{X,s}$ are given by*

$$\overline{F}_{X,s}(x) = \begin{cases} 1 & \text{if } x < 0, \\ p(s)\overline{F}_Y(x) & \text{if } x \geq 0, \end{cases}$$

yield the need to show for $s_2 \geq s_1$

$$\rho_{s_2}(X) \geq \rho_{s_1}(X)\frac{\mathbb{E}_{s_2}[X]}{\mathbb{E}_{s_1}[X]} = \rho_{s_1}(X)\frac{p(s_2)\mathbb{E}[Y]}{p(s_1)\mathbb{E}[Y]} = \rho_{s_1}(X)\frac{p(s_2)}{p(s_1)}.$$

Note that for the concave function ψ with $\psi(0) = 0$ it holds

$$\forall t \in [0,1],\ x \in \mathbb{R}:\ \psi(tx) \geq t\psi(x), \tag{13}$$

implying

$$\begin{aligned} \rho_{s_1}(X)\frac{p(s_2)}{p(s_1)} &= \int_0^\infty \underbrace{\frac{p(s_2)}{p(s_1)}}_{\in[0,1]} \psi\Big(p(s_1)\overline{F}_Y(x)\Big)\mathrm{d}x \overset{(13)}{\leq} \int_0^\infty \psi\Big(\frac{p(s_2)}{p(s_1)}p(s_1)\overline{F}_Y(x)\Big)\mathrm{d}x \\ &= \int_0^\infty \psi\Big(p(s_2)\overline{F}_Y(x)\Big)\mathrm{d}x = \rho_{s_2}(X). \end{aligned}$$

Lemma 3 ([Bensalem et al., 2020], Lemma 3.3)**.** *Assume that ρ is a distortion risk measure with a distortion function ψ such that $s \mapsto \psi(p(s))$ is convex. Then $s \mapsto \rho_s(X)$ and $s \mapsto \mathbb{E}_s[X]$ are convex.*

Proof (Lemma 3)**.** *See proof of Lemma 3.3 in [Bensalem et al., 2020].*

Proof (Corollary 1)**.** *The proof follows the derivation in [Bensalem et al., 2020], p.375 - where $s_B(\theta,\beta)$ for fixed $\beta \in [\underline{\beta}, 1]$ corresponds to e_θ therein - with the following amendments:*
1. The introduction of the constant θ_0 allows the inclusion of the values $s = 0$ and $s = s_B(\theta,\beta)$ in either of the sets $\mathcal{I}$ or $\mathcal{N}$, and the statement of increasingness (instead of non-decreasingness) in the case $\theta > \theta_0$.
2. To derive the definition of $s_B(\theta,\beta)$ and show its increasingness in θ, we use the map $G^\beta(s)$ with given assumptions on $s \mapsto c(s)$ (increasing and continuous with $c(0) = 0$ and $\lim_{s\to\infty} c(s) = \infty$).
3. We need to show increasingness of $\beta \mapsto s_B(\theta,\beta)$:
Note that for any $s > 0$, $\beta \mapsto G^\beta(s)$ is decreasing: Let $\underline{\beta} \leq \beta_1 < \beta_2 \leq 1$, then

$$G^{\beta_1}(s) = \frac{\rho_{1,s}(X)}{\mathbb{E}_s[X]} + \underbrace{(\beta_o - \beta_1)}_{>0}\underbrace{\frac{c(s)}{\mathbb{E}_s[X]}}_{>0} > \frac{\rho_{1,s}(X)}{\mathbb{E}_s[X]} + (\beta_o - \beta_2)\frac{c(s)}{\mathbb{E}_s[X]} = G^{\beta_2}(s).$$

Therefore, $s_B(\theta,\beta)$ is increasing in β:

$$s_B(\theta,\beta_1) = \min\{s > 0 : G^{\beta_1}(s) \geq 1+\theta\} < \min\{s > 0 : G^{\beta_2}(s) \geq 1+\theta\} = s_B(\theta,\beta_2).$$

Proof (Corollary 2)**.**
Part 1.:
First of all, assume that $\theta > \theta_0$, else $\mathcal{N}$ is empty and a local minimizer on this set does not exist. Following the proof of Proposition 3.4 in [Bensalem et al., 2020], we define for fixed β the (finite) constant

$$\theta_N(\beta) := \inf\{\theta \geq 0 : s_B(\theta, \beta) > s_N\}$$

*to derive the statement, where we note that in the case $\theta < \theta_N(\beta)$, the argument "the concave map $L_{\mathcal{N}}$ is non-decreasing on $\mathcal{N}$" has to be replaced by "the **convex** map $L_{1,\mathcal{N}}$ is non-**in**creasing on $\mathcal{N}$".*
Furthermore, we note that as $\beta \mapsto s_B(\theta, \beta)$ is increasing by Lemma 1 and s_N does not depend on β, by definition $\beta \mapsto \theta_N(\beta)$ is decreasing.
Part 2.:
The definition of the constant $\theta_I(\beta)$ for any fixed $\beta \in [\underline{\beta}, 1]$ and the increasingness of $\theta \mapsto s_I(\theta, \beta)$ follow the first part of the proof of Proposition 3.2 in [Bensalem et al., 2020], whereby the loss function $L^{\theta,\beta}_{1,\mathcal{I}}$ is considered. We additionally have to show decreasingness of $\beta \mapsto s_I(\theta, \beta)$ in case $\theta > \theta_I(\beta)$. Here, the global minimizer $s_I(\theta, \beta)$ is an interior point on $(0, \infty)$ characterized by

$$[L^{\theta,\beta}_{1,\mathcal{I}}(s)]'|_{s=s_I(\theta,\beta)} = 0 \iff \beta = \underbrace{\frac{1}{c'(s)|_{s=s_I(\theta,\beta)}}}_{>0,\ \textit{decreasing in } s} \underbrace{(-(1+\theta)\mathbb{E}'_s[X]|_{s=s_I(\theta,\beta)})}_{>0,\ \textit{non-increasing in } s}. \tag{14}$$

As the left-hand side is increasing in β, so must be the right-hand side. As $s \mapsto \frac{1}{c'(s)}(-(1+\theta)\mathbb{E}'_s[X])$ is non-increasing as a product of two positive, (at least) non-increasing functions, the inner function $\beta \mapsto s_I(\theta, \beta)$ must be decreasing.

To prove Corollary 3, we introduce the following lemma, where for the rest of this section, we suppress the dependencies $s_I(\theta, \beta)$ and $s_B(\theta, \beta)$ for brevity.

Lemma 4 (Adapted from [Bensalem et al., 2020])**.** *If*

$$(N1)\ \ s_N < s_I < s_B \qquad \textit{or} \qquad (N2)\ \ s_I \leq s_N < s_B,$$

the global minimizer of $L_1(\alpha^*(s), s)$ is $(\alpha^*, s^*) = (0, s_N)$.
If

$$(I1)\ \ s_B \leq s_I < s_N \qquad \textit{or} \qquad (I2)\ \ s_B \leq s_N \leq s_I,$$

the global minimizer of $L_1(\alpha^*(s), s)$ is $(\alpha^*, s^*) = (1, s_I)$.
The case $s_I < s_B \leq s_N$ is not possible, i.e. the global minimizers of $L^{\theta,\beta}_{1,\mathcal{I}}(s)$ resp. $L_{1,\mathcal{N}}(s)$ cannot be simultaneously outside of $\mathcal{I}$ resp. $\mathcal{N}$.
If

$$(T)\ s_N < s_B \leq s_I,$$

for any $\beta \in [\underline{\beta}, 1]$, there exists a constant $\theta_R(\beta) \geq 0$ such that

(i) If $\theta \leq \theta_R(\beta)$, the global minimizer of $L_1(\alpha^*(s), s)$ is $(\alpha^*, s^*) = (1, s_I)$.

(ii) If $\theta > \theta_R(\beta)$, the global minimizer of $L_1(\alpha^*(s), s)$ is $(\alpha^*, s^*) = (0, s_N)$.

Furthermore, it holds $\theta_R(\beta) \geq \theta_N(\beta)$.

Proof (Lemma 4). *See proof of Proposition 3.6 (for cases* $(N1)$ *and* $(N2)$*), Proposition 3.5/Corollary 3.2 (for cases* $(I1)$ *and* $(I2)$ *and the argument directly after), and Theorem 3.2 (for case* (T)*) in [Bensalem et al., 2020].*

Proof (Corollary 3). *The statement follows by combining the results of Lemma 4 by considering the progression through the cases for* $\theta \in [0, \infty)$*.*

- $\theta = 0$ *implies* $s_B = 0$, $\mathcal{I} = [0, \infty)$*, thus at* $\theta = 0$*, either* $(I1)$ *or* $(I2)$ *holds.*
- *If at* $\theta = 0$, $(I1)$ *holds, a transition to* $(I2)$ *must occur as* θ *increases (as so do* s_I *and* s_B*), as the only alternative* $s_I < s_B \leq s_N$ *is impossible (see Lemma 4). As* s_I *is increasing in* θ*, transitioning back is not possible.*
- $(I1)$ *or* $(I2)$ *occur exactly while* $s_B \leq s_N$ *(meaning* $s_N \notin \mathcal{N}$*), i.e. for* $0 \leq \theta \leq \theta_N(\beta)$ *(see Corollary 2). The optimal solution is* $(\alpha^*, s^*) = (1, s_I)$*.*
- *After crossing* $\theta = \theta_N(\beta)$*, one transitions from* $s_B \leq s_N$ *in* $(I2)$ *to one of the cases with* $s_N < s_B$*, i.e.* (T) *or* $(N1)$*. Note that* $(N1)$ *cannot occur while* $\theta \leq \theta_M(\beta)$*, as by definition of* $\theta_M(\beta)$ *and global optimality of* s_N *for* $L_{1,\mathcal{N}}$*, for any* $\theta \leq \theta_M(\beta)$ *it holds that* $s_I \in \mathcal{I} = [s_B, \infty)$ *as*
$$L^{\theta,\beta}_{1,\mathcal{I}}(s_I) \leq L_{1,\mathcal{N}}(s_N) < L_{1,\mathcal{N}}(s_I).$$
- *This implies that if the transition at* $\theta_N(\beta)$ *is to* $(N1)$*, then* $\theta_M(\beta) = \theta_N(\beta)$ *and if the transition is to* (T)*, then* $\theta_M(\beta) > \theta_N(\beta)$*. In any case, for* $\theta \leq \theta_M(\beta)$*, implication (i) of Lemma 4 holds, i.e.* $(\alpha^*, s^*) = (1, s_I)$*. For* $\theta > \theta_M(\beta)$*, either implication (ii) of Lemma 4 or Lemma* $(N1)$ *holds; in either case,* $(\alpha^*, s^*) = (0, s_N)$*.*

The assertion that $\theta_R(\beta) \geq \theta_N(\beta)$ *follows from Lemma 4.*
It remains to show that $\beta \mapsto \theta_R(\beta)$ *is non-increasing. Note that for any* $\theta \geq 0$*, the map* $\beta \mapsto L^{\theta,\beta}_{1,\mathcal{I}}(s_I)$ *is non-decreasing:*
Let $\underline{\beta} \leq \beta_1 < \beta_2 \leq 1$*, then*

$$\begin{aligned} L^{\theta,\beta_1}_{1,\mathcal{I}}\big(s_I(\theta,\beta_1)\big) &= (1+\theta)\mathbb{E}_{s_I(\theta,\beta_1)}[X] + \beta_1 c\big(s_I(\theta,\beta_1)\big) \\ &\leq (1+\theta)\mathbb{E}_{s_I(\theta,\beta_2)}[X] + \beta_1 c\big(s_I(\theta,\beta_2)\big) \\ &\leq (1+\theta)\mathbb{E}_{s_I(\theta,\beta_2)}[X] + \beta_2 c\big(s_I(\theta,\beta_2)\big) = L^{\theta,\beta_2}_{1,\mathcal{I}}\big(s_I(\theta,\beta_2)\big), \end{aligned}$$

where the first inequality stems from the global optimality of $s_I(\theta,\beta_1)$ *for* $L^{\theta,\beta_1}_{1,\mathcal{I}}$ *and both inequalities are strict unless* $s_I(\theta,\beta_1) = s_I(\theta,\beta_2) = 0$*.*
This implies that $\beta \mapsto L^{\theta,\beta}_{1,\mathcal{I}}(s_I)$ *is constant for* $\theta \leq \theta_I(\beta)$ *and increasing for* $\theta > \theta_I(\beta)$*, thus non-decreasing. It follows from the definition of* $\theta_R(\beta)$ *that*

$$\begin{aligned} \theta_R(\beta_1) &= \sup\{\theta \geq 0 : L^{\theta,\beta_1}_{1,\mathcal{I}}(s_I(\theta,\beta_1)) \leq L_{1,\mathcal{N}}(s_N)\} \\ &\geq \sup\{\theta \geq 0 : L^{\theta,\beta_2}_{1,\mathcal{I}}(s_I(\theta,\beta_2)) \leq L_{1,\mathcal{N}}(s_N)\} = \theta_R(\beta_2). \end{aligned}$$

Proof (Corollary 4). *See the proof of Corollary 3.3 in [Bensalem et al., 2020].*

A.4 Proofs of Section 4

Note that as above the derivative w.r.t. s will be denoted by the prime $()'$ and partial derivatives w.r.t. θ and β will be denoted explicitly as $\frac{\partial}{\partial\theta}$ and $\frac{\partial}{\partial\beta}$; occasionally, the dependency $s_I(\theta,\beta)$ is omitted for brevity. Furthermore, we assume all derivatives calculated in the following to exist.

Proof (Proposition 1). *The partial derivative of the insurer's loss w.r.t. β is given by*

$$\frac{\partial}{\partial\beta}L_0(\theta,\beta) = \underbrace{\frac{\partial}{\partial\beta}s_I(\theta,\beta)}_{\leq 0 \text{ by Cor. 2}}\Big[\underbrace{\rho'_{0,s}(X)|_{s=s_I} - (1+\theta)\mathbb{E}'_s[X]|_{s=s_I}}_{(*)} + \underbrace{(1-\beta)c'(s)|_{s=s_I}}_{\geq 0 \text{ by assumption}}\Big]\underbrace{-c(s_I(\theta,\beta))}_{\leq 0}$$

Showing $(*) > 0$ directly implies $\frac{\partial}{\partial\beta}L_0(\theta,\beta) \leq 0$ such that the claim follows. Recall that a necessary condition for the insurer to be willing to offer a contract is that it entails a negative loss, i.e.

$$L_0(\theta,\beta) = \rho_{0,s_I(\theta,\beta)}(X) - (1+\theta)\mathbb{E}_{s_I(\theta,\beta)}[X] + (1-\beta)c(s_I(\theta,\beta)) < 0.$$

As the last term is non-negative, this implies the necessity of

$$\rho_{0,s_I(\theta,\beta)}(X) < (1+\theta)\mathbb{E}_{s_I(\theta,\beta)}[X], \tag{15}$$

i.e. that the insurer's measure of the risk taken over must be at least compensated by the premium received (and unless $\beta = 1$, the difference must furthermore compensate the additional cost taken over by the provision of services). Recall that for a risk-averse insurer, Lemma 2 states that for any $s \geq 0$

$$0 \geq \mathbb{E}_s[X]\rho'_s(X) \geq \rho_s(X)\mathbb{E}'_s[X] \iff 0 \leq \mathbb{E}_s[X](-\rho'_s(X)) \leq \rho_s(X)(-\mathbb{E}'_s[X]),$$

which, together with Equation (15), *implies that in particular also at $s = s_I$,*

$$0 \leq \mathbb{E}_{s_I}[X](-\rho'_{0,s_I}(X)) \leq \rho_{0,s_I}(X)(-\mathbb{E}'_{s_I}[X]) \overset{(15)}{<} (1+\theta)\mathbb{E}_{s_I}[X](-\mathbb{E}'_{s_I}[X]).$$

Dividing by $\mathbb{E}_{s_I}[X] > 0$ and rearranging yield

$$0 \geq \rho'_{0,s_I}(X) > (1+\theta)\mathbb{E}'_{s_I}[X] \iff (*) = \rho'_{0,s_I}(X) - (1+\theta)\mathbb{E}'_{s_I}[X] > 0. \tag{16}$$

The claim holds with strict inequality (i.e. $\frac{\partial}{\partial\beta}L_0(\theta,\beta) < 0$) unless $s_I(\theta,\beta) = 0$.

Proof (Proposition 2). *Recall that by definition, for any $\beta \in [\underline{\beta}, 1]$*

$$\theta_R(\beta) = \sup\left\{\theta \geq 0 : L^{\theta,\beta}_{1,\mathcal{I}}(s_I(\theta,\beta)) \leq L_{1,\mathcal{N}}(s_N)\right\}$$

is the highest loading the insurance buyer would accept. As $\theta \mapsto L^{\theta,\beta}_{1,\mathcal{I}}(s_I(\theta,\beta))$ is increasing with $\lim_{\theta\to\infty} L^{\theta,\beta}_{1,\mathcal{I}}(s_I(\theta,\beta)) = \infty$, the supremum is attained and by continuity of $L^{\theta,\beta}_{1,\mathcal{I}}$ it holds that at $(\theta_R(\beta),\beta)$ the insurance buyer is indifferent between buying and not buying insurance, i.e.

$$\begin{aligned} L^{\theta_R(\beta),\beta}_{1,\mathcal{I}}(s_I(\theta_R(\beta),\beta)) &= L_{\mathcal{N}}(s_N) \\ (1+\theta_R(\beta))\,\mathbb{E}_{s_I(\theta_R(\beta),\beta)}[X] + \beta c(s_I(\theta_R(\beta),\beta)) &= \rho_{1,s_N}(X) + \beta_o c(s_N) \\ \iff (1+\theta_R(\beta))\,\mathbb{E}_{s_I(\theta_R(\beta),\beta)}[X] &= \rho_{1,s_N}(X) + \beta_o c(s_N) - \beta c(s_I(\theta_R(\beta),\beta)). \end{aligned} \tag{17}$$

Thus, the insurer's loss function on the boundary $(\theta_R(\beta),\beta)$ *is given by*

$$\begin{aligned} L_0(\theta_R(\beta),\beta) &= \rho_{0,s_I(\theta_R(\beta),\beta)}(X) - (1+\theta_R(\beta))\mathbb{E}_{s_I(\theta_R(\beta),\beta)}[X] + (1-\beta)c(s_I(\theta_R(\beta),\beta)) \\ &\overset{(17)}{=} \rho_{0,s_I(\theta_R(\beta),\beta)}(X) + c(s_I(\theta_R(\beta),\beta)) - \rho_{1,s_N}(X) - \beta_o c(s_N) < 0. \end{aligned}$$

The total derivative of $L_0(\theta_R(\beta),\beta)$ *w.r.t.* β *at* $s = s_I(\theta_R(\beta),\beta)$ *is given by*

$$\begin{aligned} \frac{\mathrm{d}}{\mathrm{d}\beta}L_0(\theta_R(\beta),\beta) &= \frac{\mathrm{d}}{\mathrm{d}\beta}\rho_{0,s_I(\theta_R(\beta),\beta)}(X) + \frac{\mathrm{d}}{\mathrm{d}\beta}c(s_I(\theta_R(\beta),\beta)) + \underbrace{\frac{\mathrm{d}}{\mathrm{d}\beta}\Big(-\rho_{1,s_N}(X) - \beta_o c(s_N)\Big)}_{=0} \\ &= \rho'_{0,s}(X)|_{s=s_I(\theta_R(\beta),\beta)}\left[\frac{\partial}{\partial\theta}s_I(\theta,\beta)|_{\theta=\theta_R(\beta)}\ \frac{\mathrm{d}}{\mathrm{d}\beta}\theta_R(\beta) + \frac{\partial}{\partial\beta}s_I(\theta,\beta)|_{\theta=\theta_R(\beta)}\right] \\ &\quad + c'(s)|_{s=s_I(\theta_R(\beta),\beta)}\Big[\frac{\partial}{\partial\theta}s_I(\theta,\beta)|_{\theta=\theta_R(\beta)}\ \frac{\mathrm{d}}{\mathrm{d}\beta}\theta_R(\beta) + \frac{\partial}{\partial\beta}s_I(\theta,\beta)|_{\theta=\theta_R(\beta)}\Big] \\ &= \Big[\underbrace{\rho'_{0,s}(X)|_{s=s_I}}_{\le 0} + \underbrace{c'(s)|_{s=s_I}}_{\ge 0}\Big]\underbrace{\Big[\underbrace{\frac{\partial}{\partial\theta}s_I(\theta,\beta)|_{\theta=\theta_R(\beta)}}_{\ge 0 \text{ by Cor. 2}}\ \underbrace{\frac{\mathrm{d}}{\mathrm{d}\beta}\theta_R(\beta)}_{\le 0 \text{ by Cor. 3}} + \underbrace{\frac{\partial}{\partial\beta}s_I(\theta,\beta)|_{\theta=\theta_R(\beta)}}_{\le 0 \text{ by Cor. 2}}\Big]}_{\le 0}. \end{aligned} \tag{18}$$

While it follows from the previous computations that the second factor is non-positive, the sign of the first factor is not yet determined, as the derivatives of the risk measure and the cost w.r.t. s *are of opposite signs. However, we show that their sum is always positive when evaluated at the optimal solution* $s_I(\theta_R(\beta),\beta)$ *of the buyer, such that it follows immediately that the overall product in* (18) *and thus the sign of* $\frac{\mathrm{d}}{\mathrm{d}\beta}L_0(\theta_R(\beta),\beta)$ *is non-positive.*
Recall from the proof of Corollary 2 that $s_I(\theta,\beta)$ *is the global minimizer of the strictly convex function* $L^{\theta,\beta}_{1,\mathcal{I}}(s)$ *and that for any* β *there exists* $\theta_I(\beta)$ *such that*

$$\begin{aligned} \theta \le \theta_I(\beta) &\implies [L^{\theta,\beta}_{1,\mathcal{I}}(s)]'|_{s=0} \ge 0,\ s_I(\theta,\beta) = 0, \\ \theta > \theta_I(\beta) &\implies [L^{\theta,\beta}_{1,\mathcal{I}}(s)]'|_{s=0} < 0,\ s_I(\theta,\beta) > 0,\ [L^{\theta,\beta}_{1,\mathcal{I}}(s)]'|_{s=s_I} = 0. \end{aligned}$$

In the first case, the loss function is non-decreasing at $s = 0$ *and therefore by convexity non-decreasing everywhere, thus the global minimizer given by* $s_I = 0$*. In the second case, the loss function is decreasing at* 0*, thus the global minimizer an interior point on* $(0,\infty)$ *characterized by the first-order optimality condition.*
In any case, the derivative of the loss function evaluated at the global minimizer is non-negative, i.e.

$$[L^{\theta_R(\beta),\beta}_{1,\mathcal{I}}(s)]'|_{s=s_I(\theta_R(\beta),\beta)} \ge 0. \tag{19}$$

Therefore, for the first factor in (18)*, it follows*

$$\begin{aligned} &\rho'_{0,s}(X)|_{s=s_I(\theta_R(\beta),\beta)} + c'(s)|_{s=s_I(\theta_R(\beta),\beta)} \overset{\beta\le 1}{\ge} \rho'_{0,s}(X)|_{s=s_I(\theta_R(\beta),\beta)} + \beta c'(s)|_{s=s_I(\theta_R(\beta),\beta)} \\ &\overset{(16)}{>} (1+\theta_R(\beta))\mathbb{E}'_s[X]|_{s=s_I(\theta_R(\beta),\beta)} + \beta c'(s)|_{s=s_I(\theta_R(\beta),\beta)} = [L^{\theta_R(\beta),\beta}_{1,\mathcal{I}}(s)]'|_{s=s_I(\theta_R(\beta),\beta)} \overset{(19)}{\ge} 0, \end{aligned}$$

implying the claim of the proposition and Theorem 1.
Note again that unless $\theta_R(\beta) < \theta_I(\beta)$ *implying* $s_I(\theta_R(\beta),\beta) = 0$*, the second factor of the derivative is negative (instead of non-positive), leading to uniqueness of the solution.*

A.5 Case Study: Self-Protection with a Pareto Loss

To illustrate the results for a single insurance buyer in a self-protection setting, we now consider a loss with c.d.f.

$$F_{X,s}(x) = (1 - p(s)) + p(s)F_Y(x),\ x \geq 0,$$

where $0 \leq p(s) \leq 1$ and F_Y is the c.d.f. of a Pareto-distributed r.v. $Y \sim \text{Pareto}(\hat{x}, k)$ (compare [Bensalem et al., 2020]), i.e. a *zero-inflated Pareto distribution.* Naturally, $s \mapsto p(s)$ is assumed to be decreasing (loss probability decreases as service increases) and convex (decreasing marginal impact). Additionally, assume that $\lim_{s\to\infty} p(s) > 0$, i.e. the risk of a loss can never be completely eliminated.

Assume that both parties use the PH transform, where the exponents of the distortion function express that the insurer (index r_0) is less risk averse than the buyer (index r_1): $\psi_1(u) = u^{r_1},\ \psi_0(u) = u^{r_0},\quad r_1, r_0 \in (0,1], r_0 > r_1$. Furthermore, assume for the cost of service (recall that $s \mapsto c(s)$ is assumed increasing and strictly convex with $c(0) = 0$) the functional form $c(s) = \eta s^\gamma,\ \eta > 0,\ \gamma > 1$. It follows (see [Bensalem et al., 2020]):

$$\bar{F}_{X,s}(x) = \mathbb{P}_s(X > x) = \begin{cases} p(s) & \forall\ 0 \leq x \leq \hat{x}, \\ p(s)\left(\frac{\hat{x}}{x}\right)^k, & \forall\ \hat{x} < x, \end{cases}$$

$$\mathbb{E}_s[X] = \begin{cases} p(s)\frac{\hat{x}k}{k-1}, & \text{if } k > 1, \\ \infty, & \text{else}, \end{cases} \qquad \rho_{1,s}(X) = \begin{cases} \frac{\hat{x}r_1kp(s)^{r_1}}{r_1k-1}, & \text{if } k > \frac{1}{r_1}, \\ \infty, & \text{else}, \end{cases}$$

such that from now on, we assume $k > \frac{1}{r_1}$. In this case, $G^\beta(s)$ is given by

$$G^\beta(s) = \frac{\rho_{1,s}(X)}{\mathbb{E}_s[X]} + (\beta_o - \beta)\frac{c(s)}{\mathbb{E}_s[X]} = \frac{r_1(k-1)}{r_1k-1}p(s)^{r_1-1} + (\beta_o - \beta)\frac{\eta s^\gamma(k-1)}{\hat{x}kp(s)},$$

which is indeed a non-decreasing function of s, as $s \mapsto p(s)$ is non-increasing and $r_1 - 1 < 0$. As $\theta_0 = \frac{r_1(k-1)p(0)^{r_1-1}}{r_1k-1} - 1$, it follows:

- If $\theta \leq \frac{r_1(k-1)p(0)^{r_1-1}}{r_1k-1} - 1$, this implies $\mathcal{N} = \emptyset$, $\mathcal{I} = [0, \infty)$;
- If $\theta > \frac{r_1(k-1)p(0)^{r_1-1}}{r_1k-1} - 1$, this implies $\mathcal{N} = [0, s_B(\theta,\beta))$, $\mathcal{I} = [s_B(\theta,\beta), \infty)$, where by continuity $s_B(\theta,\beta)$ solves

$$(1+\theta) - \frac{r_1(k-1)}{r_1k-1}p(s_B(\theta,\beta))^{r_1-1} - \frac{(\beta_o-\beta)(k-1)\eta}{\hat{x}k}s_B(\theta,\beta)^\gamma p(s_B(\theta,\beta))^{-1} = 0.$$

For $\theta > \theta_0$, recall that on $\mathcal{N}$, the insurance buyer minimizes

$$L_{1,\mathcal{N}}(s) = \rho_{1,s}(X) + \beta_o c(s) = \frac{\hat{x}r_1kp(s)^{r_1}}{r_1k-1} + \beta_o\eta s^\gamma.$$

From now on, let us assume that $p(s) = \frac{1}{a+s} + b,\ a \geq 1,\ b > 0$ (such that $p(0) \leq 1$), which ensures convexity of the buyer's problem.[40]

[40] Here, $s \mapsto L_{1,\mathcal{N}}(s)$ is convex iff $s \mapsto p(s)^{r_1}$ is convex. This is ensured if $(r_1-1)(p'(s))^2 + p(s)p''(s) \geq 0$, which is fulfilled for logarithmic convexity of $p(s)$ (see, e.g. [Niculescu and Persson, 2018]).

The optimality criterion for the global minimizer s_N of $L_{1,\mathcal{N}}(s)$ is then given by

$$L'_{1,\mathcal{N}}(s)|_{s=s_N} \stackrel{!}{=} 0 \iff s_N = \Big[-\frac{\hat{x}kr_1^2}{\beta_o\eta\gamma(r_1k-1)}p(s_N)^{r_1-1}p'(s_N)\Big]^{\frac{1}{\gamma-1}},$$

which is an interior point as $L'_{1,\mathcal{N}}(s)|_{s=0^+} < 0$ and $\lim_{s\to\infty} L'_{1,\mathcal{N}}(s) = \infty$.
Analogously, on $\mathcal{I}$, the buyer minimizes

$$L^{\theta,\beta}_{1,\mathcal{I}}(s) = (1+\theta)\mathbb{E}_s[X] + \beta c(s) = (1+\theta)\frac{\hat{x}k}{k-1}p(s) + \beta\eta s^\gamma,$$

whose derivative w.r.t. s is given by $[L^{\theta,\beta}_{1,\mathcal{I}}(s)]' = (1+\theta)\frac{\hat{x}k}{k-1}p'(s)+\beta\eta\gamma s^{\gamma-1}$, implying $[L^{\theta,\beta}_{1,\mathcal{I}}(s)]'|_{s=0} < 0 \ \forall\ \theta > 0$ such that in all cases, $s_I(\theta,\beta) > 0$ is an interior point and the results of Corollary 2 and Section 4 hold with strict inequality, in particular the insurer's solution is unique.
The optimality criterion for $s_I(\theta,\beta)$ is given by

$$[L^{\theta,\beta}_{1,\mathcal{I}}(s)]'|_{s=s_I} \stackrel{!}{=} 0 \iff s_I(\theta,\beta) = \Big[-\frac{(1+\theta)\hat{x}k}{\beta\eta\gamma(k-1)}p'(s_I(\theta,\beta))\Big]^{\frac{1}{\gamma-1}}, \qquad (20)$$

implying that $\theta \mapsto s_I(\theta,\beta)$ ($\beta \mapsto s_I(\theta,\beta)$) is increasing (decreasing) (Corollary 2).
The insurer's problem (5) in this setting becomes

$$\min_{(\theta,\beta)\in[0,\theta_R(\beta)]\times[\underline{\beta},1]} \frac{\hat{x}r_0k}{r_0k-1}\Big(\frac{1}{s_I(\theta,\beta)+a}+b\Big)^{r_0} - (1+\theta)\frac{\hat{x}k}{k-1}\Big(\frac{1}{s_I(\theta,\beta)+a}+b\Big) + (1-\beta)\eta s_I(\theta,\beta)^\gamma,$$

where $s_I(\theta,\beta)$ is characterized by Equation (20). Applying the results of Section 4 yields that the insurer's optimal solution lies in the set $\{(\theta,1),\ \theta\in[0,\underline{\theta}]\}$, i.e. the insurer's problem simplifies to

$$\min_{\theta\in[0,\theta_R(1)]} L_0(\theta,1) = \frac{\hat{x}r_0k}{r_0k-1}\Big(\frac{1}{s_I(\theta,1)+a}+b\Big)^{r_0} - (1+\theta)\frac{\hat{x}k}{k-1}\Big(\frac{1}{s_I(\theta,1)+a}+b\Big),$$

which can be shown to be monotone in θ (see [Bensalem et al., 2020]). We illustrate the buyer's and insurer's solution in Figures 6 and 7, respectively, for one exemplary set of parameters.[41]

- For small θ ($0\le\theta\le\theta_N$), i.e. the left region in Panel 6a where $s_B(\theta,1) < \min\{s_N,\ s_I(\theta,1)\}$, both s_N and $s_I(\theta,1)$ lie in $[s_B(\theta,1),\infty) = \mathcal{I}$. Thus, the solution for $L_{1,\mathcal{N}}$ is $s_B(\theta,1)$ (Corollary 2), while the problem for $L_{1,\mathcal{I}}$ has an interior solution on $\mathcal{I}$, which is also the global solution.
- For large θ, i.e. the right region in Panel 6a where $s_B(\theta,1) > s_I(\theta,1) > s_N$, both s_N and $s_I(\theta,1)$ lie in $[0, s_B(\theta,1)) = \mathcal{N}$, yielding s_N as global solution.
- The region where θ is such that $s_N < s_B(\theta,1) < s_I(\theta,1)$ is where both problems have interior solutions on their domains, thus to determine the global solution, it remains to compare the objective functions to find the boundary $\theta_R(1)$ (see Panel 6b).[42]

[41] The parameters in this example are: for the risk measures $r_1 = 0.5,\ r_0 = 0.6$ ($r_1 < r_0$ required), for the Pareto distr. $\hat{x} = 1,\ k = 2.5$ ($k > 1/r_1$ required), for the cost $\eta = 0.5,\ \gamma = 2,\ \beta_o = 1.2,\ \underline{\beta} = 0.05$, and for the loss probability $p(s) = \frac{1}{s+1/0.6} + 0.2$ implying $p(s) \in [\lim_{s\to\infty} p(s), p(0)] = [0.2, 0.8]$.

[42] One could compare the results in Figure 6 for different choices of β (which we know are not optimal to offer from the insurer's viewpoint): As expected, if β decreases, $s_I(\theta,\beta)$ increases for any θ (Corollary 2) and thus the size of the jump in s^* at $\theta_R(\beta)$ increases. Furthermore, as β decreases, $\theta_R(\beta)$ increases (Corollary 3).

- Note again that the optimal service demand within insurance increases with the loading, but jumps downward once the premium is too high for the contract to be acceptable – in other words, insurance and risk reduction are complements (Corollary 4).
- Panel 7a compares the maximum acceptable loading for the buyer (which as stated in Corollary 3 is non-increasing in $\beta \in [\underline{\beta}, 1]$) with the minimum acceptable loading for the insurer. For small β, i.e. in the gray region left of the vertical line, no mutually acceptable contract exists.
- This is also visible in Panel 7b depicting the insurer's loss for all parameter combinations in $\{(\theta_R(\beta), \beta),\ \beta \in [\underline{\beta}, 1]\} \cup \{(\theta, 1),\ \theta \in [0, \underline{\theta}]\}$, truncated at 0. The left of the dashed vertical line corresponds to $L_0(\theta_R(\beta), \beta),\ \beta \in [\underline{\beta}, 1]$. As stated in Proposition 2, $\beta \mapsto L_0(\theta_R(\beta), \beta)$ is decreasing, leaving the optimal solution to lie in $\{(\theta, 1),\ \theta \in [0, \underline{\theta}]\}$. The loss on this part of the boundary is depicted to the right of the vertical dashed line, and for the special case of a zero-inflated Pareto distribution is decreasing (see [Bensalem et al., 2020]), such that the unique optimal solution of the insurer's problem is $(\theta^*, \beta^*) = (\theta_R(1), 1) \approx (2.88, 1)$.
- The gray areas in Panel 7b where no negative insurer's loss can be attained correspond to the small values of β to the left of the vertical line on the first part of the boundary in Panel 7a (left gray area), and the small values $\theta \in [0, \theta_{\min}(1)]$ below the intersection of $\theta_{\min}(\beta)$ with the vertical part of the boundary (right gray area).

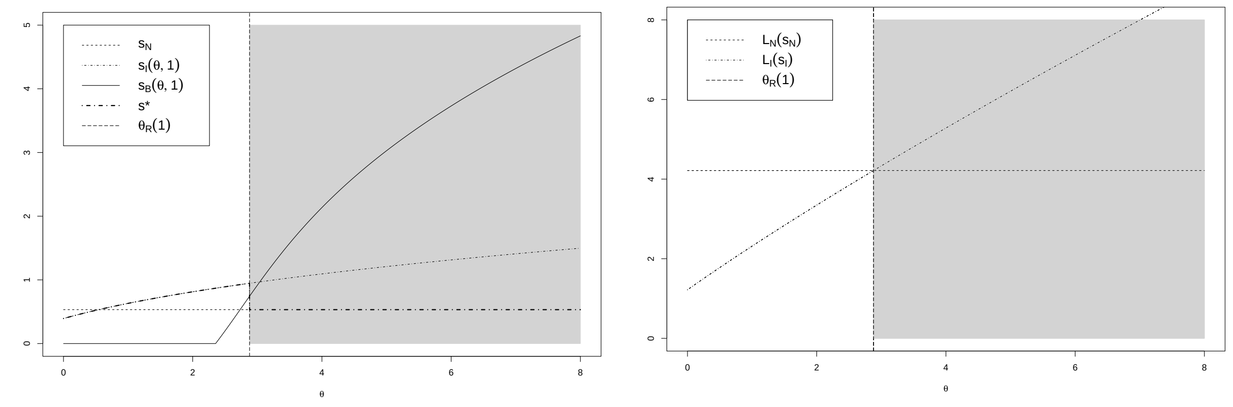

(a) s_N, $s_I(\theta, 1)$ and $s_B(\theta, 1)$.

(b) $L_{1,\mathcal{N}}(s_N)$ and $L_{1,\mathcal{I}}^{\theta,1}(s_I(\theta, 1))$.

Figure 6: Insurance buyer's solution depending on the loading θ, if he bears the full service cost ($\beta = 1$). Gray areas mark values of θ where no mutually acceptable contract exists.

A.6 Case Study: Self-Insurance with a Pareto Loss

Consider a loss with the following *zero-inflated Pareto* distribution:

$$F_{X,s}(x) = (1 - p) + pF_{Y,s}(x),\ x \geq 0,$$

where $0 < p < 1$ and $F_{Y,s}$ is the c.d.f. of a Pareto-distributed r.v. $Y \sim \text{Pareto}(\hat{x}, k(s))$, i.e. $\bar{F}_{Y,s}(x) = \left(\frac{\hat{x}}{x}\right)^{k(s)}$ for all $x > \hat{x}$ (see [Bensalem et al., 2020]).

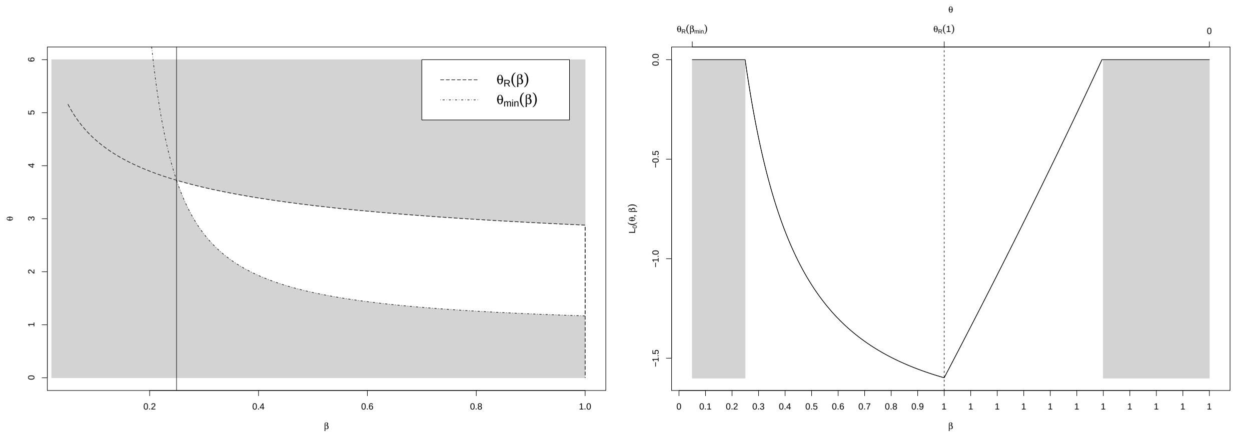

(a) $\theta_R(\beta)$ and $\theta_{\min}(\beta)$.

(b) $L_0(\theta_R(\beta), \beta),\ \beta \in [\underline{\beta}, 1]$ and $L_0(\theta, 1),\ \theta \in [0, \underline{\theta} = \theta_R(1)]$.

Figure 7: Insurer's solution: Comparison $\theta_R(\beta)$ (maximum acceptable loading for buyer) and $\theta_{\min}(\beta)$ (minimum acceptable loading for insurer) (left panel), and insurer's loss on the boundary $\{(\theta_R(\beta), \beta),\ \beta \in [\underline{\beta}, 1]\} \cup \{(\theta, 1),\ \theta \in [0, \underline{\theta}]\}$ (right panel). Areas where no mutually acceptable contract exists are marked gray.

This means that the service level controls the loss size via the map $s \mapsto k(s)$, which is assumed non-decreasing (service decreases loss severity), concave (decreasing marginal impact), and such that $k(0) =: z > \frac{1}{r_1} > 1$ (to ensure finiteness of $\rho_{1,s}(X)$ for any $s \geq 0$). As previously, assume $\psi_1(u) = u^{r_1},\ \psi_0(u) = u^{r_0},\ r_1, r_0 \in (0, 1], r_0 > r_1$, and $c(s) = \eta s^\gamma,\ \eta > 0,\ \gamma > 1$.[43] It follows (see [Bensalem et al., 2020]):

$$\bar{F}_{X,s}(x) = \begin{cases} p & \forall\ 0 \leq x \leq \hat{x}, \\ p\Big(\frac{\hat{x}}{x}\Big)^{k(s)} & \forall\ \hat{x} < x, \end{cases} \qquad \bar{q}_{X,s}(u) = \begin{cases} \hat{x}\Big(\frac{p}{u}\Big)^{1/k(s)}, & \text{if } u \in [0, p], \\ 0, & \text{if } u \in (p, 1]. \end{cases}$$

$$\mathbb{E}_s[X] = \frac{p\hat{x}k(s)}{k(s) - 1},\ \ k(s) > 1,\ \forall\ s \geq 0, \qquad \rho_{1,s}(X) = \begin{cases} \frac{\hat{x} r_1 k(s) p^{r_1}}{r_1 k(s) - 1}, & \text{if } k(s) > \frac{1}{r_1}, \\ \infty, & \text{else.} \end{cases}$$

Note that concavity of $s \mapsto k(s)$ implies convexity of $s \mapsto \bar{q}_{X,s}(u)$ for all $u \in (0, 1)$ and that we now assume $k(s) > \frac{1}{r_1}$ for all $s \geq 0$.[44] It follows that $G^\beta(s)$ is given by

$$G^\beta(s) = \frac{\rho_{1,s}(X)}{\mathbb{E}_s[X]} + (\beta_o - \beta)\frac{c(s)}{\mathbb{E}_s[X]} = \frac{r_1 p^{r_1 - 1}(k(s) - 1)}{r_1 k(s) - 1} + (\beta_o - \beta)\frac{\eta s^\gamma (k(s) - 1)}{\hat{x} p k(s)}.$$

Calculating the first two derivatives of the first summand w.r.t. s yields decreasingness and convexity of the ratio, i.e. $\Big(\frac{\rho_{1,s}(X)}{\mathbb{E}_s[X]}\Big)' < 0,\ \ \Big(\frac{\rho_{1,s}(X)}{\mathbb{E}_s[X]}\Big)'' \geq 0$, implying that in this case, Assumption 3 does not hold and indeed the reverse is fulfilled (the risk measure decreases faster than the price as s increases). A sufficient condition for convexity (in s) of the second

[43]The subscripts $_0$ and $_1$ refer again to the insurer and insurance buyer, respectively.

[44]Otherwise, this would simply imply that the insurance buyer would always choose a service level of at least $s > k^{-1}(\frac{1}{r_1})$.

summand of $G^\beta(s)$ is for $c(s)=\eta s^\gamma$ and $k(s)$ to be such that for any $s\geq 0$:

$$k(s)\gamma s^{\gamma-1}k'(s)+k(s)s^\gamma k''(s)-2s^\gamma(k'(s))^2\geq 0. \tag{21}$$

For the remainder of this study we will assume for the service cost $\eta=0.5,\ \gamma=2$ (as in A.5), and for the loss severity $k(s)=\sqrt{s}+z$, where $z>\frac{1}{r_1}$. It is easily checked that Equation (21) then holds, yielding $G^\beta(s)$ as a convex function in s with $\lim\limits_{s\to\infty}G^\beta(s)=\infty$ and by continuity of the derivative decreasing for small $s>0$, as

$$\left(G^\beta(s)\right)'=\underbrace{k'(s)}_{\stackrel{s\to 0}{\longrightarrow}\infty}\Big[\underbrace{\frac{r_1p^{r_1-1}(r_1-1)}{(r_1k(s)-1)^2}}_{\stackrel{s\to 0}{\longrightarrow}\text{const}<0}+\underbrace{\frac{(\beta_o-\beta)\eta s^\gamma}{p\hat{x}k(s)^2}}_{\stackrel{s\to 0}{\longrightarrow}0}\Big]+\underbrace{\frac{(\beta_o-\beta)\eta\gamma}{p\hat{x}}\frac{k(s)-1}{k(s)}s^{\gamma-1}}_{\stackrel{s\to 0}{\longrightarrow}0}\stackrel{s\to 0}{\longrightarrow}-\infty.$$

Therefore, for any β, the convex map $s\mapsto G^\beta(s)$ admits a minimizer s_0 which is an interior point on $(0,\infty)$ characterized by $\left(G^\beta(s)\right)'|_{s=s_0}=0$. The smallest loading $\theta_0(\beta)$ making $G^\beta(s)$ intersect the level $1+\theta$ for any $\theta>\theta_0$ is given by

$$\theta_0(\beta)=G^\beta(s_0)-1,$$

such that it follows

$$\begin{aligned}
&\theta\leq\theta_0(\beta)\implies G^\beta(s)>(1+\theta)\ \forall s\geq 0,\ \mathcal{I}=[0,\infty),\ \mathcal{N}=\emptyset\\
&\theta>\theta_0(\beta)\implies\exists\ 0\leq s_{B1}(\theta,\beta)<s_0<s_{B2}(\theta,\beta)<\infty:\\
&\qquad\qquad\mathcal{I}=\big[0,s_{B1}(\theta,\beta)\big]\cup\big[s_{B2}(\theta,\beta),\infty\big),\ \mathcal{N}=\big(s_{B1}(\theta,\beta),s_{B2}(\theta,\beta)\big),
\end{aligned}$$

where $s_{B1}(\theta,\beta)$ and $s_{B2}(\theta,\beta)$ are defined as

$$\begin{aligned}
s_{B1}(\theta,\beta)&:=\inf\{s\in[0,\infty):G^\beta(s)\leq 1+\theta\},\\
s_{B2}(\theta,\beta)&:=\sup\{s\in[0,\infty):G^\beta(s)\leq 1+\theta\}.
\end{aligned}$$

Note that $s_{B1}(\theta,\beta)$ ($s_{B2}(\theta,\beta)$) is non-increasing (non-decreasing) in θ and β. The economic interpretation is straightforward: As the insurance solution gets more expensive (increase in θ or β), the interval corresponding to the no-insurance solution widens.
For $\theta>\theta_0$, recall that on $\mathcal{N}=\big(s_{B1}(\theta,\beta),s_{B2}(\theta,\beta)\big)$, the insurance buyer minimizes the loss function

$$L_{1,\mathcal{N}}(s)=\rho_{1,s}(X)+\beta_o c(s)=\frac{\hat{x}r_1p^{r_1}k(s)}{r_1k(s)-1}+\beta_o\eta s^\gamma.$$

Thus, the optimality criterion for the global minimizer s_N of $L_{1,\mathcal{N}}(s)$ is given by

$$L'_{1,\mathcal{N}}(s)|_{s=s_N}\stackrel{!}{=}0\iff s_N=\Big[\frac{\hat{x}r_1p^{r_1}k'(s_N)}{\beta_o\eta\gamma(r_1k(s_N)-1)^2}\Big]^{\frac{1}{\gamma-1}},$$

which is an interior point as $\lim\limits_{s\to 0}L'_{1,\mathcal{N}}(s)=-\infty$ and $\lim\limits_{s\to\infty}L_{1,\mathcal{N}}(s)=\infty$.
Analogously, on $\mathcal{I}=[0,s_{B1}(\theta,\beta)]\cup[s_{B2}(\theta,\beta),\infty)$ the insurance buyer minimizes

$$L^{\theta,\beta}_{1,\mathcal{I}}(s)=(1+\theta)\mathbb{E}_s[X]+\beta c(s)=(1+\theta)\frac{\hat{x}pk(s)}{k(s)-1}+\beta\eta s^\gamma.$$

The optimality criterion for the global minimizer $s_I(\theta,\beta)$ is given by

$$[L^{\theta,\beta}_{1,\mathcal{I}}(s)]'|_{s=s_I}\stackrel{!}{=}0\iff s_I(\theta,\beta)=\Big[\frac{(1+\theta)\hat{x}pk'(s_I(\theta,\beta))}{\beta\eta\gamma(k(s_I(\theta,\beta))-1)^2}\Big]^{\frac{1}{\gamma-1}}. \tag{22}$$

Analogously to the self-protection case, one can argue that:

- For any β, at $\theta = 0$ it holds that $\mathcal{I} = [0, \infty)$ and $s_I(\theta, \beta)$ is also the local minimizer on $\mathcal{I}$ and the global minimizer of $L_1(\alpha, s)$ is $(\alpha^*, s^*) = (1, s_I)$.
- Define $\theta_N > 0$ as the smallest $\theta > 0$ such that $s_N \in \mathcal{N}$, i.e. $s_{B1}(\theta, \beta) < s_N$ (if $s_N \leq s_0$) or $s_N < s_{B2}(\theta, \beta)$ (if $s_N > s_0$).
- As both $s_{B2}(\theta, \beta)$ and $s_I(\theta, \beta)$ are non-decr. functions of θ, one has to distinguish the case $s_I \in \mathcal{N}$, i.e. $s_I(\theta, \beta) < s_{B2}(\theta, \beta)$ (in which automatically s_N is the global minimizer and $(\alpha^*, s^*) = (0, s_N)$ the global solution) and the case $s_I \in \mathcal{I}$, i.e. $s_{B2}(\theta, \beta) \leq s_I(\theta, \beta)$, in which one has to compare the function values $L_{1,\mathcal{I}}(s_I)$ and $L_{1,\mathcal{N}}(s_N)$ to determine the global solution.
- As $L_{1,\mathcal{N}}(s_N)$ is independent of θ, while $\theta \mapsto L_{1,\mathcal{I}}(s_I)$ is increasing, one can define the maximum feasible loading as $\theta_R(\beta) := \sup\{\theta \geq 0 : L_{1,\mathcal{I}}(s_I(\theta, \beta)) \leq L_{1,\mathcal{N}}(s_N)\}$, such that for given β for any $\theta > \theta_R(\beta)$ no insurance is preferred, i.e. $(\alpha^*, s^*) = (0, s_N)$.

The insurer's problem is therefore given by

$$\min_{(\theta,\beta)\in[0,\theta_R(\beta)]\times[\underline{\beta},1]} L_0(\theta, \beta) := \frac{\hat{x} r_0 p^{r_0} k(s_I)}{r_0 k(s_I) - 1} - (1+\theta)\frac{\hat{x} p k(s_I)}{k(s_I) - 1} + (1-\beta)\eta s_I^\gamma,$$

where $s_I(\theta, \beta)$ is characterized by Equation (22). In the self-insurance case, Assumption 3 does not hold anymore, and therefore neither do (necessarily) monotonicity of $\beta \mapsto L_0(\theta, \beta)$ (Proposition 1) and monotonicity of $\beta \mapsto L_0(\theta_R(\beta), \beta)$ (Proposition 2). Monotonicity (non-increasingness) of the insurer's loss in θ can be shown for the special case $\beta = 1$ (see [Bensalem et al., 2020]), but not for general $\beta \in [\underline{\beta}, 1]$. Therefore, we resort to numerical optimization (using the `R` package `nloptr`) of the program

$$\min_{\theta,\beta} L_0(\theta, \beta) \;\; s.t. \;\; L_{1,\mathcal{I}}^{\theta,\beta}(s_I(\theta, \beta)) - L_{1,\mathcal{N}}(s_N) \leq 0, \;\; 0 \leq \theta < \infty, \quad \underline{\beta} \leq \beta \leq 1,$$

where the calculations to derive the gradients of the objective (insurer's loss function) and the constraint (insurance buyer's loss function) are given in the following subsection (for $\eta = 0.5$, $\gamma = 2$ as above). The insurance buyer's and insurer's solutions for an exemplary set of parameters are illustrated in Figures 8 and 9, respectively.[45]

- Panel 8a shows the insurance buyer's optimal service demand with and without insurance depending on the loading. As $\theta \mapsto s_I(\theta, \beta)$ is increasing, at a higher loading the insurance buyer has an incentive to purchase more service within insurance (at fixed cost). However, in this case (as remarked in [Bensalem et al., 2020] for the self-insurance case), the jump in s^* at $\theta = \theta_R(0.5)$ is positive (i.e. when switching to the no-insurance solution, more service is demanded), meaning that risk transfer demand and service demand are substitutes (contrary to Corollary 4).
- The left region of Panel 8a corresponds to the case $\theta < \theta_0$ where $\mathcal{I} = [0, \infty)$ and thus $s^* = s_I$. For $\theta \geq \theta_0$, the interval $(s_{B1}(\theta, 0.5), s_{B2}(\theta, 0.5))$ corresponding to the set $\mathcal{N}$ broadens with increasing θ.

[45]The parameters for this example are: for the risk measures $r_1 = 0.5$, $r_0 \in [r_1 + \Delta, 1]$, $\Delta = 10^{-3}$, for the service cost $\eta = 0.5$, $\gamma = 2$, $\beta_o = 1.1$, $\underline{\beta} = 0.05$, for the loss (severity) distribution $\hat{x} = 2$, $p = 0.2$, $k(s) = \sqrt{s} + z$ with $z = \frac{1}{r_1} + 0.1$. For $z \leq \frac{1}{r_1}$, one would need to calculate $\underline{s} := k^{-1}\left(\frac{1}{r_1}\right)$ and restrict the analysis to $s \in (\underline{s}, \infty)$.

- In the right region of Panel 8a, it holds $s_I(\theta, 0.5) \in \mathcal{N}$ implying $s^* = s_N$.
- For values of θ such that $s_I(\theta, 0.5) \in \mathcal{I},\ s_N \in \mathcal{N}$, one compares the insurance buyer's loss (objective function value) for both problems to determine $\theta_R(0.5)$, as illustrated in Panel 8b.
- Panel 9a compares the admissible set $(\theta, \beta) \in [0, \theta_R(\beta)] \times [\underline{\beta}, 1]$ of the insurance buyer (for $r_1 = 0.5$) with the admissible set of the insurer $(\theta, \beta) \in [\theta_{\min}(\beta), \infty) \times [\underline{\beta}, 1]$ (for selected values $r_0 \in (r_1, 1]$), i.e. a mutually acceptable contract exists for β s.t. $\theta_{\min}(\beta) \leq \theta_R(\beta)$.
- Interestingly, $\beta \mapsto \theta_{\min}(\beta)$ is not necessarily monotone decreasing anymore, and in particular if r_0 is close to r_1 (the insurer is almost as risk-averse as the buyer), the case that no acceptable contract can be found does not only occur for small values of β (as observed before), but also for very *high* values of β. As this is perhaps counter-intuitive, it merits an explanation: The more risk-averse the insurer, the more she values risk reduction by the buyer; however, the service amount $s_I(\theta, \beta)$ the buyer is willing to (optimally) purchase to achieve risk reduction with insurance decreases with β, i.e. if service becomes too expensive, the buyer may not be willing to buy as much service as required by a risk-averse insurer. This occurs due to the property of the self-insurance case that for any increase in service, the risk measure decreases faster than the price of insurance and therefore, while buying a unit of service without insurance is relatively more expensive ($\beta_o > 1$), the decrease in risk (which the buyer considers without insurance) may overcompensate this relative to the smaller decrease in price within insurance. This implies that if the risk aversions of buyer and insurer are similar, **a mutually acceptable contract can only be found if the cost of risk reduction service is shared**.
- The bold part of the boundary in Panel 9a marks the set of optimal solutions (θ^*, β^*) obtained by solving (numerically) the Stackelberg game for $r_0 \in [r_1 + \Delta, 1]$. This illustrates that contrary to the self-protection case, $\beta \mapsto L_0(\theta_R(\beta), \beta)$ is not necessarily monotone decreasing anymore.
- The solid line in Panel 9b shows the optimal share of service cost β^* burdened on the buyer depending on the absolute difference $r_0 - r_1$. The more risk-averse the insurer is relative to the buyer, the more she will incentivise risk reduction by *subsidizing* service, i.e. by optimally offering a contract with lower β. If the insurer is much less risk-averse than the buyer, she will not subsidize service any longer ($\beta^* = 1$), as the partial service cost is no longer overcompensated by her subjective gain of insuring a reduced risk.
- The dashed line in Panel 9b shows the optimally attainable value of the insurer's objective function depending on the absolute difference $r_0 - r_1$. Naturally, the insurer's obtainable gain (negative loss) decreases as she becomes more risk-averse.
- While the insurer's loss is again monotone decreasing on $\{(\theta, 1),\ \theta \in [0, \theta_R(1)]\}$ (this was shown for the special Pareto case in [Bensalem et al., 2020]), this is not necessarily the case on $\{(\theta_R(\beta), \beta),\ \beta \in [\underline{\beta}, 1]\}$ anymore. The optimal $\beta^* < 1$ and the corresponding insurer's loss are depicted in Panels 9c and 9d, for the parameter choices $r_0 = 0.6$ and $r_0 = 0.51$ (very close to r_1), respectively. Panel 9d illustrates the phenomenon observed in Panel 9a that the insurer cannot obtain a negative loss for $\beta \to 1$ (as well as for small β).

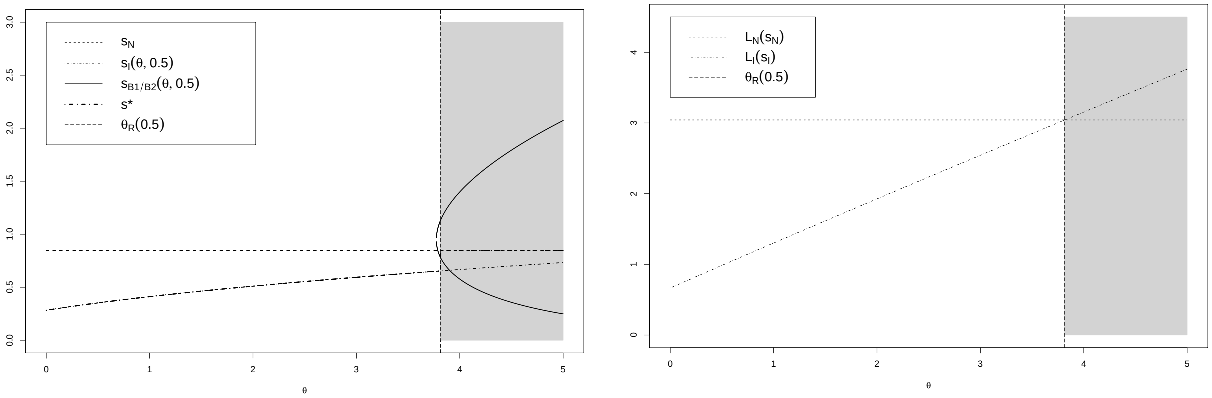

(a) $s_N, s_I(\theta, 0.5)$ and $s_{B1}(\theta, 0.5), s_{B2}(\theta, 0.5)$.

(b) $L_{1,\mathcal{N}}(s_N)$ and $L_{1,\mathcal{I}}^{\theta,0.5}(s_I(\theta, 0.5))$.

Figure 8: The figure illustrates the insurance buyer's solution depending on the loading θ, if the insurance buyer has to bear half of the service cost ($\beta = 0.5$). Gray areas again represent values of θ where no contract is closed.

Derivatives of insurer's loss in the self-insurance case

Recall that the insurer's loss function is given by

$$L_0(\theta, \beta) = \frac{\hat{x} r_0 p^{r_0} k(s_I)}{r_0 k(s_I) - 1} - (1+\theta) \frac{\hat{x} p k(s_I)}{k(s_I) - 1} + (1-\beta) c(s_I).$$

Its partial derivative w.r.t. θ is given by

$$\frac{\partial}{\partial\theta} L_0(\theta, \beta) = \underbrace{-\frac{r_0 p^{r_0} \hat{x} k'(s_I) \frac{\partial}{\partial\theta} s_I(\theta, \beta)}{(r_0 k(s_I) - 1)^2}}_{\leq 0} \underbrace{-p\hat{x} \frac{k(s_I)(k(s_I) - 1) - (1+\theta) k'(s_I) \frac{\partial}{\partial\theta} s_I(\theta, \beta)}{(k(s_I) - 1)^2}}_{<0, \text{ see [Bensalem et al., 2020]}} + \underbrace{(1-\beta) c'(s_I) \frac{\partial}{\partial\theta} s_I(\theta, \beta)}_{\geq 0}, \tag{23}$$

where we use that by assumption $k'(s) \geq 0$, $k''(s) \leq 0$, and to derive $\frac{\partial}{\partial\theta} s_I(\theta, \beta)$, use that s_I is characterized by Equation (22) or equivalently (by rearranging), for the function $F_\beta(\theta, s) := \frac{s\beta(k(s)-1)^2}{p\hat{x}k'(s)} - (1+\theta)$, the tuple $(\theta, s_I(\theta, \beta))$ is a solution to $F_\beta = 0$ for any θ. Then, applying the implicit function theorem (IFT) yields (see [Bensalem et al., 2020])

$$\frac{\partial s_I(\theta, \beta)}{\partial\theta} = -\frac{\frac{\partial F_\beta(\theta, s_I)}{\partial\theta}}{\frac{\partial F_\beta(\theta, s_I)}{\partial s}} = \underbrace{\left(\frac{s_I}{1+\theta}\right)}_{\geq 0} \left(\frac{1}{1 + \underbrace{\frac{2 s_I k'(s_I)}{k(s_I) - 1}}_{>0} \underbrace{- \frac{k''(s_I) s_I}{k'(s_I)}}_{>0}} \right) \geq 0,$$

corroborating that $\theta \mapsto s_I(\theta, \beta)$ is non-decreasing. Equation (23) implies that in the special case $\beta = 1$ (where the third term vanishes), the insurer's loss is decreasing in θ, but this does not necessarily hold for general $\beta \in [\underline{\beta}, 1]$.
Analogously, we derive the partial derivative of the insurer's loss w.r.t. β as

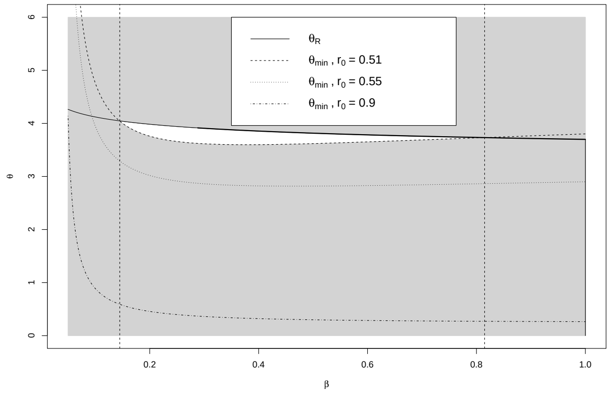

(a) $\theta_R(\beta)$ and $\theta_{min}(\beta)$.

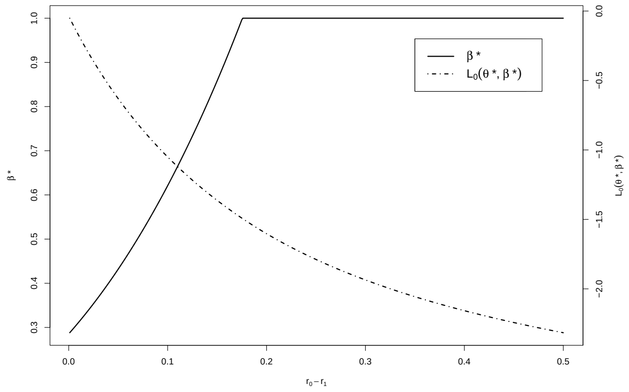

(b) β^* and $L_0(\theta^*, \beta^*)$ as functions of $r_0 - r_1$.

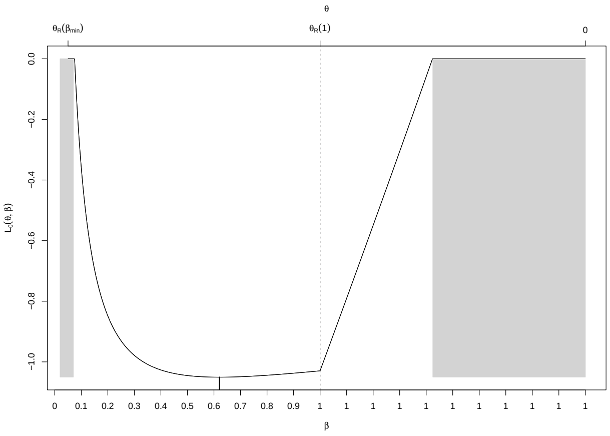

(c) $r_0 = 0.6$.

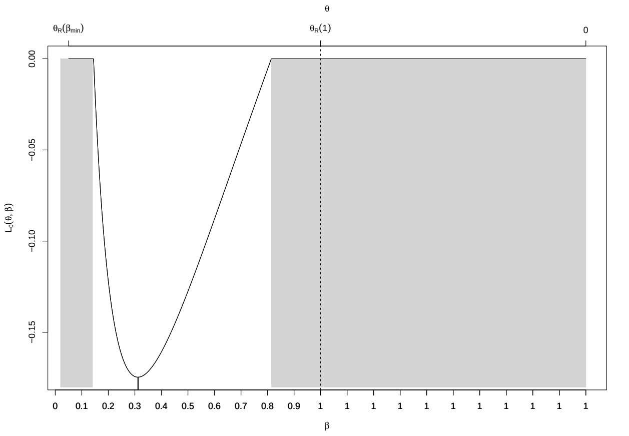

(d) $r_0 = 0.51$.

Figure 9: Illustration of several aspects of the insurer's solution in the self-insurance case. Panels 9c and 9d show the insurer's loss $L_0(\theta_R(\beta), \beta),\ \beta \in [\underline{\beta}, 1]$ and $L_0(\theta, 1),\ \theta \in [0, \underline{\theta} = \theta_R(1)]$.

$$\frac{\partial}{\partial \beta} L_0(\theta, \beta) = \underbrace{-\frac{p^{r_0} r_0 \hat{x} k'(s_I) \frac{\partial}{\partial \beta} s_I(\theta, \beta)}{(r_0 k(s_I) - 1)^2}}_{\geq 0} + \underbrace{\frac{(1+\theta) p \hat{x} k'(s_I) \frac{\partial}{\partial \beta} s_I(\theta, \beta)}{(k(s_I) - 1)^2}}_{\leq 0} + \underbrace{(1-\beta) s_I \frac{\partial}{\partial \beta} s_I(\theta, \beta) - c(s_I)}_{\leq 0},$$

where, analogously to above, consider that s_I is characterized by Equation (22), or equivalently, the tuple $(\beta, s_I(\theta, \beta))$ for any β is a solution to $F_\theta(\beta, s) := \beta - (1+\theta)\frac{p\hat{x}k'(s)}{s(k(s)-1)^2} = 0$. As before, applying the IFT yields

$$\frac{\partial s_I(\theta, \beta)}{\partial \beta} = -\frac{\frac{\partial F_\theta(\beta, s_I)}{\partial \beta}}{\frac{\partial F_\theta(\beta, s_I)}{\partial s}} = \frac{1}{\beta}\left(\underbrace{\frac{k''(s_I)}{k'(s_I)}}_{\leq 0} \underbrace{-\frac{1}{s_I}}_{<0} \underbrace{-\frac{2k'(s_I)}{k(s_I) - 1}}_{\leq 0} \right)^{-1} < 0,$$

corroborating that also in this self-insurance case, $\beta \mapsto s_I(\theta, \beta)$ is non-increasing. Lastly, the gradient of the constraint $L^{\theta,\beta}_{1,\mathcal{I}}(s_I(\theta, \beta)) - L_{1,\mathcal{N}}(s_N) \leq 0$ is given by

$$\frac{\partial L^{\theta,\beta}_{1,\mathcal{I}}(s_I(\theta,\beta))}{\partial\theta} = p\hat{x}\frac{k(s_I)(k(s_I)-1)-(1+\theta)k'(s_I)\frac{\partial}{\partial\theta}s_I(\theta,\beta)}{(k(s_I)-1)^2} + \beta c'(s_I)\frac{\partial}{\partial\theta}s_I(\theta,\beta),$$
$$\frac{\partial L^{\theta,\beta}_{1,\mathcal{I}}(s_I(\theta,\beta))}{\partial\beta} = \frac{-(1+\theta)p\hat{x}k'(s_I)\frac{\partial}{\partial\beta}s_I(\theta,\beta)}{(k(s_I)-1)^2} + \beta c'(s_I)\frac{\partial}{\partial\beta}s_I(\theta,\beta) - c(s_I).$$

A.7 The Insurer's Problem in the Portfolio Case

A.7.1 (Directed) Loss Propagation

With the stated assumptions on $X_i,\ i\in\{1,2\}$, the portfolio loss $X := X_1 + X_2$ and its tail quantile function are given by

$$X = \begin{cases} 0 & \text{w.p. } (1-p_1)(1-p_2), \\ L_1 & \text{w.p. } p_1(1-q)(1-p_2), \\ L_2 & \text{w.p. } (1-p_1)p_2, \\ L_1+L_2 & \text{w.p. } p_1q + p_1(1-q)p_2, \end{cases} \quad \overline{q}_{X,s}(u) = \begin{cases} 0 & u\in(p_1+p_2-p_1p_2,1], \\ L_1 & u\in(p_2+p_1q-p_1p_2q, p_1+p_2-p_1p_2], \\ L_2 & u\in(p_1q+p_1p_2-p_1p_2q, p_2+p_1q-p_1p_2q], \\ L_1+L_2 & u\in[0,p_1q+p_1p_2-p_1p_2q], \end{cases}$$

where the dependence on $s_i,\ i\in\{1,2\}$, is omitted for brevity. The insurer's portfolio risk measure with $\mathbf{s} := (s_1,s_2)$ (for $\psi(u)=u^{r_0},\ r_0\in(0,1)$) is then given by

$$\begin{aligned}\rho_{0,\mathbf{s}}(X) &= L_1[(p_1+p_2-p_1p_2)^{r_0} - (p_2+p_1q-p_1p_2q)^{r_0}] + L_2[(p_2+p_1q-p_1p_2q)^{r_0} \\ &\quad - (p_1q+p_1p_2-p_1p_2q)^{r_0}] + (L_1+L_2)(p_1q+p_1p_2-p_1p_2q)^{r_0} \\ &= L_1[(p_1+p_2-p_1p_2)^{r_0} + (p_1q+p_1p_2-p_1p_2q)^{r_0}] + (L_2-L_1)(p_2+p_1q-p_1p_2q)^{r_0}. \qquad (24)\end{aligned}$$

The general difference between the single-contract and the portfolio case, i.e. that Equation (7) may hold, is illustrated in Figure 3 for an exemplary set of parameters in this case of directed loss propagation.

Calculation of gradients for sequential contract closure (SEQ 21)

Recall the insurer's objective function in Equation (10) for contract 1 is given by

$$L^{\text{seq}}_{0,1}(\theta_1,\beta_1) = \rho_{0,s_{I1}(\theta_1,\beta_1),s_{I2}(\theta_{R,2}(1),1)}(X) - (1+\theta_1)\mathbb{E}_{s_{I1}(\theta_1,\beta_1)}(X_1) + (1-\beta_1)c(s_{I1}(\theta_1,\beta_1)),$$

where the portfolio risk measure is given in (24). Its partial derivatives w.r.t. θ_1 and β_1 are thus given by

$$\begin{aligned}\frac{\partial L^{\text{seq}}_{0,1}(\theta_1,\beta_1)}{\partial\theta_1} &= \Big[L_1\big(r_0(p_1(s_{I1})(1-p_{I2})+p_{I2})^{r_0-1}(1-p_{I2}) + (q+p_{I2}-p_{I2}q)^{r_0}r_0p_1(s_{I1})^{r_0-1}\big) \\ &\quad + (L_2-L_1)r_0\big[p_1(s_{I1})(q-p_{I2}q)+p_{I2}\big]^{r_0-1}(q-p_{I2}q)\Big]p_1'(s_{I1})\frac{\partial s_{I1}}{\partial\theta_1} \\ &\quad - \Big(L_1p_1(s_{I1}) + (1+\theta_1)L_1p_1'(s_{I1})\frac{\partial s_{I1}}{\partial\theta_1}\Big) + (1-\beta_1)c'(s_{I1})\frac{\partial s_{I1}}{\partial\theta_1}, \\ \frac{\partial L^{\text{seq}}_{0,1}(\theta_1,\beta_1)}{\partial\beta_1} &= \Big[L_1\big(r_0(p_1(s_{I1})(1-p_{I2})+p_{I2})^{r_0-1}(1-p_{I2}) + (q+p_{I2}-p_{I2}q)^{r_0}r_0p_1(s_{I1})^{r_0-1}\big) \\ &\quad + (L_2-L_1)r_0\big[p_1(s_{I1})(q-p_{I2}q)+p_{I2}\big]^{r_0-1}(q-p_{I2}q)\Big]p_1'(s_{I1})\frac{\partial s_{I1}}{\partial\beta_1} \\ &\quad - (1+\theta_1)L_1p_1'(s_{I1})\frac{\partial s_{I1}}{\partial\beta_1} - c(s_{I1}) + (1-\beta_1)c'(s_{I1})\frac{\partial s_{I1}}{\partial\beta_1},\end{aligned}$$

where the dependence $s_{I1}(\theta_1,\beta_1)$ is omitted for brevity and $p_{I2} := p_2(s_{I2})$. Analogously to above, to derive $\frac{\partial s_{I1}}{\partial \theta_1}$ and $\frac{\partial s_{I1}}{\partial \beta_1}$, use that s_{I1} is characterized by

$$\big[L^{\theta_1,\beta_1}_{\mathcal{I},1}(s_1)\big]'|_{s_1=s_{I1}} \overset{!}{=} 0 \iff s_{I1}(\theta_1,\beta_1) = \Big[-\frac{(1+\theta_1)L_1}{\beta_1\eta\gamma}p_1'(s_{I1})\Big]^{\frac{1}{\gamma-1}} \overset{\eta=0.5,\gamma=2}{=} -\frac{(1+\theta_1)L_1 p'(s_{I1})}{\beta_1}. \quad (25)$$

Rearranging (25) yields that (θ_1, s_{I1}) is a solution to $F_{\beta_1}(\theta_1,s) := \frac{s\beta_1}{L_1 p_1'(s)} + (1+\theta_1) = 0$ for any θ_1 and likewise (β_1, s_{I1}) is a solution to $F_{\theta_1}(\beta_1,s) := \beta_1 + (1+\theta_1)L_1\frac{p_1'(s)}{s} = 0$ for any β_1. Applying IFT then yields

$$\frac{\partial s_{I1}(\theta_1,\beta_1)}{\partial\theta_1} = -\frac{\frac{\partial F_{\beta_1}(\theta_1,s)}{\partial\theta_1}|_{s=s_{I1}}}{\frac{\partial F_{\beta_1}(\theta_1,s)}{\partial s}|_{s=s_{I1}}} = \frac{-L_1 p_1'(s_{I1})}{\beta_1\Big(1-\frac{s_{I1}p_1''(s_{I1})}{p_1'(s_{I1})}\Big)} > 0,$$
$$\frac{\partial s_{I1}(\theta_1,\beta_1)}{\partial\beta_1} = -\frac{\frac{\partial F_{\theta_1}(\beta_1,s)}{\partial\beta_1}|_{s=s_{I1}}}{\frac{\partial F_{\theta_1}(\beta_1,s)}{\partial s}|_{s=s_{I1}}} = -\frac{s_{I1}}{\Big(p_1''(s_{I1})-\frac{p_1'(s_{I1})}{s_{I1}}\Big)L_1(1+\theta_1)} < 0.$$

The partial derivatives of the constraint $L^{\theta_1,\beta_1}_{\mathcal{I},1}(s_{I1}(\theta_1,\beta_1)) - L_{\mathcal{N},1}(s_{N1}) \le 0$ are

$$\frac{\partial}{\partial\theta_1}L^{\theta_1,\beta_1}_{\mathcal{I},1}(s_{I1}(\theta_1,\beta_1)) = L_1p_1(s_{I1}) + (1+\theta_1)L_1p_1'(s_{I1})\frac{\partial s_{I1}}{\partial\theta_1} + \beta_1 c'(s_{I1})\frac{\partial s_{I1}}{\partial\theta_1},$$
$$\frac{\partial}{\partial\beta_1}L^{\theta_1,\beta_1}_{\mathcal{I},1}(s_{I1}(\theta_1,\beta_1)) = (1+\theta_1)L_1p_1'(s_{I1})\frac{\partial s_{I1}}{\partial\beta_1} + c(s_{I1}) + \beta_1 c'(s_{I1})\frac{\partial s_{I1}}{\partial\beta_1}.$$

Calculation of gradients for sequential contract closure (SEQ 12)
If the contracts are closed in reverse sequential order, the insurer's objective function for contract 2 is

$$L^{\text{seq}}_{0,2}(\theta_2,\beta_2) = \rho_{0,s_{I1}(\theta_{R,1}(1),1),s_{I2}(\theta_2,\beta_2)}(X) - (1+\theta_2)\mathbb{E}_{s_{I2}(\theta_2,\beta_2)}(X_2) + (1-\beta_2)c(s_{I2}(\theta_2,\beta_2)),$$

yielding its partial derivatives w.r.t. θ_2 and β_2 as

$$\begin{aligned}\frac{\partial L^{\text{seq}}_{0,2}(\theta_2,\beta_2)}{\partial\theta_2} =& \Big[L_1\big(r_0(p_{I1}+(1-p_{I1})p_2(s_{I2}))^{r_0-1}(1-p_{I1}) + r_0(p_{I1}q+p_{I1}(1-q)p_2(s_{I2}))^{r_0-1}p_{I1}(1-q)\big)\\ &+ (L_2-L_1)r_0\big[p_{I1}q+(1-qp_{I1})p_2(s_{I2})\big]^{r_0-1}(1-qp_{I1})\Big]p_2'(s_{I2})\frac{\partial s_{I2}}{\partial\theta_2}\\ &-\Big(L_2(p_{I1}q+(1-p_{I1}q)p_2(s_{I2})) + (1+\theta_2)L_2(1-p_{I1}q)p_2'(s_{I2})\frac{\partial s_{I2}}{\partial\theta_2}\Big) + (1-\beta_2)c'(s_{I2})\frac{\partial s_{I2}}{\partial\theta_2},\\ \frac{\partial L^{\text{seq}}_{0,2}(\theta_2,\beta_2)}{\partial\beta_2} =& \Big[L_1\big(r_0(p_{I1}+(1-p_{I1})p_2(s_{I2})))^{r_0-1}(1-p_{I1}) + r_0(p_{I1}q+p_{I1}(1-q)p_2(s_{I2}))^{r_0-1}p_{I1}(1-q)\\ &+ (L_2-L_1)r_0\big[p_{I1}q+(1-qp_{I1})p_2(s_{I2})\big]^{r_0-1}(1-qp_{I1})\Big]p_2'(s_{I2})\frac{\partial s_{I2}}{\partial\beta_2}\\ &-(1+\theta_2)L_2(1-p_{I1}q)p_2'(s_{I2})\frac{\partial s_{I2}}{\partial\beta_2} - c(s_{I2}) + (1-\beta_2)c'(s_{I2})\frac{\partial s_{I2}}{\partial\beta_2},\end{aligned}$$

where the dependence $s_{I2}(\theta_2,\beta_2)$ is omitted, again $p_{I1} := p_1(s_{I1})$, and the partial derivatives $\frac{\partial s_{I2}}{\partial\theta_2}$ and $\frac{\partial s_{I2}}{\partial\beta_2}$ are derived analogously to above as

$$\frac{\partial s_{I2}(\theta_2,\beta_2)}{\partial\theta_2} = \frac{-L_2(1-p_1q)p_2'(s_{I2})}{\beta_2\Big(1-\frac{s_{I2}p_2''(s_{I2})}{p_2'(s_{I2})}\Big)} > 0,$$
$$\frac{\partial s_{I2}(\theta_2,\beta_2)}{\partial\beta_2} = -\frac{s_{I2}}{\Big(p_2''(s_{I2})-\frac{p_2'(s_{I2})}{s_{I2}}\Big)L_2(1+\theta_2)(1-p_1q)} < 0.$$

The partial derivatives of the constraint $L_{\mathcal{I},2}^{\theta_2,\beta_2}(s_{I2}(\theta_2,\beta_2)) - L_{\mathcal{N},2}(s_{N2}) \le 0$ are

$$\frac{\partial}{\partial\theta_2}L_{\mathcal{I},2}^{\theta_2,\beta_2}(s_{I2}(\theta_2,\beta_2)) = L_2(p_{I1}q+p_2(s_{I2})(1-p_1q)) + (1+\theta_2)L_2(1-p_1q)p_2'(s_{I2})\frac{\partial s_{I2}}{\partial\theta_2} + \beta_2c'(s_{I2})\frac{\partial s_{I2}}{\partial\theta_2},$$
$$\frac{\partial}{\partial\beta_2}L_{\mathcal{I},2}^{\theta_2,\beta_2}(s_{I2}(\theta_2,\beta_2)) = (1+\theta_2)L_2(1-p_1q)p_2'(s_{I2})\frac{\partial s_{I2}}{\partial\beta_2} + c(s_{I2}) + \beta_2c'(s_{I2})\frac{\partial s_{I2}}{\partial\beta_2}.$$

Calculation of gradients for simultaneous contract closure (SIM)
Recall that if the contracts are closed simultaneously, the insurer solves the four-dimensional problem stated in Equation (11), minimizing the loss function

$$\begin{aligned}
L_0^{\text{sim}}(\theta_1,\beta_1,\theta_2,\beta_2) &= \rho_{0,s_{I1}(\theta_1,\beta_1),s_{I2}(\theta_1,\beta_1,\theta_2,\beta_2)}(X)\\
&\quad-(1+\theta_1)\mathbb{E}_{s_{I1}(\theta_1,\beta_1)}[X_1]-(1+\theta_2)\mathbb{E}_{s_{I2}(\theta_1,\beta_1,\theta_2,\beta_2)}[X_2]\\
&\quad+(1-\beta_1)c(s_{I1}(\theta_1,\beta_1))+(1-\beta_2)c(s_{I2}(\theta_1,\beta_1,\theta_2,\beta_2))\\
&=: f_1(\theta_1,\beta_1,\theta_2,\beta_2)-f_2(\theta_1,\beta_1,\theta_2,\beta_2)-f_3(\theta_1,\beta_1,\theta_2,\beta_2)\\
&\quad+f_4(\theta_1,\beta_1,\theta_2,\beta_2)+f_5(\theta_1,\beta_1,\theta_2,\beta_2),
\end{aligned}$$

on the admissible set $\mathcal{A} := [0,\theta_{R,1}(\beta_1)]\times[\underline{\beta},1]\times[0,\theta_{R,2}(\beta_2)]\times[\underline{\beta},1]$. Note that

- Due to the directed nature of loss propagation, s_{I1} does not depend on θ_2,β_2,s_{I2}, implying $\frac{\partial f_2}{\partial\theta_2}=\frac{\partial f_2}{\partial\beta_2}=\frac{\partial f_4}{\partial\theta_2}=\frac{\partial f_4}{\partial\beta_2}=0$.
- The derivatives of the portfolio risk measure and the price of insurance and service cost for firm 2, i.e. $\frac{\partial f_1}{\partial\theta_2}$, $\frac{\partial f_1}{\partial\beta_2}$, $\frac{\partial f_3}{\partial\theta_2}$, $\frac{\partial f_3}{\partial\beta_2}$, $\frac{\partial f_5}{\partial\theta_2}$, $\frac{\partial f_5}{\partial\beta_2}$, are as in (SEQ 12).
- Analogously, the partial derivatives of the price of insurance and service cost for firm 1, i.e. $\frac{\partial f_2}{\partial\theta_1}$, $\frac{\partial f_2}{\partial\beta_1}$, $\frac{\partial f_4}{\partial\theta_1}$, $\frac{\partial f_4}{\partial\beta_1}$, are as in (SEQ 21).

The remaining derivatives w.r.t. θ_1 are given by:

$$\begin{aligned}
\frac{\partial f_1(\theta_1,\beta_1,\theta_2,\beta_2)}{\partial\theta_1} &= L_1\Big[r_0(p_{I1}+p_{I2}-p_{I1}p_{I2})^{r_0-1}\big[p_1'(s_{I1})\frac{\partial s_{I1}}{\partial\theta_1}(1-p_{I2})+p_2'(s_{I2})\frac{\partial s_{I2}}{\partial\theta_1}(1-p_{I1})\big]\\
&\quad+r_0(p_{I1}q+p_{I1}p_{I2}-p_{I1}p_{I2}q)^{r_0-1}\big[(p_{I2}+q-p_{I2}q)p_1'(s_{I1})\frac{\partial s_{I1}}{\partial\theta_1}+p_{I1}(1-q)p_2'(s_{I2})\frac{\partial s_{I2}}{\partial\theta_1}\big]\Big]\\
&\quad+(L_2-L_1)r_0(p_{I1}q-p_{I1}p_{I2}q+p_{I2})^{r_0-1}\Big[q(1-p_2)p_1'(s_{I1})\frac{\partial s_{I1}}{\partial\theta_1}+(1-p_{I1}q)p_2'(s_{I2})\frac{\partial s_{I2}}{\partial\theta_1}\Big],\\
\frac{\partial f_3(\theta_1,\beta_1,\theta_2,\beta_2)}{\partial\theta_1} &= (1+\theta_2)L_2\Big((1-p_{I2})qp_1'(s_{I1})\frac{\partial s_{I1}}{\partial\theta_1}+(1-p_{I1}q)p_2'(s_{I2})\frac{\partial s_{I2}}{\partial\theta_1}\Big),\\
\frac{\partial f_5(\theta_1,\beta_1,\theta_2,\beta_2)}{\partial\theta_1} &= (1-\beta_2)c'(s_{I2})\frac{\partial s_{I2}}{\partial\theta_1}.
\end{aligned}$$

To derive $\frac{\partial s_{I2}}{\partial \theta_1}$, recall that s_{I2} is characterized by $\frac{(1+\theta_2)L_2(1-qp_{I1})p_2'(s_{I2})}{\beta_2} + s_{I2} = 0$, thus for $F_{\theta_2,\beta_2}(s_1, s_2) := \frac{(1+\theta_2)L_2(1-qp_1(s_1))p_2'(s_2)}{\beta_2} + s_2$, it follows as above

$$\frac{\partial s_{I2}}{\partial s_{I1}} = -\frac{\frac{\partial F_{\theta_2,\beta_2}(s_1,s_2)}{\partial s_1}}{\frac{\partial F_{\theta_2,\beta_2}(s_1,s_2)}{\partial s_2}} = \frac{(1+\theta_2)L_2 p_2'(s_{I2})qp_1'(s_{I1})}{(1+\theta_2)L_2(1-qp_{I1})p_2''(s_{I2}) + \beta_2}$$

and by the chain rule $\frac{\partial s_{I2}}{\partial \theta_1} = \frac{\partial s_{I2}}{\partial s_{I1}} \cdot \frac{\partial s_{I1}}{\partial \theta_1}$ where $\frac{\partial s_{I1}}{\partial \theta_1}$ has been calculated in (SEQ 21) above. The partial derivatives w.r.t. β_1 are derived analogously as

$$\begin{aligned}
\frac{\partial f_1(\theta_1,\beta_1,\theta_2,\beta_2)}{\partial \beta_1} &= L_1\Big[r_0(p_{I1}+p_{I2}-p_{I1}p_{I2})^{r_0-1}\big[p_1'(s_{I1})\frac{\partial s_{I1}}{\partial \beta_1}(1-p_{I2}) + p_2'(s_{I2})\frac{\partial s_{I2}}{\partial \beta_1}(1-p_{I1})\big] \\
&\quad + r_0(p_{I1}q + p_{I1}p_{I2} - p_{I1}p_{I2}q)^{r_0-1}\big[(p_{I2}+q-p_{I2}q)p_1'(s_{I1})\frac{\partial s_{I1}}{\partial \beta_1} + p_{I1}(1-q)p_2'(s_{I2})\frac{\partial s_{I2}}{\partial \beta_1}\big]\Big] \\
&\quad + (L_2-L_1)r_0(p_{I1}q - p_{I1}p_{I2}q + p_{I2})^{r_0-1}\Big[q(1-p_2)p_1'(s_{I1})\frac{\partial s_{I1}}{\partial \beta_1} + (1-p_{I1}q)p_2'(s_{I2})\frac{\partial s_{I2}}{\partial \beta_1}\Big], \\
\frac{\partial f_3(\theta_1,\beta_1,\theta_2,\beta_2)}{\partial \beta_1} &= (1+\theta_2)L_2\Big((1-p_{I2})qp_1'(s_{I1})\frac{\partial s_{I1}}{\partial \beta_1} + (1-p_{I1}q)p_2'(s_{I2})\frac{\partial s_{I2}}{\partial \beta_1}\Big), \\
\frac{\partial f_5(\theta_1,\beta_1,\theta_2,\beta_2)}{\partial \beta_1} &= (1-\beta_2)c'(s_{I2})\frac{\partial s_{I2}}{\partial \beta_1},
\end{aligned}$$

where $\frac{\partial s_{I2}}{\partial \beta_1} = \frac{\partial s_{I2}}{\partial s_{I1}} \cdot \frac{\partial s_{I1}}{\partial \beta_1}$ and $\frac{\partial s_{I1}}{\partial \beta_1}$ has been derived above.
In this case, both constraints

$$\begin{aligned}
&L_{\mathcal{I},1}^{\theta_1,\beta_1}(s_{I1}(\theta_1,\beta_1)) - L_{\mathcal{N},1}(s_{N1}) \leq 0, \\
&L_{\mathcal{I},2}^{\theta_1,\beta_1,\theta_2,\beta_2}(s_{I2}(\theta_1,\beta_1,\theta_2,\beta_2)) - L_{\mathcal{N},2}(s_{N2}) \leq 0,
\end{aligned}$$

have to be fulfilled simultaneously. Again $\frac{\partial L_{\mathcal{I},1}^{\theta_1,\beta_1}(s_{I1}(\theta_1,\beta_1))}{\partial \theta_2} = \frac{\partial L_{\mathcal{I},1}^{\theta_1,\beta_1}(s_{I1}(\theta_1,\beta_1))}{\partial \beta_2} = 0$, and $\frac{\partial L_{\mathcal{I},1}^{\theta_1,\beta_1}(s_{I1}(\theta_1,\beta_1))}{\partial \theta_1}$, $\frac{\partial L_{\mathcal{I},1}^{\theta_1,\beta_1}(s_{I1}(\theta_1,\beta_1))}{\partial \beta_1}$ and $\frac{\partial L_{\mathcal{I},2}^{\theta_1,\beta_1,\theta_2,\beta_2}(s_{I2}(\theta_1,\beta_1,\theta_2,\beta_2))}{\partial \theta_2}$, $\frac{\partial L_{\mathcal{I},2}^{\theta_1,\beta_1,\theta_2,\beta_2}(s_{I2}(\theta_1,\beta_1,\theta_2,\beta_2))}{\partial \beta_2}$ have been calculated above for sequential contract closure. To implement the numerical optimization routine, it remains to compute

$$\begin{aligned}
\frac{\partial L_{\mathcal{I},2}^{\theta_1,\beta_1,\theta_2,\beta_2}(s_{I2})}{\partial \theta_1} &= (1+\theta_2)L_2\Big((1-p_{I2})qp_1'(s_{I1})\frac{\partial s_{I1}}{\partial \theta_1} + (1-p_{I1}q)p_2'(s_{I2})\frac{\partial s_{I2}}{\partial \theta_1}\Big) + \beta_2 c'(s_{I2})\frac{\partial s_{I2}}{\partial \theta_1}, \\
\frac{\partial L_{\mathcal{I},2}^{\theta_1,\beta_1,\theta_2,\beta_2}(s_{I2})}{\partial \beta_1} &= (1+\theta_2)L_2\Big((1-p_{I2})qp_1'(s_{I1})\frac{\partial s_{I1}}{\partial \beta_1} + (1-p_{I1}q)p_2'(s_{I2})\frac{\partial s_{I2}}{\partial \beta_1}\Big) + \beta_2 c'(s_{I2})\frac{\partial s_{I2}}{\partial \beta_1}.
\end{aligned}$$

A.7.2 Cyber Events at Multiple Targets

For X_1, X_2 as above, let $Z_1 := \min\{E_1, E_{12}\}$ and $Z_2 := \min\{E_2, E_{12}\}$. Then, the portfolio loss $X := X_1 + X_2$ is described by

$$X = \begin{cases} 0 & \text{if } Z_1 > T,\ Z_2 > T \implies \mathbb{P}(X=0) = e^{-(\lambda_1+\lambda_2+\lambda_{12})} =: y_{00}, \\ L_1 & \text{if } Z_1 \leq T,\ Z_2 > T \implies \mathbb{P}(X=L_1) = (1-e^{-\lambda_1})e^{-(\lambda_2+\lambda_{12})} =: y_{10}, \\ L_2 & \text{if } Z_2 \leq T,\ Z_1 > T \implies \mathbb{P}(X=L_2) = (1-e^{-\lambda_2})e^{-(\lambda_1+\lambda_{12})} =: y_{01}, \\ L_1+L_2 & \text{if } Z_1 \leq T,\ Z_2 \leq T \implies \mathbb{P}(X=L_1+L_2) = y_{11} := 1-(y_{00}+y_{10}+y_{01}), \end{cases}$$

$$\overline{F}_X(x) = \begin{cases} 1 & x < 0, \\ 1 - y_{00} & 0 \leq x < L_1, \\ 1 - (y_{00} + y_{10}) & L_1 \leq x < L_2, \\ 1 - (y_{00} + y_{10} + y_{01}) & L_2 \leq x < L_1 + L_2, \\ 0 & L_1 + L_2 \leq x. \end{cases}$$

The risk measure of the portfolio loss is thus

$$\begin{aligned} \rho(X) = L_1[(1 - y_{00})^r - (1 - (y_{00} + y_{10}))^r] + L_2[(1 - (y_{00} + y_{10}))^r - (1 - (y_{00} + y_{10} + y_{01}))^r] \\ + (L_1 + L_2)(1 - (y_{00} + y_{10} + y_{01}))^r \\ = L_1[(1 - y_{00})^r + (1 - (y_{00} + y_{10} + y_{01}))^r] + (L_2 - L_1)(1 - (y_{00} + y_{10}))^r. \end{aligned}$$

Prevention of systemic events: Extended calculations

By the assumptions above, the expectation and insurer's risk measure for the contract of firm 1 are given by

$$\mathbb{E}_{s_1}[X_1] = L_1(1 - e^{-(\lambda_1 + \lambda_{12}(s_1))}), \quad \rho_{0,s_1}(X_1) = L_1(1 - e^{-(\lambda_1 + \lambda_{12}(s_1))})^{r_0},$$

while the insurer's portfolio risk measure is given by

$$\rho_{0,\mathbf{s}}(X) = L_1((1 - y_{00}(\mathbf{s}))^{r_0} + (1 - y_{00}(\mathbf{s}) - y_{10}(\mathbf{s}) - y_{01}(\mathbf{s}))^{r_0}) + (L_2 - L_1)(1 - y_{00}(\mathbf{s}) - y_{10}(\mathbf{s}))^{r_0},$$

where

$$\begin{aligned} y_{00}(\mathbf{s}) &:= e^{-(\lambda_1 + \lambda_2(s_2) + \lambda_{12}(s_1))} & &\implies \frac{\partial y_{00}(\mathbf{s})}{\partial s_1} = -y_{00}(\mathbf{s})\lambda'_{12}(s_1), \\ y_{10}(\mathbf{s}) &:= (1 - e^{-\lambda_1})e^{-(\lambda_2(s_2) + \lambda_{12}(s_1))} & &\implies \frac{\partial y_{10}(\mathbf{s})}{\partial s_1} = -y_{10}(\mathbf{s})\lambda'_{12}(s_1), \\ y_{01}(\mathbf{s}) &:= (1 - e^{-\lambda_2(s_2)})e^{-(\lambda_1 + \lambda_{12}(s_1))} & &\implies \frac{\partial y_{01}(\mathbf{s})}{\partial s_1} = -y_{01}(\mathbf{s})\lambda'_{12}(s_1). \end{aligned}$$

The derivative of the insurer's portfolio risk measure w.r.t. s_1 is given by

$$\begin{aligned} \frac{\partial \rho_{0,\mathbf{s}}(X)}{\partial s_1} &= L_1\Big[r_0(1 - y_{00}(\mathbf{s}))^{r_0 - 1}\Big(-\frac{\partial y_{00}(\mathbf{s})}{\partial s_1}\Big) + r_0(1 - y_{00}(\mathbf{s}) - y_{10}(\mathbf{s}) - y_{01}(\mathbf{s}))^{r_0 - 1} \\ &\Big(-\frac{\partial y_{00}(\mathbf{s}) + y_{10}(\mathbf{s}) + y_{01}(\mathbf{s})}{\partial s_1}\Big)\Big] + (L_2 - L_1)r_0(1 - y_{00}(\mathbf{s}) - y_{10}(\mathbf{s}))^{r_0 - 1}\Big(-\frac{\partial y_{00}(\mathbf{s}) + y_{10}(\mathbf{s})}{\partial s_1}\Big) \\ &= L_1\Big[r_0(1 - y_{00}(\mathbf{s}))^{r_0 - 1}y_{00}(\mathbf{s})\lambda'_{12}(s_1) + r_0\big(1 - y_{00}(\mathbf{s}) - y_{10}(\mathbf{s}) - y_{01}(\mathbf{s})\big)^{r_0 - 1} \\ &(y_{00}(\mathbf{s}) + y_{10}(\mathbf{s}) + y_{01}(\mathbf{s}))\lambda'_{12}(s_1)\Big] + (L_2 - L_1)r_0(1 - y_{00}(\mathbf{s}) - y_{10}(\mathbf{s}))^{r_0 - 1}(y_{00}(\mathbf{s}) + y_{10}(\mathbf{s}))\lambda'_{12}(s_1). \end{aligned} \tag{26}$$

Using that s_{I1} is characterized by

$$[L_{\mathcal{I},1}^{\theta_1,\beta_1}(s_1)]'|_{s_1 = s_{I1}} = (1 + \theta_1)L_1 e^{-(\lambda_1 + \lambda_{12}(s_{I1}))}\lambda'_{12}(s_{I1}) + \beta_1 c'(s_{I1}) = 0,$$

similar calculations to Subsection A.7.1 yield the derivatives of s_{I1} w.r.t. θ_1 and β_1 as

$$\frac{\partial s_{I1}}{\partial \theta_1} = -\frac{L_1 e^{-(\lambda_1 + \lambda_{12}(s_{I1}))}\lambda'_{12}(s_{I1})}{\beta_1\Big(1 + s_{I1}\lambda'_{12}(s_{I1}) - s_{I1}\frac{\lambda''_{12}(s_{I1})}{\lambda'_{12}(s_{I1})}\Big)}, \tag{27}$$

$$\frac{\partial s_{I1}}{\partial \beta_1} = \frac{s_{I1}}{(1 + \theta_1)L_1 e^{-(\lambda_1 + \lambda_{12}(s_{I1}))}\Big(\lambda'_{12}(s_{I1})^2 - \lambda''_{12}(s_{I1}) + \frac{\lambda'_{12}(s_{I1})}{s_{I1}}\Big)}, \tag{28}$$

such that the gradient of the constraint $L_{\mathcal{I},1}(s_{I1}(\theta_1,\beta_1)) - L_{\mathcal{N},1}(s_{N1}) \leq 0$ w.r.t. (θ_1,β_1) is given by

$$\frac{\partial L^{\theta_1,\beta_1}_{\mathcal{I},1}(s_{I1})}{\partial\theta_1} = L_1(1-e^{-(\lambda_1+\lambda_{12}(s_{I1}))}) + (1+\theta_1)L_1 e^{-(\lambda_1+\lambda_{12}(s_{I1}))}\lambda'_{12}(s_{I1})\frac{\partial s_{I1}}{\partial\theta_1} + \beta_1 c'(s_{I1})\frac{\partial s_{I1}}{\partial\theta_1},$$
$$\frac{\partial L^{\theta_1,\beta_1}_{\mathcal{I},1}(s_{I1})}{\partial\beta_1} = (1+\theta_1)L_1 e^{-(\lambda_1+\lambda_{12}(s_{I1}))}\lambda'_{12}(s_{I1})\frac{\partial s_{I1}}{\partial\beta_1} + c(s_{I1}) + \beta_1 c'(s_{I1})\frac{\partial s_{I1}}{\partial\beta_1}.$$

In the case of sequential contract closure, the insurer's objective function is

$$L^{\text{seq}}_{0,1}(\theta_1,\beta_1) = \rho_{0,s_{I1}(\theta_1,\beta_1),s_{I2}(\theta_{R,2}(1),1)} - (1+\theta_1)\mathbb{E}_{s_{I1}(\theta_1,\beta_1)}(X_1) + (1-\beta_1)c(s_{I1(\theta_1,\beta_1)}),$$

where the derivatives of the portfolio risk measure w.r.t. θ_1 and β_1 are

$$\frac{\partial\rho_{0,s_{I1}(\theta_1,\beta_1),s_{I2}(\theta_{R,2}(1),1)}}{\partial\theta_1} = \underbrace{\frac{\partial\rho_{0,\mathbf{s}}(X)}{\partial s_1}}_{(26)}|_{s_1=s_{I1}}\underbrace{\frac{\partial s_{I1}(\theta_1,\beta_1)}{\partial\theta_1}}_{(27)},$$
$$\frac{\partial\rho_{0,s_{I1}(\theta_1,\beta_1),s_{I2}(\theta_{R,2}(1),1)}}{\partial\beta_1} = \underbrace{\frac{\partial\rho_{0,\mathbf{s}}(X)}{\partial s_1}}_{(26)}|_{s_1=s_{I1}}\underbrace{\frac{\partial s_{I1}(\theta_1,\beta_1)}{\partial\beta_1}}_{(28)}.$$

A.7.3 Cyber Events at Multiple Targets: A Multivariate Example

We now generalize the idea behind the bivariate example in Section 5.2 to a larger portfolio. While the qualitative differences to the univariate case regarding cost-sharing can already be observed in the bivariate case, this portfolio treatment gives an indication of how to generalise the underlying idea for a specific assumption of dependence through common cyber events. We use an adapted version of the setting in [Zeller and Scherer, 2021] as follows:

- **Existing portfolio:** Assume the existing portfolio consists of $(N-1) \geq 1$ homogeneous companies indexed $j \in \{1,\ldots,N-1\}$, whose contracts were priced on an individual basis previously with some risk loading $\theta_{fix} > 0$ (and implicitly $\beta = 1$). One important characteristic of each company is its IT security level, denoted here by $\ell_j \in [0,1]$, which encodes the company's ability to withstand systemic attacks (see below). We assume a homogeneous level of $\ell_j = \ell_{fix} \in [0,1)$, $j \in \{1,\ldots,N-1\}$, for the existing portfolio.

- **Arrival of cyber incidents and events:** Cyber incidents at each company stem from two independent Poisson arrival processes, namely from idiosyncratic incidents (independently from other companies) and systemic events (where multiple companies are affected jointly). We assume that an arrival from a systemic event can affect each company in the portfolio with equal probability $p_{syst} \in [0,1]$. Denote the random subset of the portfolio affected by an event as $\mathcal{S} \subseteq \{1,\ldots,N-1\} \cup \{N\}$. Each event arrival is furthermore equipped with a mark $m \sim Unif([0,1])$ encoding the strength of the attack, where a company which is affected by an event suffers a loss iff the strength of the attack exceeds the company's security level, i.e. $\ell_j < m$. In summary, for each company one can write

the number of cyber losses in the period $[0, T]$ (in the following w.l.o.g. $T = 1$), denoted $N_j(T)$, as the sum of two independent Poisson r.v.s:

$$\begin{aligned}
&N_j(T) = N_j^{idio}(T) + N_j^{syst}(T),\\
&N_j^{idio}(T) \sim Poi(\lambda_j^{idio}\, T),\\
&N_j^{syst}(T) \sim Poi(\lambda^g\, T\, p_{syst}\, (1 - \ell_j)),
\end{aligned}$$

where λ_j^{idio} is the arrival rate of idiosyncratic cyber losses and λ^g is the fixed rate of the *ground process* of systemic cyber events.

As an additional company, indexed N, is to be added to the portfolio, the insurer seeks to price the new contract by choosing (θ_N, β_N). The insurer's choice will again induce a choice of service level within insurance, denoted $s_I(\theta_N, \beta_N)$, by the buyer, which will in turn affect his security level, denoted $\ell(s_I)$. Analogously to the bivariate example, we assume the following:

- **Effect of service on IT security level:** Initially, i.e. when approaching the insurer, buyer N has IT security level $\ell_0 \in [0, 1)$. By purchasing service at level $s \in [0, \infty)$, he can improve his security level according to

$$\ell(s) = 1 - \frac{1}{\frac{1}{1-\ell_0} + s},$$

  such that $s \mapsto \ell(s)$ is increasing and concave with $\ell(0) = \ell_0$ and $\lim_{s\to\infty} \ell(s) = 1$.

- **Loss probability for individual company:** By combining the above assumptions, one can use the properties of the Poisson distribution to express the loss probability for an individual company $j \in \{1, \dots, N\}$ as

$$p(s) = \mathbb{P}(N_j(T) \geq 1) = 1 - \mathbb{P}(N_j(T) = 0) = 1 - \exp\Big(- T\big(\lambda_j^{idio} + \lambda^g\, p_{syst}\, (1 - \ell_j(s))\big)\Big), \tag{29}$$

  such that again $s \mapsto p(s)$ is decreasing.[46]

- **Mitigation of systemic events through warning mechanism:** In order to replicate the mechanism of prevention of systemic events by self-protection described in Remark 20, we assume the following: the companies affected by each common cyber event are targeted in a random order over time, represented by a random permutation of $\mathcal{S}$. The first company which is well-enough protected to withstand the attack furthermore enables the insurer to trigger a warning mechanism such that the remaining companies in the portfolio can

[46]Note that, as previously, to ensure convexity of the buyer's problem, one needs to check that furthermore the subjective loss probabilities $\psi(p(s))$ are convex in s. Furthermore, note that for consistency with the setting of this study we will approximate $\mathbb{P}(N_j(T) = 1) \approx \mathbb{P}(N_j(T) \geq 1)$, i.e. we assume that at most one loss can occur. This is reasonable in practice and for the parameters chosen below, as typically arrival rates of cyber incidents are very low, such that the probability of multiple losses at one policyholder in a single year is negligible (and may even be contractually excluded).

adjust their security in time in order to equally withstand the attack.[47] Denote the subset of the portfolio which is affected by an event after implying the warning mechanism as $\mathcal{S}^*(\ell(\mathbf{s}))$, where $\ell(\mathbf{s}) = (\ell_1, \dots, \ell_{N-1}, \ell_N) = (\ell_{fix}, \dots, \ell_{fix}, \ell(s_I))$.

- **Loss severity:** As in the self-protection case study in Appendix A.5, we assume each loss size to follow a Pareto distribution (with fixed parameters independent of s for a pure self-protection scenario).

Remark 25 (Effect of warning mechanism on almost homogeneous portfolio)**.** *Due to the homogeneity assumption for the structure of the existing portfolio, one needs to distinguish only three cases to understand the functionality of the warning mechanism:*

$$\begin{aligned} (\textit{weak attacks})\ & m \le \ell_{fix} \le \ell(s_I) \implies \mathcal{S}^* = \emptyset, \\ (\textit{medium attacks})\ & \ell_{fix} < m \le \ell(s_I) \implies \mathcal{S}^* \subset \mathcal{S}, \\ (\textit{strong attacks})\ & \ell_{fix} \le \ell(s_I) < m \implies \mathcal{S}^* = \mathcal{S}. \end{aligned}$$

In the case of a weak attack, the first affected firm can always withstand the attack and everyone else can be warned (which is actually unnecessary, as they could also withstand the attack at their established security level). In the case of a strong attack, every company in the targeted set suffers a loss and no warning mechanism can be triggered. In the second (and interesting) case of a medium attack, company N (who is better protected than the homogeneous rest of the portfolio) can withstand the attack and the subset of companies of $\mathcal{S}$ affected after N in the random permutation can be warned in time (essentially, their security level is artificially heightened to $\ell(s_I) > \ell_{fix}$ for this attack).

Remark 26 (Insurance Buyer's Problem)**.** *The optimization problem and its solution for buyer N are analogous to Section 3 and Appendix A.5 with loss probability $p(s)$ as in* (29)*, as he does not consider a portfolio viewpoint; compare Remark 19.*

Remark 27 (Insurer's Portfolio Loss and Optimization Problem)**.** *By the above assumptions, the insurer's portfolio loss X is given by*

$$X = \sum_{j=1}^{N} \Big(\sum_{i=1}^{N_j^{idio}(T)} L_i^{idio} + \sum_{i=1}^{N^{syst}(T)} \mathbb{1}_{j \in \mathcal{S}_i^*(\ell)} L_i^{syst} \Big) =: \sum_{j=1}^{N} X_j,$$

where $N_j^{idio}(T) \sim Poi(\lambda_j^{idio}\, T)$, $N^{syst}(T) \sim Poi(\lambda^g\, T)$, and $L_i^{idio}, L_i^{syst} \sim Pareto(\hat{x}, k)$, i.i.d. $\forall i$ are independent and $\mathcal{S}_i^*(\ell)$ is generated as described above independently for all i. Analogously to Remark 15, the insurer's problem for 'sequential' optimization of contract N is

[47] It is obvious that spreading out resp. ordering attacks from common events over time is actually more realistic than assuming strictly simultaneous losses. Admittedly, the specific warning mechanism is only realistic for certain types of cyber attacks, namely for those that do not immediately "notify" the victim that the system has been compromised or a loss typically stays undetected for some time. This may e.g. be the case for most data breaches (where affected companies may need years until they realize a leak) and malware which lingers or spreads in the system until the attacker uses an opportune moment to cause a business interruption or make a ransomware demand. In this case, only companies which are well-enough protected to realize an attack has been attempted can immediately trigger a warning, whereas compromised victims who do not recognize the breach cannot.

given by

$$\min_{(\theta_N,\beta_N)\in[0,\theta_{R,N}(\beta_N)]\times[\underline{\beta},1]} L^{seq}_{0,N}(\theta_N,\beta_N) = \rho_{0,\ell(\mathbf{s})}(X) - (1+\theta_N)\mathbb{E}_{\ell(s_I(\theta_N,\beta_N))}(X_N) + (1-\beta_N)c(s_I(\theta_N,\beta_N)) - (N-1)(1+\theta_{fix})\mathbb{E}_{\ell_{fix}}(X_1), \tag{30}$$

where $\rho_{0,\ell(\mathbf{s})}(\cdot)$ denotes the insurer's risk measure, dependent on the security levels $\ell(\boldsymbol{s})$ of the portfolio. Note that the last term (premium for the existing portfolio) does not influence the optimization, but serves to check whether the solution complies with the necessary profitability condition $L^{seq}_{0,N}(\theta^*_N,\beta^*_N) < 0$. If the insurer priced the contract individually, she would solve

$$\min_{(\theta_N,\beta_N)\in[0,\theta_{R,N}(\beta_N)]\times[\underline{\beta},1]} L^{ind}_{0,N}(\theta_N,\beta_N) = \rho_{0,\ell(s_I)}(X_N) - (1+\theta_N)\mathbb{E}_{\ell(s_I(\theta_N,\beta_N))}(X_N) + (1-\beta_N)c(s_I(\theta_N,\beta_N)), \tag{31}$$

yielding $(\theta^*_N,\beta^*_N) = (\theta_R(1),1)$; see Section 4 and Appendix A.5.

Example 9. *In Figure 10 and Table 5, we report insightful aspects of the insurer's optimal solution depending on two variables:*

- *Figure 10 shows the optimal share of service cost and the resulting portfolio risk measure for varying p_{syst}, whereby λ^g is adjusted according to $\lambda^g = \frac{\lambda^g_0}{p_{syst}}$ for some $\lambda^g_0 > 0$ constant. This means that the overall expected number of cyber losses from systemic events stays constant, but for larger p_{syst} there are fewer events which affect on average more companies (as opposed to more events to on average smaller subsets for small p_{syst}).*
- *Table 5 shows the optimal parameters (θ^*_N,β^*_N) and the resulting service demand s_I and security level $\ell(s_I)$ of company N for different portfolio sizes N as well as the insurer's resulting total loss and portfolio risk measure.*

It is intuitive from the above construction that the effect of the warning mechanism (and therefore the benefit from company N having higher security standards) increases with both p_{syst} (influencing the expected size of the affected subset for each event) and the portfolio size N. This increases the insurer's willingness to subsidize service by lowering β_N for increasing p_{syst} or increasing N. Precisely this effect is visible in Panel 10a and Table 5. As before, with decreasing β^*_N, the attainable feasible risk loading θ^*_N increases (see Table 5). The higher the subsidy on service cost, the more service company N will purchase within insurance (see Corollary 2), leading to increased security of company N (and therefore a stronger effect of the warning mechanism). Furthermore, the difference in the insurer's portfolio risk between pricing contract N using a portfolio viewpoint and pricing contract N individually again increases (see Panel 10b and the lower part of Table 5). This emphasizes the importance of using a portfolio viewpoint when pricing the additional contract, in particular for large portfolios or portfolios with strong dependence from systemic events.

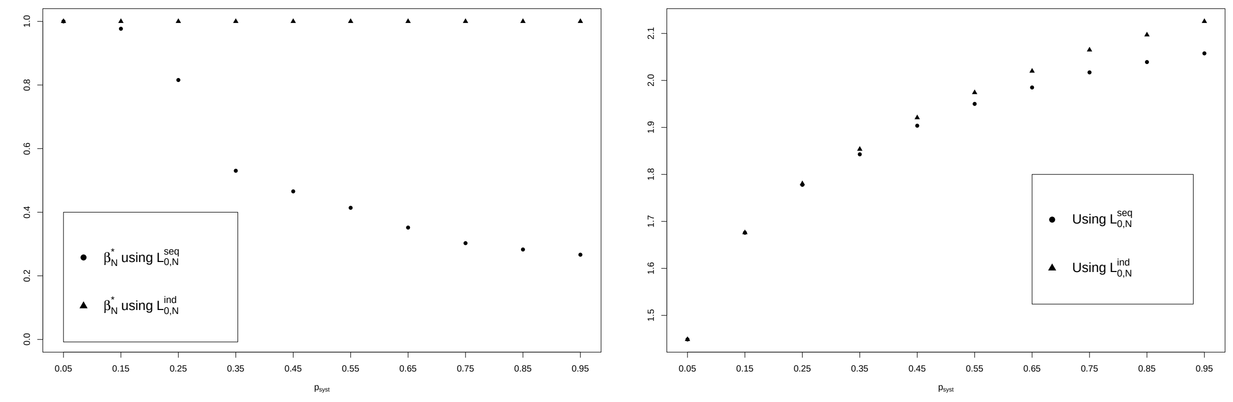

(a) β_N^* as function of p_{syst}. (b) Insurer's optimal portfolio risk $\rho_{0,\ell(\mathbf{s})}(X)$.

Figure 10: Aspects of the insurer's solution in the portfolio case with common cyber events for varying $p_{syst} \in [0.05, 0.95]$ in steps of $\Delta = 0.1$. The parameters for this example are: portfolio size $N = 10$, loss severity parameters $\hat{x} = 1, k = 10$, loss probability parameters $\ell_{fix} = \ell_0 = 0.2, \lambda^{idio} = \frac{\lambda_0^g}{1-\ell_{fix}} = 0.05$, risk aversions $r_0 = 0.7, r_1 = 0.6$, cost parameters $\eta = 0.5, \gamma = 2, \beta_o = 1.1$. The portfolio loss distribution used to approximate the insurer's portfolio risk measure (by numerical integration) is simulated based on 1.000.000 runs.

	No insurance	Insurance (using (31))	Insurance (using (30))				
			$N = 5$	$N = 10$	$N = 25$	$N = 50$	$N = 100$
β_N^*	1.1	1	0.7350	0.4504	0.2076	0.1070	0.0623
θ_N^*	0	1.8055	1.8217	1.8566	1.9408	2.0464	2.1562
s^*	0.0561	0.0971	0.1273	0.1922	0.3501	0.5554	0.7823
$\ell(s^*)$	0.2343	0.2577	0.2739	0.3067	0.3750	0.4461	0.5080
Insurer's Loss (using (31))	0	–	-0.5333	-1.1819	-3.1754	-6.5735	-13.4685
Insurer's Loss (using (30))	0	–	-0.5338	-1.1875	-3.2226	-6.7477	-13.9697
Portfolio Risk (using (31))	0	–	1.0157	1.9467	4.6921	9.1921	18.0933
Portfolio Risk (using (30))	0	–	1.0118	1.9274	4.5883	8.8683	17.2908

Table 5: Aspects of the insurer's solution in the portfolio case with common cyber events with varying portfolio size N. All parameters are as in Figure 10 apart from $p_{syst} = 0.5$ fixed. Due to the (realistically) small rates $\lambda^{idio} = \frac{\lambda_0^g}{1-\ell_{fix}} = 0.05$, the absolute differences of the total loss and portfolio risk measure are only minor (particularly for small portfolio sizes). Therefore, we additionally report the results for another set of parameters (higher rate of systemic events) in Table 6. Note that the qualitative observations do not change, the effects are just more pronounced (as expected).

	No insurance	Insurance (using (31))	Insurance (using (30))				
			$N = 5$	$N = 10$	$N = 25$	$N = 50$	$N = 100$
β_N^*	1.1	1	0.4766	0.3007	0.1650	0.0919	0.0569
θ_N^*	0	0.6079	0.7135	0.8010	0.9807	0.8523	1.4374
s^*	0.1798	0.2960	0.5275	0.7347	1.0986	1.4475	2.0933
$\ell(s^*)$	0.3006	0.3532	0.4374	0.4961	0.5742	0.6293	0.7009
Insurer's Loss (using (31))	0	–	-2.2468	-5.3134	-14.4852	-29.8766	-60.8180
Insurer's Loss (using (30))	0	–	-2.2959	-5.4938	-15.2872	-31.9760	-66.4986
Portfolio Risk (using (31))	0	–	4.0259	7.9876	19.9007	39.6507	78.9922
Portfolio Risk (using (30))	0	–	3.8815	7.5812	18.5390	36.4532	71.1643

Table 6: Aspects of the insurer's solution in the portfolio case with common cyber events with varying portfolio size N. All parameters are as in Table 5 apart from $\lambda^{idio} = \frac{\lambda_0^g}{10(1-\ell_{fix})} = 0.05$, i.e. the ground process rate of systemic events is increased ten-fold. As this increases the occurrence rate of systemic events, it yields more pronounced (but qualitatively analogous) results to Table 5. We remark that we purposely did not choose this as the default example as for higher rates the quality of the approximation in (29) (see the corresponding footnote) deteriorates.
